# Longitudinal phenotyping of maternal antenatal depression in obese pregnant women supports multiple-hit hypothesis for fetal brain development, a secondary analysis of the UPBEAT study

**DOI:** 10.1016/j.eclinm.2022.101512

**Published:** 2022-06-25

**Authors:** Julie Nihouarn Sigurdardottir, Sara White, Angela Flynn, Claire Singh, Annette Briley, Mary Rutherford, Lucilla Poston, UPBEAT consortium

**Affiliations:** a Department of Perinatal Imaging and Health, School of Biomedical Engineering & Imaging Sciences, King's College London, 1st Floor South Wing, St Thomas’ Hospital, London, SE1 7EH, United Kingdom; b Department of Women and Children's Health, School of Life Course and Population Health Sciences, King's College London, 10th Floor, North Wing, St Thomas's, London, SE1 7EH, United Kingdom; c Caring Futures Institute, Flinders University, Sturt Rd, Bedford Park 5042, South Australia, Australia; d Florence Nightingale Faculty of Nursing, Midwifery & Palliative Care, King's College London, 57 Waterloo Road, London SE1 8WA, United Kingdom

**Keywords:** Depression, Antenatal, Pregnancy, EPDS, LCGA, Latent class growth analysis, Neurodevelopment, Brain, Fetus, Exposure, Preterm birth, Ethnic minorities, obesity, Diet, Fatty acids, Glycaemic load, Biomarkers, Inflammation, Diabetes, GDM, Glucose, Phenylalaline, IL-6, infection, placenta, plgf

## Abstract

**Background:**

Maternal antenatal depression is associated with offspring psychological disorders, but obesity is also widely implicated in maternal depression and neurodevelopment. In pregnant women with obesity we explored interrelationships between antenatal depressive symptom trajectories and multiple exposures implicated in fetal neurodevelopment which could explain these associations, as a prelude to exploring associations with infant mental health.

**Methods:**

The UK Pregnancies Better Eating and Activity Trial (UPBEAT) recruited multi-ethnic pregnant women with obesity (BMI >= 30kg/m^2^) between March 2009 and June 2014 from 8 UK sites and 1369 were included to model longitudinal antenatal depressive symptoms from Edinburgh Postnatal Depression Scale (EPDS) scores using Latent Class Growth Analysis. Classes were compared on maternal baseline demography, biomarkers of metabolism, inflammation and placental function, infection, diet and by pregnancy and birth outcomes. Odds ratios, mean differences and 95% Confidence Intervals were calculated using robust auxiliary modelling techniques.

**Findings:**

The chosen model produced four classes: “Not Depressed” (n=575 [42%], “reference”), “Mild” (n=523 [37·5%]), “Moderate” (n=219 [16%]) and “Severe” (n=62 [4·5%]) symptom trajectories. Socio-economic deprivation and ethnic diversity were greater in Severe and Moderate classes. Dietary glycaemic load and saturated fat intake were higher in Severe and Moderate classes (at 17 and 27 weeks). Higher Interleukin-6, glycoprotein acetyls (17 weeks), glucose (34 weeks) and lower placental growth factor (PlGF, 17 and 27 weeks) was found in the Severe class. PlGF was lower in the Moderate class (27 weeks). Infection was least likely in the Not Depressed class across gestation. Risks of preterm birth were associated with Severe depressive symptoms (aOR 3·05[1·11 to 8·36]).

**Interpretation:**

Comprehensive phenotyping exposes important fetal exposures implicated in adverse neurodevelopment, differing by depression class. This study expands substantially on causal models of suboptimal fetal neurodevelopment and offers potential new targets for intervention in obese pregnant women.

**Funding:**

JNS was funded by a PhD studentship from the National Institute for Health Research (NIHR) Biomedical Research Centre based at Guy's and St Thomas’ NHS Foundation Trust and King's College London. UPBEAT was supported by the European Union's 7th Framework Programme (FP7/2007-2013), project EarlyNutrition; grant agreement no. 289346 and the National Institute for Health Research (NIHR) (UK) Programme Grants for Applied Research Programme (RP-0407-10452), Medical Research Council UK Project Grant (MR/L002477/1). Support was also provided by the Chief Scientist Office Scotland, Guy's and St Thomas’ Charity and Tommy's Charity (Registered charity no. 1060508). LP and SLW are funded by Tommy's Charity.

**Research in context**

**Evidence before this study**

We searched PubMed for articles using terms depress* OR EPDS AND pregn* OR antena* AND obes* OR BMI published between January 2015 and December 2020. Whilst reports relate maternal antenatal depression to adverse offspring psychological outcomes, other confounders implicated in adverse fetal neurodevelopment are not incorporated. Studies often evaluate antenatal depression using a single measure and by dichotomisation, rather than longitudinally. None has employed depressive symptom growth modelling in a large and exclusively obese multi-ethnic sample followed by a multi-point assessment of metabolism, inflammation, placental biomarkers, infection risks, dietary intake, obstetric comorbidities and birth outcomes.

**Added value of this study**

This is to date the largest study in a multi-ethnic cohort of pregnant women with obesity which comprehensively describes a range of maternal lifestyle and physiological markers with pregnancy outcomes implicated in adverse fetal neurodevelopment. Our data-driven latent class growth analysis revealed four latent trajectories, two improved as pregnancy progressed although they showed the lowest baseline mean scores. Cross-sectional comparisons of classes on outcomes at three time points offers insight into pertinent relationships during critical windows of fetal development. Capitalising further on methods within the structural equation modelling framework, seldom used in this context, we minimized bias of estimates. We offers reference probabilities and effect sizes which invite further research into biological causal mechanisms.

**Implications of all the available evidence**

Given the multifaceted metabolic profile of pregnancies complicated by obesity, the causal pathways disrupting normal fetal brain development through depression are likely very complex. The study exposes the combinations of exposures which invites reconsideration in future study designs. The between-class differences among those not meeting conventional cut-off scores for depression presented here could imply bias in previously reported associations between antenatal depression and pregnancy/child outcomes in dichotomised designs. Infection incidence was always the lowest among Not depressed women who also had higher socio-economic status. We encourage considering how the socio-demographic profiles and mental health in general relate to outcomes in the mother and exposures for the unborn child.

Alt-text: Unlabelled box

## Introduction

Depression during pregnancy occurs in approximately 17% of women in developed countries^1^ and has been repeatedly associated with increased risk of neurodevelopmental and affective disorders in the exposed offspring.^2^ Suggested causal mechanisms include disturbances in the maternal and the fetal hypothalamicpituitary-adrenal axes through the action of cortisol, mediated via the placenta.^3^^,^^4^ Other plausible pathways and confounders are often neglected such as exposures associated with maternal obesity, occurring in over 20% in UK pregnancies. Indeed, obesity in pregnancy has been repeatedly associated with both maternal depression and autism and attention-deficit hyperactivity disorder (ADHD) in the child.^5^^,^^6^ However, depression and obesity share abnormalities in glucose homeostasis, lipid metabolism and inflammatory mediators and can interact with diet, all of which may influence the developing brain.^7^^,^^8^ Putative mechanisms include the quality of dietary fat intake which can induce a pro-inflammatory state, and deficiencies in omega-3 fatty acids (FA) which have been associated with depression and obesity.^9^

Previous studies addressing these relationships have placed little importance on the interaction between obesity and depression nor with other exposures adverse to fetal neurodevelopment (e.g. infection, diet) and sample size has been a limiting factor.^8^, ^9^, ^10^ Importantly, the frequent use of single-point measurement or dichotomisation of diagnosis oversimplifies the burden of co-morbidities and assumes symptom stability in depression but also of biomarkers in disorders such as gestational diabetes mellitus (GDM), the most common complication of obesity. Such approaches may conceal outcome heterogeneity in “control” pregnancies, possibly introducing bias and undermining optimal antenatal management. In the few studies reporting longitudinal depressive symptom trajectories across pregnancy, samples have been of heterogeneous BMI and include post-partum time points^11^^,^^12^ or suggest the potential role of but do not include biological markers.^13^^,^^14^ There is a need for a comprehensive description of the associations between maternal depression, obesity and fetal exposures in which depression is assessed longitudinally throughout pregnancy.

In anticipation of understanding offspring long-term psychological outcomes, we have addressed these gaps in one of the largest exclusively obese multi-ethnic cohorts of pregnant women from whom longitudinal measures of depressive symptoms, maternal blood biomarkers and a range of clinical and lifestyle factors relevant to fetal neurodevelopment are available. Using Latent Class Growth Analysis, a data driven approach, we modelled the most likely longitudinal depressive symptom profiles, comparing trajectories at median 17, 27 and 34 weeks of pregnancy. We sought demographic profiles and mechanistic pathways that could affect neurodevelopment through depression, and possible novel confounders. This study directly answers a recent call for the integration of cumulative maternal states which have inflammation in common, either acutely (infection) or chronically (obesity, diabetes, pre-eclampsia, low socioeconomic status, depression) and are associated with neurodevelopmental disorders in the child.^15^

## Methods

### Participants

We studied pregnant women with obesity (BMI ≥ 30kg/m^2^) who participated in the UK Pregnancies Better Eating and Activity Trial (UPBEAT)^16^ of a lifestyle intervention in which primary outcome was the prevention of GDM and large-for-gestational age neonates. For trial details and inclusion/exclusion criteria, see the appendix (page 3). UPBEAT complied with criteria set by the Declaration of Helsinki. Ethical approval was given by the National Health Service (NHS) Research Ethics Committee (09/H0802/5) and all participants provided informed consent.

Questionnaires were completed and blood samples collected at three antenatal visits from 1369/1490 women who gave birth to live neonates and completed at least one Edinburgh Postnatal Depression Scale (EPDS).^17^ 935/1369 (68·3%) participants provided all three EPDS questionnaires. There were no demographic differences between women who were included and those with confirmed live births who were excluded (n=121/1490, appendix page 4).

### Antenatal depression

Depressive symptoms were evaluated using the Edinburgh Postnatal Depression Scale (EPDS),^17^ a freely accessible 10-item self-reported screening tool of depression, validated in pregnancy in multiple languages. The total sum score ranges from 0-30 points. Major depression is suspected at the cutoff of 14/15 and minor at 12/13 points.^18^ Responses were provided by 1300 women at median[IQR] 17[16 to 17] weeks gestation (baseline visit), 1156 women at 27[27 to 28] weeks (visit 2) and 1004 women at 34[34 to 35] weeks (visit 3)(see appendix page 5). Women presenting as high-risk on the EPDS or those scoring high on the *self-harm* item were referred to NHS perinatal mental health services as according to local protocols.

We first interrogated whether (latent) groups of women could be identified based on the antenatal trajectories as measured by their total EPDS scores. Then we explored whether heterogeneity in trajectories was also reflected in heterogeneity in maternal and neonatal factors known to influence neurodevelopment based on the literature.

### Variables and outcomes

Baseline demographic variables and anthropometric measurements were recorded at median 17 weeks. Other than providing demographic characterisation, demographic variables were chosen based on reported associations with obesity, mood disorders and/or offspring outcomes such as socio-economic status (SES), asthma and smoking. Anthropometric measures were included as a more informative reflection of maternal adiposity compared to BMI alone and in order to derive a latent “Obesity” factor. This latent obesity factor helps minimise the confounding adiposity-induced effects (i.e.”meta-inflammation” and metabolic disturbance) on outcomes when the depressive status is the main factor of interest.

Dietary intake, was evaluated by the food frequency questionnaire (FFQ) for the previous month at 17 weeks and 27 weeks. An automated program was developed to transform data from the FFQs into nutrient intakes as previously described.^16^^,^^19^

Blood samples taken at each visit were analysed with standard laboratory assays or a targeted Nuclear Magnetic Resonance (NMR) metabolome and are detailed in the appendix (page 7) and previously.^20^ To exclude active infection at sampling, logistic regression was used to compare classes at each visit and no difference in CRP >10mg/L (clinical cut-off) was found (see appendix page 37).

Analysed as a binary event, clinical infection was self-reported for the period prior to each visit, prespecified for respiratory infection/flu, lower urinary tract infection (UTI), pyelonephritis, gastroenteritis, vaginal candida (VC), suspected VC or “other”.

Other pregnancy outcomes included GDM (defined according to International Association of Diabetes and Pregnancy Study Groups [IADPSG] guidelines) and pre-eclampsia (only included if not comorbid with GDM), total gestational weight gain at 34 weeks and antenatal admissions to hospital. Birth outcomes such as prematurity and delivery complications were included. Details on data collection, availability and definitions of the above variables are in the appendix pages 5-17 and missing data strategy on page 17.

### Statistical analysis

#### Latent class growth analysis (LCGA)

Reporting of the LCGA followed the *Guidelines for Reporting on Latent Trajectory Studies (GRoLTS)* Checklist.^21^ LCGA helps detect the presence of subgroups of individuals which share a pattern of scores and change over time and assigns them probabilistically to latent classes.^22^ Unlike repeated measure ANOVA, it provides an intercept and a rate of change (slope)^23^ and retrieves classes in a data-driven fashion, here from the sum EPDS scores at each time point. *Mplus v8·3*^24^ was used to generate all the models described and the outputs were integrated with the R package *MplusAutomation*.^25^ To handle missing data, *Mplus* implements full information maximum likelihood (FIML) under the assumption of missing at random (MAR) and the robust maximum likelihood (MLR) estimator dealt with non-normal distributions of EPDS scores. One to five-class models were generated and fit indices and criteria were assessed to select the best fitting model such as Aikaike Information Criterion (AIC) Bayesian Information Criterion (BIC), sample-size Adjusted BIC (aBIC) where the smallest value is preferred. We also evaluated the entropy (measures the accuracy of group assignment and membership) where entropy =1 reflects perfect classification.^26^ Each run was checked that it had reached global maximum i.e. the best log-likelihood value had successfully replicated. Further description of other longitudinal models generated, model selection and interpretation in the appendix (page 19).

#### Class comparisons

From the chosen model we first compared the classes against a reference class (see below) across baseline socio-demographic characteristics to determine their profiles and then on outcomes of interest to fetal development to inspect heterogeneity. To do this we employed the AUXILIARY option in *Mplus* and the three-step approach^26^ considered superior to comparing classes (by e.g. ANOVA) based on most probable participant class membership. This reduces the bias attributed to the LCGA since participant classification measurement error (i.e. fractional classification to other classes) is accounted for.^27^ It performs unadjusted and adjusted multinomial/logistic regressions for categorical outcomes and pairwise comparisons of means for continuous outcomes. This refers to all the comparisons between classes on demography, diet, blood markers, infection and pregnancy/birth as the outcomes measures when classes are the independent variable. There MLR estimator provided robust estimates and standard errors.

To control for class-wise baseline differences and assess the specific influence of maternal mental health, adjustment for maternal age, nulliparity (vs multiparity), White ethnicity (vs non-White, dichomotmised from the four ethnic groups at baseline to facilitate model convergence and interpretation), latent obesity and latent SES served as covariates in analyses of diet, blood biomarkers, infection, pregnancy and birth outcomes. The intervention effect was also included as a covariate when outcomes were measured after the baseline 17 week visit. Latent factor modelling of obesity and SES was done to reduce measurement bias of these constructs otherwise introduced in conventional regressions using observed proxy measures. The latent “obesity” variable was indicated by sum of skinfold, waist and hip circumferences and BMI (all continuous) and a latent “SES” variable by income, index of multiple deprivation, and highest education attained (all ordinal), see appendix page 28.

Standard deviation unit difference in class means or Odds Ratio [OR, 95%CIs] against the reference class are presented. When variables are categorical/binary, we provide the probability (%) of response for each class. If CIs excluded 1 or 0 for the ORs and mean differences, respectively, the effect was viewed as significant. Blood analyte concentrations and dietary variables (continuous) were standardized and log transformed if non-normally distributed. We visually report the main effects of covariates on blood and dietary outcomes as these are underreported in the literature but may have clinical and epidemiological value. The interaction of covariates with classes was evaluated by within-class regressions. To improve estimation of all covariate effects, the covariance of latent SES and latent obesity was included in regression models since theoretically plausible. Methods are further described in appendix (page 24). In a sensitivity analysis, we excluded blood samples from 120 participants taken at 27 weeks from one study site which deviated from the UPBEAT processing protocol (analytes were retrieved from a 1h OGTT sample rather than fasting blood).

### Role of the funding source

The funding source had no role in the study design, data collection, analysis, interpretation, writing of this report nor in the decision to submit the manuscript.

All authors had full access to all the data in the study and accept responsibility to submit for publication.

## Results

Characteristics for the study sample (n=1369) at study entry (baseline) are in Table 1. The median [IQR] EPDS scores at 17, 27 and 34 weeks were 6[3 to 10], 5[2 to 9] and 5[2 to 8] and frequencies for scores >= 13 points threshold were 157/1300 (12·1%), 118/1158(10·2%) and 82/1005(8·2%) respectively. 693 (50·62%) women were originally randomized to the intervention and 676(49·38%) to standard care.

**Table 1:** Entry characteristics at first visit in the whole sample of 1369 obese pregnant women.

		N=1369
**Age(years)**		30·5 (5·47)
**Main ethnicity**	White	857 (62·6%)
	Black	351 (25·6%)
	Asian	85 (6·2%)
	Other	76 (5·6%)
**UK born**	No	447(32·6%)
	Yes	922(67·3%)
**Index Multiple Deprivation**	Least Deprived	54 (3·9%)
	2nd quintile	90 (6·6%)
	3rd quintile	155 (11·3%)
	4th quintile	462 (33·7%)
	Most deprived	604 (44·1%)
**Highest education attained**	None	57 (4·2%)
	GCE (or equivalent)	224 (16·4%)
	Vocational qualification	333 (24·3%)
	A level (or equivalent)	214 (15·6%)
	First degree	365 (26·7%)
	Higher degree	176 (12·9%)
**Job situation**	School	60 (4·4%)
	In paid/self employement	894 (65·3%)
	Looking after home or family	240 (17·5%)
	Not doing paid work	147 (10·7%)
	Government scheme training	6 (0·4%)
	Permanently unable to work	9 (0·7%)
	Retired	1 (<0·1%)
	Doing something else	12 (0·9%)
**Parity**	Multiparous	771 (56·3%)
	Nulliparous	598 (43·6%)
**Child(ren) in household age < 2yrs**	0	1182 (86·3%)
	1+	187 (13·7%)
**Living with partner**	Yes	1060 (77·4%)
	No	309(22·6%)
**Type of accommodation**	Own house/flat	443 (32·4%)
	Temporary	6 (0·4%)
	Family/Friends rent free	80 (5·8%)
	Private rental	413 (30·2%)
	Council rental	359 (26·2%)
	Other	29 (2·8%)
**Income at entry (gross)**	< £12,688	247 (18·0%)
	£12,688 - £17,628	159 (11·6%)
	£17,629 - £23,452	112 (8·2%)
	£23,453 - £32,500	169 (12·3%)
	> £32,500	479 (35·0%)
	Prefers not to answer	203 (14·8%)
**Anthropometrics**	BMI	35·0 (32·8, 38·5)
**Folate supplement**	Daily	800(58·4%)
	Less than daily	91(6·6%)
	None	478(34·9%)
**Asthma**	No	1113(81·3%)
	Yes	256(18·7%)
**Smoking**	No	1279(93·4%)
	Current	90(6·6%)
**Randomisation**	Controls	676(49·38%)
	Intervention	693 (50·62%)

Continuous variables presented as mean(standard deviation) or median (interquartile range) if non-normally distributed. Asthma was coded as 'yes' if it had been diagnosed by a medical practitioner. Missing: Index of Multiple Deprivation=4, accommodation=39. BMI: Body Mass Index.

### LCGA model estimation

**Figure 1:**
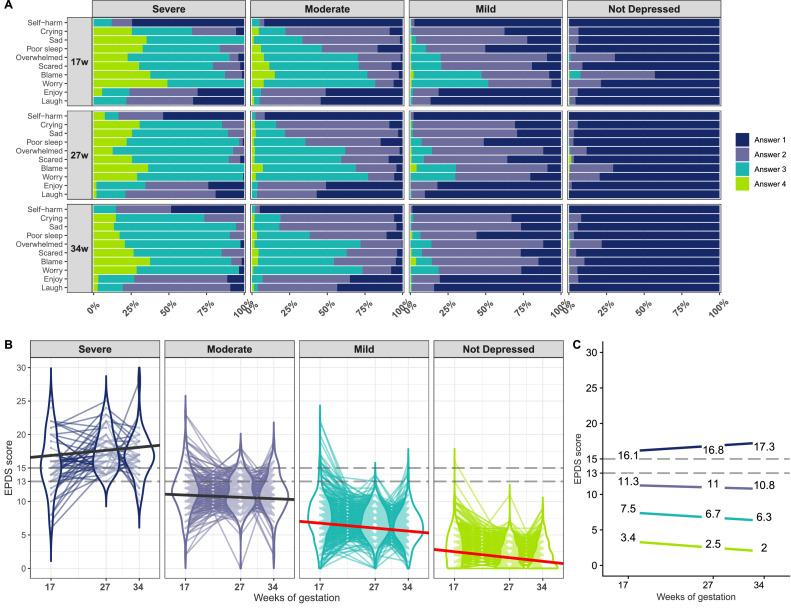
EPDS item response probabilities and total score trajectories of the 4-class model. A. Item response probabilities were estimated for each class where Answer 4 is the highest scoring response (i.e. highest severity/frequency). B. Observed EPDS scores split according to most likely class membership following LCGA using all data available (n=1369). Each line refers to a subject and violin plots illustrate the score distributions. Solid black/red lines are within group average trajectories (slopes different from 0 at p<0·05 are in red). Classes were defined as ‘Not Depressed’ and improving (n=575, 42%; mean intercept = 3·4 points (SE=0·22, p<0·001); slope =-0·9 points (SE=0·1, p<0·001)), ‘Mild’ and improving (n=513, 37·5%; intercept = 7·5 (SE=0·5, p<0·001); slope =-0·74 (SE=0·20,p<0·001)), ‘Moderate’ and stable (n=219, 16%, intercept = 11·3 (SE=0·55, p<0·001); slope = -0·34 (SE=0·46, p=0·47) and ‘Severe’ and stable (n=62,4·5%; intercept= 16·1(SE=0·87, p<0·001); slope =0·77 (SE=0·56, p=0·174)). C. Estimated mean score at each time point in each class, colors same as in B. Dashed lines in B. and C. are references to the 12/13 and 14/15 cutoff scores for all and major depression respectively (Murray et al., 1990). EPDS, Edinburgh Postnatal Depression Scale.

One to five class models were generated and all reached convergence and the best log-likelihood was replicated in each solution. Model estimation, selection criteria and summaries are provided in the appendix (page 19-24). The 1-class model had the worst fit (highest BIC/AIC/aBIC values) suggesting heterogeneity in depressive symptom trajectories from 17 to 34 weeks gestation. A 4-class model was accepted and described by class in Figure 1. Classes were labelled based on their means symptom severity and slope as: “Not Depressed” (n=575,42%),“Mild”(n=513, 37·5%),“Moderate”(n=219, 16%) and “Severe”(n=62, 4·5%). The “Not Depressed” class provided the opportunity to obtain a reference “control” class against which the other three classes were compared. The complete case sample (n=935) also yielded a 4-class model although the main difference was a significant slope (worsening symptoms) in the Severe group (appendix page 23).

#### Baseline characteristics by class

**Figure 2:**
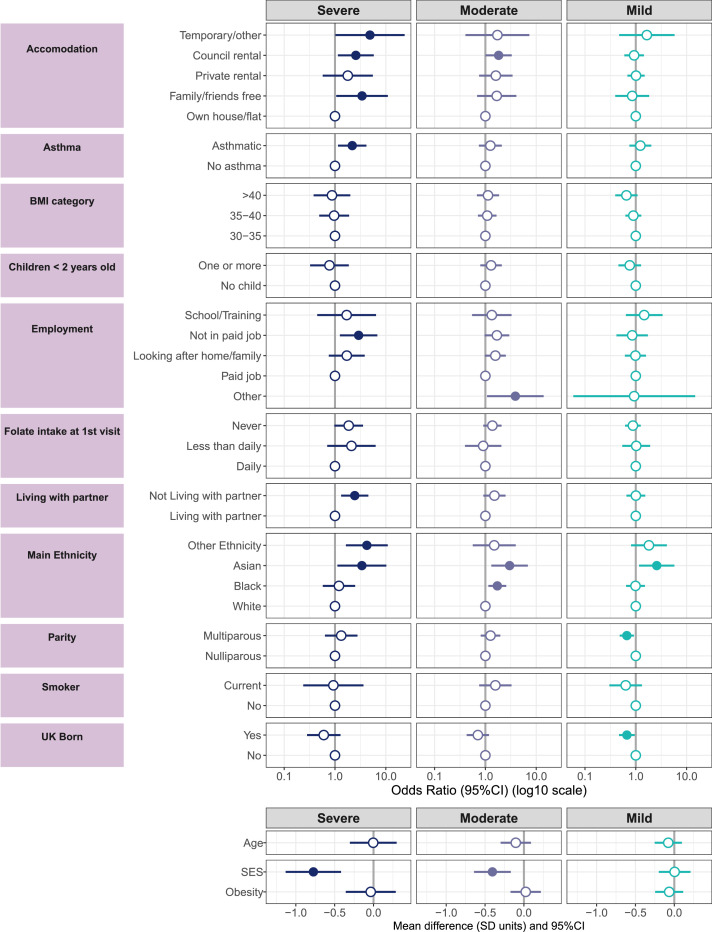
Comparisons against the Not Depressed class on baseline characteristics. The participant counts based on the most likely class membership was n=62 (Severe), n=219 (Moderate), n=513 (Mild) and n=575 (Not Depressed, reference). Odds ratio below 1 indicate the latent group's odds for that category relative the reference category are lower against the same odds in the Not depressed class. Dots are filled if the OR CIs exclude 1 or the mean difference CIs exclude 0, which is interpreted as significant. Measurement error attributed to the LCGA was taken into account. SES and Obesity are latent factors (see appendix page 28). Missing: Index of Multiple Deprivation n=4, Accommodation n= 39. SES: socioeconomic status.

Comparisons on baseline factors against the Not Depressed class are represented in Figure 2 and appendix pages 25-27. Women in the Severe class were more socio-economically deprived (standardized Mean Difference -0·77[-1·13 to -0·42]), more likely to be of Asian or “other” ethnicity (than white), to live in temporary/council rental or with family rather than their own property, not living with their partners, unemployed, and asthmatic. Women in the Moderate group were more socio-economically deprived (sMD -0·41[-0·64 to -0·17]), more likely Asian or Black than White, and living in council rentals than their own homes. Women in the Mild group were socio-economically comparable (sMD 0·00[-0·20 to 0·21]) to the Not Depressed women but more likely to be Asian, born outside the UK and less likely multiparous. There were no differences in latent obesity or age between classes. We found probabilities of women being in the intervention group similar (Severe: 53,1%, Moderate: 48·9%, Mild: 52·5% and Not Depressed: 49·4% (compared to the Not Depressed class Severe: OR=0·62 [0·37 to 2·16], Moderate: OR=0·64 [0·22 to 1·57], Mild: OR=0·79 [0·21 to 1·62].)

#### Diet

**Figure 3:**
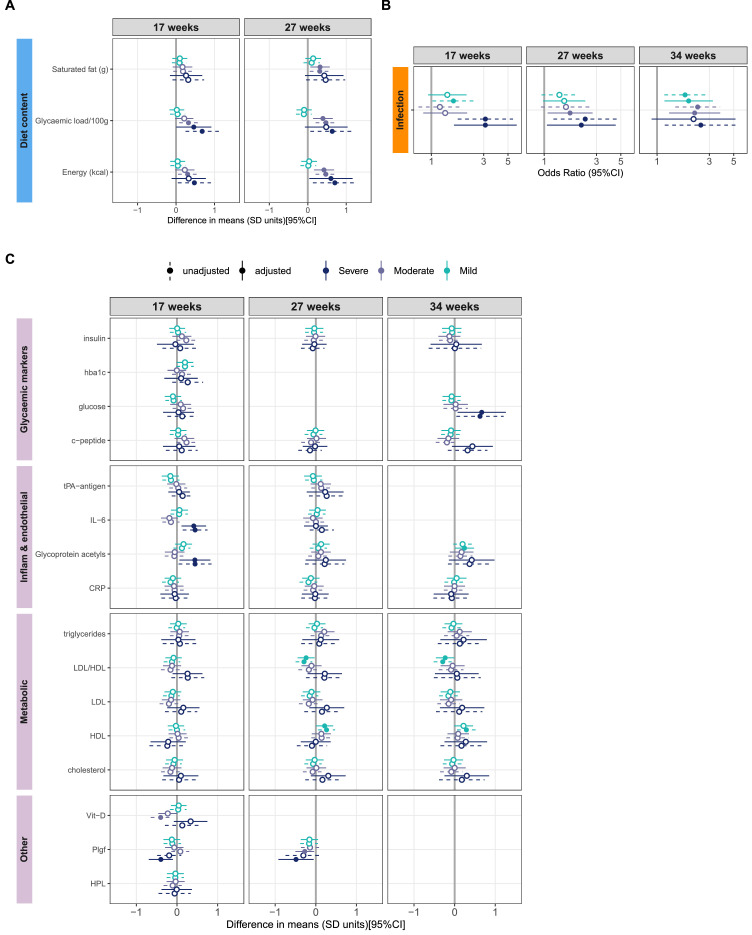
Classes compared against the Not Depressed class on their macronutrient intake (A), self- reported infection prior to each visit (B) and blood biomarkers (C), each presented unadjusted and adjusted for maternal age, nulliparity, white ethnicity (vs non-white), latent SES and latent Obesity, and the intervention effect at 27 and 34 weeks only. Filled circles: CIs exclude 0 for the mean differences in A and C or 1 for the ORs in B, interpreted as significant. CRP: C-reactive protein, DHA: Docosahexaenoic acid, hba1c: glycated haemoglobin, HDL: high-density lipoproteins, HPL: Human Placental Lactogen, IL-6: Interleukin- 6, LDL: low-density lipoproteins, Plgf: Placental growth factor, tPA-antigen: Tissue plasminogen activator antigen.

Comparisons on dietary intake are shown in Figure 3A and composition (% Energy) in appendix pages 29-32. There were notable associations between depression classes and dietary intake. After adjustment for baseline factors, at 17 weeks, women in the Severe depression class reported higher glycaemic load (per 100g, adjusted sMD: 0·46[0·00 to 0·92]) and lower protein composition (% Energy, asMD: -0·50[-0·85 to -0·15]). At 27 weeks they showed higher energy intake (kcal, asMD: 0·60[0·04 to 1·17]). For the Moderate group, glycaemic load (per 100g) and energy intake (kcal) was higher in unadjusted comparisons only. However, at 27 weeks, these women reported higher glycaemic load, energy intake (kcal, asMD: 0·39[0·14 to 0·65]) and saturated fat intake (grams, asMD 0·32[0·07 to 0·57]) than the Not Depressed women in adjusted comparisons. The only difference between the Mild and Not Depressed classes was at 27 weeks where diet intake in total fats and saturated fats was higher and carbohydrates lower (all as % of total energy) for the Mild women, after adjustment.

Age, SES and White (vs non-White) ethnicity had overall the strongest effects on the diet at 17 weeks. The intervention, SES and White ethnicity had the largest main effects on diet at 27 weeks (appendix page 33). To explore interactions, within-class regressions suggested that the effect of the intervention on reducing saturated fat and glycaemic load was evident in the Mild and Not Depressed classes only. Heterogeneous effect of age, White ethnicity and SES across the classes also suggest interactions of covariates with class membership on dietary outcomes (appendix page 34).

#### Blood biomarkers

Figure 3C presents results on inflammatory, metabolic and placental markers. For results on adipokines, amino acids and fatty acids see appendix pages 40 and 35-37.

At 17 weeks the Severe class had significantly higher adjusted IL-6 (asMD:0·41[0·11 to 0·72]), glycoprotein acetyls (0·44[0·06 to 0·82]), phenylalanine levels (0·40[0·04 to 0·76]) and lower PlGF (-0·40[-0·70 to -0·10]) than the Not Depressed group. At 27 weeks, phenylalanine in the Severe class remained higher and PlGF lower (-0·27[-0·50 to -0·04]). At 34 weeks, these women had higher adjusted blood glucose (0·67[0·07 to 1·26]) and lower Omega-6/Omega-3 ratio (-0·46[-0·86 to -0·07]). We noted only a trend for increased absolute FA subtypes concentration in the Severe class across pregnancy. At 27 weeks Moderate women presented lower PlGF (-0·27[-0·50 to -0·04]) and higher isoleucine and higher alanine in adjusted comparisons. There were no differences between the Mild and Not Depressed classes amongst blood markers at 17 weeks. However in adjusted comparisons at 27 weeks, women in the Mild class showed lower LDL/HDL ratio (-0·24[-0·45 to -0·03], with accompanying higher blood HDL), lower Omega-6/ Omega-3 ratio and higher degree of blood unsaturation and proportion of Omega-3/Total FA. At 34 weeks, they showed higher glycoprotein acetyls, a higher degree of unsaturation (0·23[0·03 to 0·44]), lower Saturated FA/Total FA proportion and lower LDL/HDL ratio (-0·24[-0·45 to -0·03]) compared to the Not Depressed women. Sensitivity analyses at 27 weeks generated small changes i.e. adjusted triglycerides was higher in the Moderate class and PlGF was no longer lower in the Severe and Moderate class (appendix page 46).

**Figure 4:**
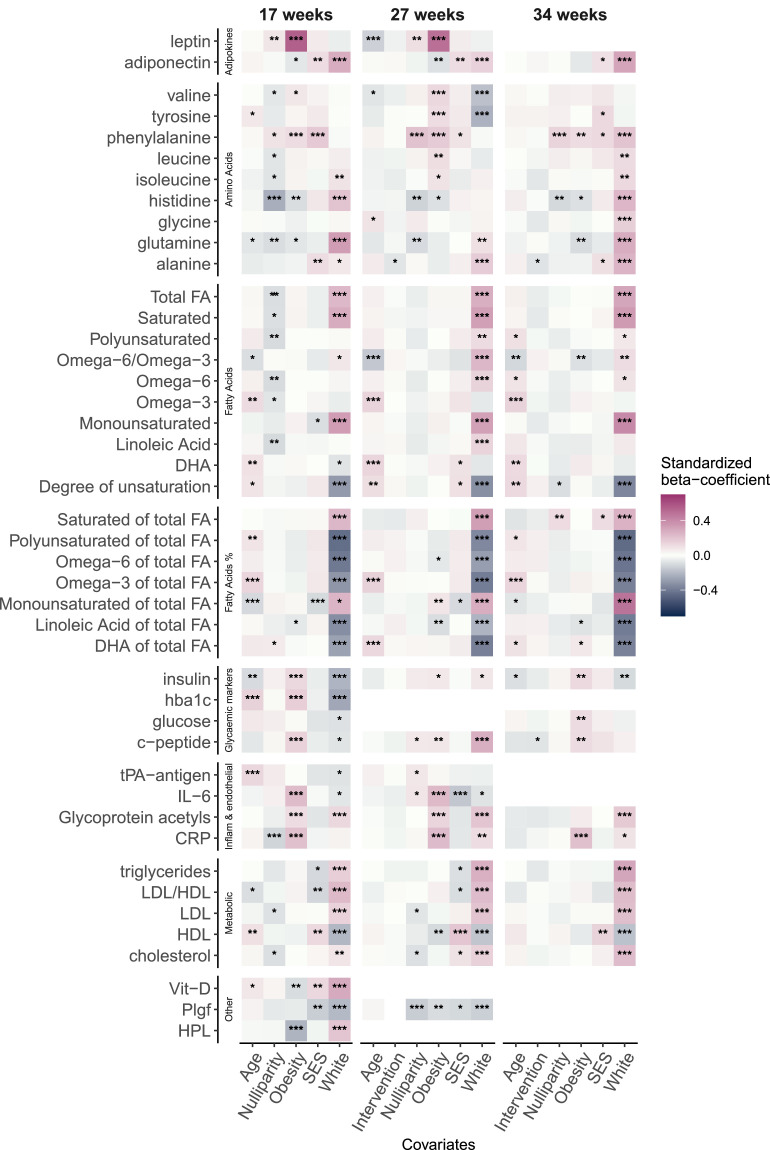
Main effects of maternal covariates included in multiple regressions of adjusted analyses with each blood marker as an outcome, at 17, 27 and 34 weeks gestation. The correlation of latent SES and latent Obesity was included in each model. Variables significantly associated with metabolites are annotated ‘*’:p<0·05,’**‘:p<0·01,’***’p<0·001. CRP: C-reactive protein, DHA: Docosahexaenoic acid, FA: Fatty Acid, hba1c: glycated haemoglobin, HDL: high-density lipoproteins, HPL: Human Placental Lactogen, IL-6: Interleukin- 6, LDL: low-density lipoproteins, Plgf: Placental growth factor, SES: socioeconomic status, tPA-antigen: Tissue plasminogen activator antigen.

Overall, white ethnicity appeared to have the strongest effect on blood biomarkers (Figure 4) followed by latent obesity and SES. The effect of the UPBEAT intervention on blood markers at 27 and 34 weeks was not evident when including all other covariates.

#### Pregnancy outcomes

The Severe class was 3·11 times more likely (aOR, a95%CI: 1·61 to 6·00) than the Not Depressed class to report an infection occurring prior to 17 weeks (46·5% probability vs 21·9%) and 2·18 times [1·06 to 4·49] at 27 weeks (40·6% vs 22·4%). Infection in the Moderate class was 1·72 times [1·07 to 2·76] and 2·20 times [1·29 to 3·77] more likely at 27 and 34 weeks respectively. The Mild class was 1·94 times [1·17 to 3·23] more likely at 34 weeks, see Figure 3B and appendix (page 48). There were no differences in odds of GDM and pre-eclampsia between classes (appendix page 49). However, we found that the OR of missing a GDM diagnosis (i.e. missing OGTT) was 2·9 times more likely for the Severe class (a95%CI: 1·32 to 6·36; 31% probability vs a 13·2% probability), but odds across the other classes were not different. Moreover, the Severe class was 2·69 times more likely (a95%CI [1·18 to 6·13]) than the Not Depressed class to be admitted to hospital during pregnancy (probability 20·60% vs 7·60%, appendix page 49). After adjustment, the Severe class gained a mean 2·64kg more [0·63kg to 4·64kg] than the Not Depressed women at 34 weeks (appendix page 48).

Preterm birth (<37 weeks) was 3·05 times more likely for the Severe class (a95%CI: 1·11 to 8·36) with probability of 14·6% compared to 2·9% in the Not Depressed. The OR[95%CI] for the Mild class was 3·07[1·22 to 7·72] with a 8·4% probability but no longer significant after adjustment (aOR: 1·84[0·81 to 4·18]). To differentiate from iatrogenic premature birth (i.e. physician initiated), the OR of spontaenous preterm birth or premature rupture of membrane was 4·63[1·36 to 15·73] for the Severe and 3·19[1·11 to 9·22] for the Mild class, this difference was not significant after adjustment. Odds of induction of labour, Caesarean section, blood loss at birth >1000ml and NICU admission and gestational age at birth (days) were similar between classes (appendix pages 50 and 51). Large-for-gestational-age infants (WHO definition) was less likely for the Mild class (OR: 0·51[0·30 to 0·88]) but no longer significant after adjustment (aOR: 0·54[0·29 to 1·02]). Odds of small-for-gestational age infants were similar across classes.

## Discussion

This study is the first to report the multiple exposures related to maternal antenatal depression and obesity which may adversely influence fetal neurodevelopment. While we cannot infer causality here, the analysis identified several important lifestyle factors including diet and biomarkers which could theoretically lie on the causal pathway of the reported association between maternal obesity/depression and adverse offspring neurodevelopment. In the context of psychopathological risks factors, it highlights the complexities of causal models within the framework of the Developmental Origins of Health and Disease.

The Severe and Moderate depression classes reported a modest increase in absolute glycaemic load and saturated fat intake compared to Not Depressed women, independent of socio-economic factors. Overall, the model indicates a dose-dependent effect of maternal symptom severity and reported dietary intake not previously described using these methods but consistent with depression being associated with poorer diet.^28^ The implication for the fetus lies in the known effects of maternal high-fat and high-sugar diets on neurodevelopment.^29^ Although there was only a trend to higher absolute FA subtypes concentrations in the blood of the Severe class, there was evidence for a lower LDL/HDL and higher blood FA unsaturation in the Mild class. Additionally, lower dietary protein intake (% E) in the Severe and Moderate classes could suggest heterogeneity in serotonin precursor availability within the fetal brain, necessarily derived from maternal dietary protein. The implication of higher phenylalanine in the Severe class (notably found in artificial sweeteners) is unclear since relationships with fetal neurodevelopment have exclusively been studied in the context of phenylketonuria, when concentrations are very high.^30^

The heightened concentration of the pro-inflammatory cytokine IL-6 in the Severe class is consistent with it being a biomarker of depression.^31^ Glycoprotein acetyls was also higher and has recently been identified as a marker of chronic inflammation. It increases throughout pregnancy^32^ and has been recently implicated in antenatal depression symptomology.^33^

Importantly, antenatal maternal immune activation (MIA) has been associated with increased risk of offspring psychiatric disorders.^34^ We reported that recurring maternal infections were common and probability of infection was least in the Not Depressed class. Maternal antenatal infection, including UTI, has frequently been linked with offspring outcomes of autism and depression.^35^ Animal models have shown that maternal immune activation increases inflammatory responses in the fetal brain and subsequent offspring behavioural abnormalities, where this association was mediated by placental IL-6.^36^ The degree and extent to which MIA affects the fetal brain will vary by gestational age given the known critical windows in neurodevelopment and placental permeability being highest early in pregnancy.^37^ Whereas others have frequently implicated cortisol in the mechanisms linking maternal depression and offspring psychological outcome, our results emphasise the need to include infection and inflammation as confounders or interacting factors.^38^

Of interest, there were no differences in odds of GDM and PE between the depression classes, despite both having been implicated in adverse neurodevelopmental outcomes.^39^ However, the higher probability of the Severe class missing their GDM diagnostic test suggests under-diagnosis and could explain their higher glucose at 34 weeks and may constitute an additional risk factor for adverse offspring outcomes. Of potential relevance to this high rate of failure to attend, mental and social problems have been previously identified as barriers to attending OGTT.^40^

The Severe class were threefold more likely to deliver prematurely. Prematurity is a recognised population risk factor for neurodevelopmental disorders and our findings agree with the literature associating preterm-birth with depressed/stress pregnancies, and higher IL-6 concentrations.^41^ The 2·7 fold risk of hospital admission has not previously been reported in relation to depression in obese pregnancy but has been implicated in risks of autism in the child if due to maternal viral infection.^42^ Finally, PlGF is an angiogenic factor which plays a key role in placental development and growth and was lower in the Severe and Moderate classes. This suggests poorer placental function and a potential impact on nutrient availability to the fetal brain.

This is, to our knowledge, the first study to show a cumulative increase in socio-economic disparity and ethnic diversity associated with a stepwise increase in longitudinal depressive symptoms in obese pregnant women. The observation that women with severe symptoms had the lowest SES, were less likely to be living with their partner and more likely unemployed, reflects other reports^1^^,^^43^ including a small UK study of women with Major Depressive Disorder.^41^ These parallels further strengthen the validity of our longitudinal phenotyping of depression in an unselected sample. Socio-economic deprivation is strongly linked to adverse pregnancy outcomes such as preterm birth and our observation that Severely depressed women had less secure accommodation is consistent also with findings that extreme housing insecurity increase risks of preterm birth by 73%.^44^ More ethnic diversity in all classes other than the Not Depressed class could reflect recent reports that UK ethnic minorities are less likely to access mental health services, to be referred to primary/secondary care^45^ or to be asked about their mental health during pregnancy.^46^

The primary strengths of our study lie in the advantages of the LCGA and our implementation of robust methods within the structural equation modelling framework, the comprehensive data available and the large sample size in an exclusively obese cohort. This is, to our knowledge, the most extensive characterisation of heterogeneity of outcomes across longitudinal antenatal depressive symptom profiles derived from a screening tool such as the EPDS, providing reference estimates against a clear low-risk group for three antenatal time points. It emphasises the importance of repeated measures in establishing stability of the fetal exposures in obese pregnancies.

The study was limited by the trial exclusion criteria and the intervention which bias representation. We also cannot exclude any effect of medication eg anti-depressants or antibiotics on the biomarkers of interest. No adjustment for multiple testing was applied here which could be viewed as a limitation and with nearly 200 outcomes we would expect 1 in 20 results to be significant due to chance, however our analyses were based on comparisons of 95% CIs and independence of many outcomes is unlikely. Indeed, it is well established that many biomarkers (e.g., IL-6, CRP) are functionally related. Providing CIs adds value in exploring a large array of fetal exposures and the size and directions of effects likely to be valuable in generating new hypotheses^47^ or for future smaller studies using Bayes statistics and priors. Nevertheless, the retrospective and explorative nature of LCGA may lead to underpowered analyses in the smallest class (Severe). Additionally, no epigenetic/genetic variables were considered, nor the contribution of paternal mental health. We cannot rule out bias from a retrospective self-report of diet, nor of infection although the former may be substantiated by the higher gestational weight gain in the Severe class and latter was predefined according to standard clinical guidelines. Analyses into causal mechanisms with outcomes were beyond the scope of this study but will follow for offspring psychological outcomes.

Our findings have several implications. Exploring trajectories of antenatal depression reveals improvement in symptoms in latent groups of women with already low and mild baseline risks which single-measure and dichotomised designs cannot detect. Further, fluctuations in symptoms at different antenatal periods may lead to an overestimation of cases of depressed women in these designs. The Moderate class scored on average below the conventional threshold for suspected depression and the lifestyle intervention effect on diet in this class and the Severe classes was not evident, as demonstrated by the within-class regressions. Overall, our latent modelling implies thus that the assumption of homogeneity in subthreshold individuals (i.e. in dichotomised studies) is not upheld, since we uncovered heterogeneities across classes on a wide array of outcomes, which would bias statistical analyses if ignored. Moreover, a reappraisal of the influence of a maternal lifestyle intervention in this RCT, or in any future intervention trial among those experiencing low mood, is warranted in the context of prevention of suboptimal fetal neurodevelopment. Finally, the sociodemographic profiling provided could have clinical value in detecting at-risk pregnant women and promote policies on improving access to care, especially among ethnic minorities.

To conclude, this study reveals that obesity, now highly prevalent amongst women in antenatal care worldwide, complicates causal modelling between maternal depression and offspring short and long-term outcomes. By providing cross-sectional estimates at three time points, our study invites the generation of new hypotheses into a multifactorial and multi-hit model of transgenerational transfer of psychopathological risk.

## Contributors

JNS: conceptualisation, literature search, data analysis, visualisation, writing- original draft, review and editing. LP: resources, project administration, funding acquisition, supervision, review and editing. MR: resources, supervision, review and editing. SW: data curation, resources, review and editing. AF/CS: data curation, resources, review and editing. AB: project management, data acquisition, data curation, review and editing. JNS and LP have verified the underlying and all authors had access to the underlying data and accept responsibility for submission. All other authors critically reviewed and approved the final version.

## Data sharing statement

Anonymised participant data and data dictionary are made available upon request to and approval of the UPBEAT Scientific Advisory Committee (www.medscinet.net/upbeat/).

## Declaration of interests

All authors declare that there is no conflict of interest associated with their contribution to this manuscript.

## Appendix Supplementary materials

Image, application 1
