# Supplementary material for: Longitudinal phenotyping of maternal antenatal depression in obese pregnant women supports multiple-hit hypothesis for fetal brain development, a secondary analysis of the UPBEAT study
Source: eClinicalMedicine. 2022 Jun 25;50:101512. doi: 10.1016/j.eclinm.2022.101512 (PMC9241104; doi:10.1016/j.eclinm.2022.101512)
Supplement: Supplementary file 1 [file mmc1.pdf]

Appendix for ‘Longitudinal phenotyping of maternal antenatal depression in obese pregnant women supports multiple-hit hypothesis for fetal brain development, a secondary analysis of the UPBEAT study.’

Julie Nihouarn Sigurdardottir et al.

Contents

1 Participants exclusion and inclusion in UPBEAT **3**

2 Variables **5**
2.1 Edinburgh Postnatal Depression Scale (EPDS) 5
2.2 Demographics/Baseline variables 5
2.3 Anthropometrics 5
2.4 Diet 6
2.5 Blood Markers collection, availability and processing 7
2.6 Pregnancy complications 10
2.6.1 Obstetric 10
2.6.2 Infections 12
2.6.3 Other 12
2.7 Birth and neonatal outcomes 15

3 Missing data **17**

4 Latent Class Growth Analaysis - LCGA **19**
4.1 Criteria 19
4.2 Model selection and interpretation 19
4.3 LCGA all data vs complete data 22
4.4 LCGA vs GMM 24

5 Group comparisons **24**
5.1 Demographics 25
5.2 Latent measurement modelling 28
5.3 Diet 29

5.3.1 Main effects of covariates on diet 33
5.3.2 Interactions Covariates x Class 34
5.4 Blood markers 35
5.4.1 Group comparisons on acute infection (CRP>10mg/L) at blood sampling 37
5.4.2 Group comparisons on blood biomarkers 39
5.4.3 Tables at 17 weeks 41
5.4.4 Tables at 27 weeks 42
5.4.5 Interaction class x covariates 45
5.4.6 Sensitivity Analysis 46
5.4.7 Tables at 34 weeks 47
5.5 Group comparison on reports of infections prior to each visit 48
5.6 Group comparisons on gestational weight gain 48
5.7 Pregnancy outcomes 49
5.8 Birth outcomes 50

References **51**

1 Participants exclusion and inclusion in UPBEAT

The UPBEAT RCT[1] aimed to prevent GDM and lower the incidence of large-for-gestational age newborns (>90th customized birthweight centile) by promoting diet substitutions to decrease saturated fat intake and glycaemic load and increase incrementally their physical activity. 1554 women of BMI $\geq$ 30 kg/m^2, age >16 years, carrying singleton pregnancies, void of hypertension, renal disease, pre-gestational diabetes, sick cell disease, thalassemia, coeliac disease, renal disease, systemic lupus erythematous, antiphospholipid syndrome, thyroid disease, current psychosis or under current metformin medication were consented to participate from 8 UK NHS trusts (Bradford, Glasgow, 3 London Centres, Manchester, Newcastle and Sunderland).[1] The trial was designed to randomize women between 15^{+0}-18^{+6} weeks gestation (intervention start), invite them for the oral glucose tolerance test (OGTT) at 27^{+0}-28^{+6} weeks (intervention end) and a last visit between 34^{+0}-36^{+0} weeks of gestation. Randomization was minimized by ethnicity, BMI groups (obesity class I:30.0–34.9, II:35.0–39.9, III: $\geq$ 40 kg/m²), age (<24, 25–29, 30–34, $\geq$ 35 years) and centre.

From 1554 in the original trial, 24 experienced fetal loss or miscarriages, 4 terminated, 5 neonatal deaths occurred (within 28 days of birth), 14 withdrew consent to use data or were lost to follow-up and 17 had unconfirmed fetal/neonatal outcomes. Thus 1490 pregnancies resulted in known live births. Of those, 1369 women who had at least one completed EPDS questionnaire were included for the purpose of studying depressive symptom trajectories in pregnancy and their outcomes, see Figure S1. Women who were missing all EPDS scores and were excluded did not differ on entry demographics from those included but rather differed by trial site (Table S1.)

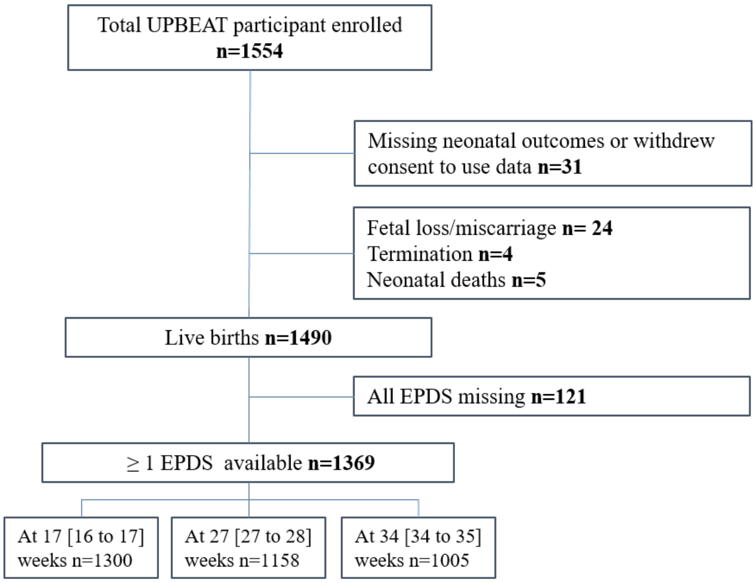

Figure S1: Consort diagram of participants. 693 (50.62%) women were randomized to the intervention and 676 (49.38%) to standard care.

Table S1: Comparison between excluded and included participants who answered to at least one EPDS questionnaire (total n=1490).

	Excluded	Included	p
n	121	1,369	
BMI (median [IQR])	35.40 [32.70, 39.10]	35.00 [32.80, 38.50]	0.538
Age(years) (mean (SD))	30.50 (5.68)	30.48 (5.47)	0.968
Main ethnicity (%)			0.397
White	79 (65.3)	857 (62.6)	
Black	31 (25.6)	351 (25.6)	
Asian	3 (2.5)	85 (6.2)	
Other	8 (6.6)	76 (5.6)	
IMD (%)			0.461
Least Deprived	5 (4.2)	54 (4.0)	
2nd quintile	8 (6.7)	90 (6.6)	
3rd quintile	11 (9.2)	155 (11.4)	
4th quintile	50 (42.0)	462 (33.8)	
Most deprived	45 (37.8)	604 (44.2)	
Income (%)			0.275
< £12,688	27 (22.3)	247 (18.0)	
£12,688 - £17,628	7 (5.8)	159 (11.6)	
£17,629 - £23,452	14 (11.6)	112 (8.2)	
£23,453 - £32,500	13 (10.7)	169 (12.3)	
> £32,500	41 (33.9)	479 (35.0)	
Prefers not to answer	19 (15.7)	203 (14.8)	
Born in the UK (%)	81 (66.9)	922 (67.3)	1.000
Nulliparous (%)	52 (43.0)	598 (43.7)	0.956
Centre (%)			<0.001
St Thomas'	41 (33.9)	327 (23.9)	
King's College Hospital	29 (24.0)	240 (17.5)	
Newcastle	24 (19.8)	206 (15.0)	
Glasgow	25 (20.7)	234 (17.1)	
Manchester	1 (0.8)	134 (9.8)	
Bradford	0 (0.0)	50 (3.7)	
Sunderland	0 (0.0)	82 (6.0)	
St Georges'	1 (0.8)	96 (7.0)	

Note:
From 1554 women enrolled, 1490 pregnancies resulted in confirmed live births, 1369 provided at least one EPDS questionnaire and were included in this study. Normally and non-normally distributed variables are compared with a t-test and a Kruskal-Wallis test, respectively. Categorical variables were analysed with a Chi-squared test. IMD: Index of Multiple Deprivation; BMI: Body Mass Index.

2 Variables

2.1 Edinburgh Postnatal Depression Scale (EPDS)

Ten items within the EPDS ask about experiences and feelings occurring seven days previously, rated on a four-point scale according to frequency (e.g. Item 9: “I have been so unhappy that I have been crying”; “Yes, most of the time”=3, “No, Never”=0). A frequency matrix is provided in Table S2. Items three and five to 10 are reverse coded and the highest possible score is 30. We report on Item 10 (self-harm) in Table S3 as a high score triggers referral pathway to antenatal mental health service regardless of the total EPDS score.

Table S2: Frequency matrix of available EPDS.

	Time point 1	Time point 2	Time point 3
Time point 1	1300		
Time point 2	1116	1158	
Time point 3	939	974	1005

Note:
The responses for the EPDS were provided by 1300 women at median[IQR] 17[16,17] weeks GA, 1156 women at 27[27,28] weeks and 1004 women at 34[34,35] weeks. The count(%) of women who responded above the 13-point threshold was 157/1300 (12.1%), 118/1158(10.2%) and 82/1005(8.2%) at the 1st,2nd and 3rd time points respectively.

Table S3: Participant response on item 10 (’self-harm’) of the EPDS at median 17, 27 and 34 weeks gestation

	Time 1	Time 2	Time 3
Never	1256	1116	978
Hardly	25	31	20
Sometimes	18	8	7
Quite often	1	3	0
Missing	*69*	*211*	*364*

Note:
From 1369 women who responded to at least one questionnaire.

2.2 Demographics/Baseline variables

In analyses of outcomes, due to low frequency, ‘other’ and ‘temporary’ accommodation categories were aggregated. Only 5/1369 women had more than one child under 2 years of age in the household, the variable was dichotomised into none vs one or more. ‘Other’ employment included: Doing something else/Retired/Unable to work, for details see Table 1 of main manuscript. The standard error of the “Other” accommodation for the Depressed group could not be calculated due to zero probability. In regression analyses, ethnicity as a covariate was dichotomised into white/non-white for modelling simplification and convergence.

2.3 Anthropometrics

Sum of skinfold thickness measurements (mean triceps + mean biceps + mean subscapular + mean suprailiac taken in triplicate) were obtained with Harpenden skinfold callipers to the nearest millimetre. Waist, hip and thigh circumferences were measured to the nearest centimetre and the neck to the nearest millimetre with a plastic tapemeasure.

Table S4: Entry characteristics at first visit in the whole sample of 1369 obese pregnant women.

Factor		N=1369
**Anthropometrics**	BMI	35.0 (32.8, 38.5)
	Neck (cm)	36.6(2.5)
	Waist (cm)	106(100,113)
	Hip (cm)	121(116,128)
	Thigh (cm)	68.5 (6.6)

Note:
Continuous variables presented as mean(standard deviation) or median (interquartile range) if non-normally distributed. Missing: neck/hip/waist/thigh measurement=10.

2.4 Diet

In Table S5 we present available data on Total energy (kcal), Glycaemic load (per 100g; calculated for each food as the glycaemic index x carbohydrates amount/100), saturated fat (g) and dietary composition for carbohydrates, saturated fat, protein, sugar as a % of total energy.

Table S5: Dietary outcomes available from a total of 1369 participants.

	17 weeks	27 weeks
Energy (kcal)	997 (72.8%)	858 (62.7%)
Glycaemic load/100g	997 (72.8%)	858 (62.7%)
Saturated fat (gr)	997 (72.8%)	858 (62.7%)
Total Fat (% Energy)	997 (72.8%)	858 (62.7%)
Carbohydrates (% Energy)	997 (72.8%)	858 (62.7%)
Saturated fat (% Energy)	997 (72.8%)	858 (62.7%)
Protein (% Energy)	997 (72.8%)	858 (62.7%)
Sugar (% Energy)	997 (72.8%)	858 (62.7%)

Note:
Participant dietary intake was included if total energy count was within 1076 to 4780 kcal range to control for over- and under-reporting.

2.5 Blood Markers collection, availability and processing

The first and last blood samples were random samples and the second was obtained after an overnight fast (as per OGTT protocol). All samples were kept at -80 degrees after processing (within 2 hours of collection). The count of blood samples at each time point is provided in table S7 and the laboratory methods in table S6. All the markers obtained by conventional biochemical assays were processed by blinded techniciansat the University of Glasgow following manufacturer's calibrators and quality controls apart from human placental lactogen which was obtained by DiabetOmics, Inc, Beaverton, Oregan, USA. Glucose levels at the OGTT was processed at each trial centre to obtain GDM status by the International Association of Diabetes and Pregnancy Study Groups criteria (IADPSG, see below). Amino acids, fatty acids and glycoprotein acetyles were measure by targeted NMR metabolomics platform (Nightingale Health, Finland) with no batch effect as described previously.[2]

Table S6: Properties of blood biochemical analyses.

Group	Biomarker	Units	Sample type	Method	Platform	CV(%)
**Conventional Biochemical Platforms**						
Glycaemic Markers	Insulin	mU/l	plasma	Electrochemiluminescence immunoassay	Roche, Cobas e411	< 10.3
Glycaemic Markers	HbA1c	mmol/mol	whole blood	Turbidimetric inhibition immunoassay	Roche, Cobas c311	< 1.4
Glycaemic Markers	HbA1c	% (old units)	whole blood	Turbidimetric inhibition immunoassay	Roche, Cobas c312	< 1.5
Glycaemic Markers	C-peptide	ng/ml	serum	Electrochemiluminescence immunoassay	Roche, Cobas e411	< 6.2
Glycaemic Markers	glucose	mmol/l	plasma	Enzymatic hexokinase	Roche Cobas c311	< 2.4
Metabolic Markers	Cholesterol	mmol/l	plasma	Enzymatic, colorimetric	Roche Cobas c311	< 2.4
Metabolic Markers	Triglycerides	mmol/l	plasma	Enzymatic, colorimetric	Roche Cobas c311	< 3.6
Metabolic Markers	HDL	mmol/l	plasma	Homogeneous enzymatic, colorimetric	Roche Cobas c311	< 4.5
Metabolic Markers	LDL	mmol/l	plasma	Homogeneous enzymatic, colorimetric	Roche Cobas c311	< 3.3
Adipokines	Adiponectin	ug/ml	plasma	Enzyme-linked immunosorbent assay	R and D Systems	< 6.9
Adipokines	Leptin	pg/ml	plasma	Enzyme-linked immunosorbent assay	R and D Systems	< 2.0
Inflammation	hs-IL-6	pg/ml	plasma	Enzyme-linked immunosorbent assay	R and D Systems	< 9.8
Inflammation	hs-CRP	mg/L	plasma	Particle enhanced immunoturbidimetric	Roche, Cobas c311	< 7.1
Endothelial marker	t-PA antigen	ng/ml	plasma	Enzyme-linked immunosorbent assay	Asserchrom (Stago)	< 5.7
Placenta	Human placental lactogen	ng/ml	serum	Enzyme-linked immunoassay	R and D Systems	< 5.0
Placenta	Placental growth factor	pg/ml	Plasma	Fluorescence Immunoassay	Alere, Triage Meter Pro	
Vitamin	Vitamin D	ng/ml	serum	Electrochemiluminescence immunoassay	Roche, Cobas e411	< 11.2
**NMR metabolomics platform**						
Amino acids	Alanine	mmol/l	serum or EDTA plasma	NMR	Nightingal Health, Finland	
Amino acids	Glutamine	mmol/l	serum or EDTA plasma	NMR	Nightingal Health, Finland	
Amino acids	Glycine	mmol/l	serum or EDTA plasma	NMR	Nightingal Health, Finland	
Amino acids	Histadine	mmol/l	serum or EDTA plasma	NMR	Nightingal Health, Finland	
Amino acids (Branched-chain)	Isoleucine	mmol/l	serum or EDTA plasma	NMR	Nightingal Health, Finland	
Amino acids (Branched-chain)	Leucine	mmol/l	serum or EDTA plasma	NMR	Nightingal Health, Finland	
Amino acids (Branched-chain)	Valine	mmol/l	serum or EDTA plasma	NMR	Nightingal Health, Finland	
Amino acids (Aromatic)	Phenylalanine	mmol/l	serum or EDTA plasma	NMR	Nightingal Health, Finland	
Amino acids (Aromatic)	Tyrosine	mmol/l	serum or EDTA plasma	NMR	Nightingal Health, Finland	
Fatty Acids	Total fatty acids	mmol/l	serum or EDTA plasma	NMR	Nightingal Health, Finland	
Fatty Acids	estimated degree of unsaturation		serum or EDTA plasma	NMR	Nightingal Health, Finland	
Fatty Acids	Omega-3	mmol/l	serum or EDTA plasma	NMR	Nightingal Health, Finland	
Fatty Acids	Omega-6	mmol/l	serum or EDTA plasma	NMR	Nightingal Health, Finland	
Fatty Acids	Polyunsaturated fatty acids	mmol/l	serum or EDTA plasma	NMR	Nightingal Health, Finland	
Fatty Acids	Monounsaturated fatty acids 16:1;18:1	mmol/l	serum or EDTA plasma	NMR	Nightingal Health, Finland	
Fatty Acids	Saturated fatty acids	mmol/l	serum or EDTA plasma	NMR	Nightingal Health, Finland	
Fatty Acids	Docosahexaenoic acid (DHA) 22:6	mmol/l	serum or EDTA plasma	NMR	Nightingal Health, Finland	
Fatty Acids	Linoleic acid 18:2	mmol/l	serum or EDTA plasma	NMR	Nightingal Health, Finland	
Inflammation	Glycoprotein acetyls (a1-acid glycoprotein)	mmol/l	serum or EDTA plasma	NMR	Nightingal Health, Finland	

Note:
The Coefficient of Variation (CV) based on the highest value at time 1 or 2. Glucose at time point 2 issued by centre as per OGTT protocol.

Table S7: Biomarkers available from a total of 1369 participants.

Group	Biomarker	Time point 1	Time point 2	Time point 3
Adipokines	adiponectin	967 (70.6%)	898 (65.6%)	733 (53.5%)
Adipokines	leptin	967 (70.6%)	897 (65.5%)	
Amino Acids	alanine	945 (69.0%)	887 (64.8%)	715 (52.2%)
Amino Acids	glutamine	943 (68.9%)	886 (64.7%)	714 (52.2%)
Amino Acids	glycine	945 (69.0%)	887 (64.8%)	715 (52.2%)
Amino Acids	histidine	944 (69.0%)	886 (64.7%)	715 (52.2%)
Amino Acids	isoleucine	945 (69.0%)	887 (64.8%)	715 (52.2%)
Amino Acids	leucine	945 (69.0%)	887 (64.8%)	715 (52.2%)
Amino Acids	phenylalanine	945 (69.0%)	887 (64.8%)	715 (52.2%)
Amino Acids	tyrosine	944 (69.0%)	886 (64.7%)	715 (52.2%)
Amino Acids	valine	945 (69.0%)	887 (64.8%)	715 (52.2%)
Fatty Acids	Degree of unsaturation	945 (69.0%)	886 (64.7%)	716 (52.3%)
Fatty Acids	DHA	945 (69.0%)	886 (64.7%)	716 (52.3%)
Fatty Acids	Linoleic Acid	945 (69.0%)	886 (64.7%)	716 (52.3%)
Fatty Acids	Monounsaturated	945 (69.0%)	886 (64.7%)	716 (52.3%)
Fatty Acids	Omega-3	945 (69.0%)	886 (64.7%)	716 (52.3%)
Fatty Acids	Omega-6	945 (69.0%)	886 (64.7%)	716 (52.3%)
Fatty Acids	Polyunsaturated	945 (69.0%)	886 (64.7%)	716 (52.3%)
Fatty Acids	Saturated	944 (69.0%)	886 (64.7%)	716 (52.3%)
Fatty Acids	Total FA	945 (69.0%)	886 (64.7%)	716 (52.3%)
Fatty Acids %	DHA of total FA	945 (69.0%)	886 (64.7%)	716 (52.3%)
Fatty Acids %	Linoleic Acid of total FA	945 (69.0%)	886 (64.7%)	716 (52.3%)
Fatty Acids %	Monounsaturated of total FA	945 (69.0%)	886 (64.7%)	716 (52.3%)
Fatty Acids %	Omega-3 of total FA	945 (69.0%)	886 (64.7%)	716 (52.3%)
Fatty Acids %	Omega-6 of total FA	945 (69.0%)	886 (64.7%)	716 (52.3%)
Fatty Acids %	Polyunsaturated of total FA	945 (69.0%)	886 (64.7%)	716 (52.3%)
Fatty Acids %	Saturated of total FA	944 (69.0%)	886 (64.7%)	716 (52.3%)
Glycaemic markers	c-peptide	959 (70.1%)	890 (65.0%)	728 (53.2%)
Glycaemic markers	glucose	959 (70.1%)		733 (53.5%)
Glycaemic markers	hba1c	905 (66.1%)		
Glycaemic markers	HOMA-2IR		878 (64.1%)	
Glycaemic markers	insulin	972 (71.0%)	898 (65.6%)	704 (51.4%)
Inflammation and endothelial function	CRP	969 (70.8%)	894 (65.3%)	733 (53.5%)
Inflammation and endothelial function	Glycoprotein acetyls	945 (69.0%)	887 (64.8%)	715 (52.2%)
Inflammation and endothelial function	IL-6	968 (70.7%)	895 (65.4%)	
Inflammation and endothelial function	tPA-antigen	968 (70.7%)	898 (65.6%)	
Metabolic	cholesterol	972 (71.0%)	896 (65.4%)	733 (53.5%)
Metabolic	HDL	969 (70.8%)	896 (65.4%)	733 (53.5%)
Metabolic	LDL	969 (70.8%)	896 (65.4%)	733 (53.5%)
Metabolic	triglycerides	969 (70.8%)	891 (65.1%)	733 (53.5%)
Other	HPL	944 (69.0%)		
Other	Plgf	970 (70.9%)	897 (65.5%)	
Other	Vit-D	953 (69.6%)		

Note:
FA: Fatty Acids, DHA: Docosahexaenoic acid, tPA-antigen: Tissue plasminogen activator antigen, IL-6: Interleukin-6, CRP: C-reactive protein, LDL: low-density lipoproteins, HDL: high-density lipoproteins, HPL: Human Placental Lactogen, Plgf: Placental growth factor.

2.6 Pregnancy complications

2.6.1 Obstetric

Obstetric diagnoses were those which received clinical diagnoses during pregnancy (GDM) or after revision of the pregnancy outcomes from the electronic records and pregnancy notes after birth (preeclampsia or gestational hypertension[GHT]). Due to the low frequency in GHT and the established adverse effect of PE in the literature only PE and GDM are included in group comparisons of outcomes but all comorbidity patterns are reported below for clarity.

GDM was diagnosed according to the International Association of Diabetes and Pregnancy Study Groups (IADPSG) criteria, from 27 to 28^{+6} weeks of gestation at OGTT visit, if fasting glucose $\geq$ 5.1 mmol/L and/or if 1-hour glucose $\geq$ 10mm/L and/or 2-hour glucose $\geq$ 8.5 mmol/L. Women who received GDM diagnosis were referred to standard antenatal care based on the NICE guidelines.

Preeclampsia was diagnosed following the International Society for the Study of Hypertension in Pregnancy (ISSHP;)[3] criteria as: The presence of proteinuria (spot urine protein/creatinine $\geq$ 30mg/mmol [0.3mg/mg] or $\geq$ 300 mg/day or minimum 1g/L ["2 +"] on dipstick testing) with two measures of systolic ($\geq$ 140mmHg) or diastolic blood pressure ($\geq$ 90 mmHg) taken four hours apart. Preeclampsia was recorded by the research team after revision of the recorded blood pressure and proteinuria values.[4]

Gestational hypertension was recorded as two measures of systolic ($\geq$ 140mmHg) or diastolic blood pressure ($\geq$ 90 mmHg) taken four hours apart in the absence of proteinuria.

Table S8: Obstetric comorbidities in n=1369 participants.

n=1369	GDM	PE	GHT
**All**			
No	882 (64.4%)	1271 (92.8%)	1292 (94.4%)
Yes	303 (22.1%)	82 (6.0%)	56 (4.1%)
missing	184 (13.4%)	16 (1.2%)	21 (1.5%)

Note:
GDM diagnosed according to the International Association of Diabetes and Pregnancy Study Groups (IADPSG) criteria, missing due to no-show at the Oral Glucose Tolerance Test. PE and GHT marked at revision of all recorded blood pressure measurement and test of proteinuria. GDM: Gestational Diabetes Mellitus, PE:Pre-eclampsia, GHT: Gestational Hypertension.

Table S9: Obstetric comorbidity patterns

n=1369	GDM	PE	GHT	Met criteria	n(%)	Missing N(%)
	X	X		No	1143 (83.5%)	195 (14.2%)
	X	X		Yes	31 (2.3%)	
	X		X	No	1157 (84.6%)	198 (14.4%)
	X		X	Yes	14 (1.0%)	
	X			No	927 (67.7%)	188 (13.7%)
	X			Yes	254 (18.6%)	
		X		No	1130 (82.5%)	193 (14.1%)
		X		Yes	46 (3.4%)	
			X	No	1143 (83.5%)	194 (14.2%)
			X	Yes	32 (2.3%)	
All absent					789 (57.6%)	184 (13.4%)
One or more					396 (28.9%)	

Note:
Missing value counted if any obstetric diagnosis had missing diagnosis for each pattern. PE and GHT diagnoses are mutually exclusive. GDM: Gestational Diabetes Mellitus, PE:Pre-eclampsia, GHT: Gestational Hypertension.

2.6.2 Infections

Data on maternal infection during pregnancy was collected by research midwives at each visit as part of the UPBEAT study for the period prior to each visit and specified for respiratory infection/flu, lower urinary tract infection (UTI), pyelonephritis, gastroenteritis, vaginal candida (VC), suspected vaginal candida and “other infections”, which were coded Yes/No and specified if of other type. For positive responses, participants self-reported if they had “used antibiotic, antifungal or antiviral treatment” (coded Yes/No) or “other treatment” (specified in free text). See Table S10

Respiratory infection/flu was defined at cold-like symptoms with/without cough, or febrile illness with muscle pains and lethargy, including sinusitis and tonsilitis.

UTI was confirmed UTI on culture without pyrexia and renal angle tenderness.

Pyelonephritis was confirmed UTI with pyrexia (>38 degrees C) and/or renal angle tenderness.

Gastroenteritis was positive if the woman experienced repeated diarrhoea with/without vomiting.

VC was proven candida on culture of vaginal swab. *Suspected VC* was defined as symptoms of candida without culture but woman self medicated.

Other infection was specified and included herpes or group B strep on vaginal swab.

There were 361, 317 and 234 women who reported at least one infection during the period prior to the first, second and third visit respectively (Table S10). Of these 68.7%, 73.2% and 67.9% had reported having taken antifungal, antiviral or antibiotic treatment (Table S11).

2.6.3 Other

Hospital admission: data was collected from summary notes or medical records for any admission to hospital during pregnancy. The indications were available but not used as outcomes in this study, see Table S12.

Table S10: Frequency of infections in 1369 pregnancies at three visits.

n=1369	Time point 1	Time point 2	Time point 3	n(%)
**All**				
No	986(72.0%)	866(63.2%)	773(56.5%)	
Yes	358(26.2%)	313(22.9%)	234(17.1%)	
Total	1344	1179	1007	
Missing	25(1.8%)	190(13.9%)	362(26.4%)	
**Missing data pattern**				
				980(71.59%)
			X	181(13.22%)
		X		9(0.66%)
		X	X	174(12.71%)
	X			16(1.17%)
	X		X	2(0.15%)
	X	X		2(0.15%)
	X	X	X	5(0.37%)
**Infection in complete cases (n=980)**				
	Yes	Yes	Yes	51(5.20%)
	No	Yes	Yes	49(5.00%)
	Yes	No	Yes	31(3.16%)
	No	No	Yes	97(9.90%)
	Yes	Yes	No	54(5.51%)
	No	Yes	No	117(11.93%)
	Yes	No	No	116(11.83%)
	No	No	No	465(47.45%)

Note:
Women were asked about whether they suffered from at least one infection in the period prior to the visit, i.e. before 17 weeks, between 1 and 27 weeks, and between 27 and 34 weeks. The frequency of any infection for participants who responded at all 3 time points (n=980): never = 465(47.4%), one visit = 330(33.6%), two visits = 134(13.6%) and all three visits = 51(5.2%).'X' refers to missing data.

Table S11: Infections in 1369 pregnancies

Infections	Time point 1<br>n(%)	Time point 2<br>n(%)	Time point 3<br>n(%)
Any	361 (26.4%)	317 (23.2%)	234 (17.1%)
None	983 (71.8%)	862 (63.0%)	773 (56.5%)
**Type**			
Flu/Respiratory tract	81 (5.9%)	71 (5.2%)	64 (4.7%)
Lower UTI	147 (10.7%)	95 (6.9%)	55 (4.0%)
Pyelonephritis	1 (0.1%)	2 (0.1%)	2 (0.1%)
Gastroenteritis	12 (0.9%)	20 (1.5%)	6 (0.4%)
Vaginal candida	52 (3.8%)	63 (4.6%)	61 (4.5%)
Suspected Vag.Cand	24 (1.8%)	21 (1.5%)	17 (1.2%)
Other infection	78 (5.7%)	78 (5.7%)	49 (3.6%)
**Treatment**			
None	103 (28.5%)	81 (25.6%)	74 (31.6%)
Yes	248 (68.7%)	232 (73.2%)	159 (67.9%)

Note:
Women were asked about whether they experienced any infection in the period prior to each visit. Women could answer positive for more than one type. There were 1344 responses at Time point 1, 1179 at time point 2 and 1007 at time point 3. Percentages in the table are of the total sample (1369).

Table S12: Admissions among n=1369 pregnancies.

Outcome		n=1369	Missing
Antenatal Maternal admission	No	1240 (90.6%)	
	Yes	129 (9.4%)	

2.7 Birth and neonatal outcomes

At the time of birth 474 women (34.6%) had been induced, 716(52.3%) gave birth via vaginal delivery and 490(35.8%) had a Cesarean section (CS) where 132 were indicated for maternal/fetal compromise but not life threatening (category 2 CS) and 96 for immediate threat to life (category 1 CS). Preterm birth (prior to 37 weeks) occurred in 81 pregnancies (5.9%), 102 (7.5%) infants were admitted to the NICU of which 46 stayed for 4 days or longer. Overall 1033 neonates stayed 1-3 days (75.5%) in hospital. We provide further information on the birth outcomes for the preterm births in Table S14 in order to dissociate spontaneous labour and premature rupture of membrane from induced pre-term births.

The following variables were included as birth outcomes :

Gestational age at birth which was calculated in postmenstrual days and a new variable created where GA birth was dichotomised as $\leq$ 34 week, $\leq$ 37 weeks to estimate rate of early and late preterm birth respectively but only preterm <37 weeks is included as an outcome in our comparative analyses. Spontaneous/Premature Rupture of Membranes (PROM) are also included in analyses to differentiate against indicated premature birth given the physiological aetiologies of spontaneous birth and clinical context of the event. Further details on preterm deliveries and the labour onset are included for clinical interest.

Birth weight centile by WHO: centile calculated according to WHO is adjusted for sex and gestational age and is included as an outcome for comparative value. Large-for-gestational age (LGA) was defined as $\geq 90^{th}$ centile and small-for-gestational age (SGA) as $< 10^{th}$ centile.
C-section (vs no CS), *induction of labour* (IOL), and *blood loss*, were retrieved from medical records/pregnancy notes. *NICU admission* was reviewed from discharge summaries or from UK NICU database (www.neonatal.net) and included admission from birth or from the postnatal ward and included in comparative analyses. The clinical indication recorded (may be more than 1) for SGA, respiratory distress, birth asphyxia, infection, congenital abnormality, feeding problem, phototherapy, hypoglycemia, cyanosis, drug withdrawal or other indications are included for clinical interest but are not outcomes used in analyses.

Other outcomes reported but not included in analyses:
Mode of delivery and *Caesarean-section indication Birthweight* and *sex*, *NICU indication and length*, *Apgar score* and *Neonatal length of stay* were all collected from the discharge summaries.
Birth weight centile: a customised centile using the GROW calculator, adjusted for maternal weight, height, ethnicity, parity, birthweight, sex and gestational age at delivery. Large-for-gestational age (LGA) was defined as $\geq 90^{th}$ centile and small-for-gestational age (SGA) as $< 10^{th}$ centile.

Table S13: Birth and infant outcomes in n=1369 pregnancies.

Outcome		n=1369	Missing
**Birth**			
Labour onset	Spontaneous	602(44.0%)	
	Induction	474(34.6%)	
	Prelabour CS	224(16.4%)	
	PROM+augmentation	66(4.8%)	3(0.2%)
Induction of labour	No induction performed	892 (65.2%)	3 (0.2%)
	Induction performed	474 (34.6%)	
Mode of Delivery	LSCS in labour	227 (16.6%)	3 (0.2%)
	Operative vaginal	160 (11.7%)	
	Prelabour CS	263 (19.2%)	
	Unassisted vaginal	716 (52.3%)	
	CS not applicable	875 (63.9%)	5 (0.4%)
	Delivery timed to suit the woman and staff	169 (12.3%)	
	Immediate threat to life of the woman or fetus	96 (7.0%)	

Table S13: Birth and infant outcomes in n=1369 pregnancies. *(continued)*

Outcome		n=1369	Missing
CS indication	Maternal/fetal compromise which is not immediately life threatening	132 (9.6%)	
	No maternal or fetal compromise but needs early delivery	92 (6.7%)	
Blood loss	Blood loss <1000mls	1165 (85.1%)	22 (1.6%)
	Blood loss >=1000mls	182 (13.3%)	
**Infant**			
Sex	Male	695 (50.8%)	3 (0.2%)
	Female	671 (49.0%)	
Gestation age at birth	Days	279 [271, 286]	3 (0.2%)
	Weeks	39.9[38.7,40.9]	
Preterm birth (PTB)	Delivery >34 weeks	1342 (98.0%)	3 (0.2%)
	Delivery <34 weeks	24 (1.8%)	
	Delivered >37 weeks	1285 (93.9%)	
	Delivered < 37 weeks	81 (5.9%)	
Spontaneous PTB or PROM		56(4.1%)	3(0.2%)
Birthweight (g)		3447[3120,3788]	3 (0.2%)
Low birthweight <1.5kg		12(0.88%)	3 (0.2%)
SGA 10% WHO		83(6.06%)	3 (0.2%)
SGA 10% customised		150(10.96%)	3 (0.2%)
LGA 90% WHO		158(11.54%)	3 (0.2%)
LGA 90% customised		111(8.11%)	3 (0.2%)
Apgar at 5 min	median[IQR]	10[9,10]	26(1.9%)
	>=7	1324(98.6%)	
	<7	19(1.4%)	
NICU	Not admitted	1264 (92.3%)	3 (0.2%)
	Admitted	102 (7.5%)	
NICU Indication	Preterm	32 (2.3%)	2/102(2.0%)
	SGA	3 (0.2%)	
	Respiratory distress	41 (3.0%)	
	Birth asphyxia	3 (0.2%)	
	Infection	22 (1.6%)	
	Congenital abnormality	3 (0.2%)	
	Feeding problem	2 (0.1%)	
	Phototherapy	9 (0.7%)	
	Hypoglycemia	24 (1.8%)	
	Cyanosis	2 (0.1%)	
	Other NICU indication	29 (2.1%)	
NICU Days admitted	median[IQR]	3[1,14]	8/102(7.8%)
	1 day	29 (2.1%)	
	2-3 days	19 (1.4%)	

Table S13: Birth and infant outcomes in n=1369 pregnancies. *(continued)*

Outcome		n=1369	Missing
NICU Days admitted	4-13 days	22 (1.6%)	
	14+ days	24 (1.8%)	
Neonatal nights	0 nights	111 (8.1%)	22 (1.6%)
	1-3nights	1033 (75.5%)	
	4-10 nights	174 (12.7%)	
	More than 10 nights	29 (2.1%)	

Note:
102 neonates were admitted to the NICU after revision of discharge summaries and search on UK database (www.neonatal.net) among whom two had missing NICU indication (one born at 28+6 weeks, weighing 1130gr with unknown Apgar score and one born at 38+1 weeks weighing 3890g with a 5-min Apgar score of 6). LGA: Large for gestational age; NICU: Neonatal Intensive Care Unit; SGA: Small for gestational age.

Table S14: Labour and birth outcomes in 81 pre-term deliveries (<37 weeks gestation).

	n	%
**Labour Onset**		
Spontaneous	41	50.6
Induction	11	13.6
Prelabour CS	14	17.3
PROM+augmentation	15	18.5
**Mode of Delivery**		
CS in labour	8	9.9
Operative vaginal	8	9.9
Prelabour CS	16	19.8
Unassisted vaginal	49	60.5
**CS indication**		
CS not applicable	57	70.4
Delivery timed to suit the woman and staff	2	2.5
Immediate threat to life of the woman or fetus	8	9.9
Maternal/fetal compromise which is not immediately life threatening	9	11.1
No maternal or fetal compromise but needs early delivery	5	6.2

Note:
CS:Caesarean section; PROM: Premature rupture of membranes.

3 Missing data

Three responses were missing out of all the 3463 EPDS questionnaires included in this study. At time point two, one participant had an answer missing for the item six ("overwhelmed") which was inputted to zero as her two other scores for this item were zero. Another participant had a missing answer for item seven ("poor sleep") also at time point two which was inputted to 2 based on the her answer scoring two at the first time point and one at third time point. At time point three one participant did not answer item six which was inputted to zero because she scored zero for this item at the two previous time points. A frequency matrix is provided in table S2 and the responses to the Self-harm item in table S3 for it is the only item which requires direct referral to mental health services regardless of the total score.

Demographic and lifestyle variables collected at 17 weeks gestation were available for all women, expect four (0.29%) had missing data on the index of multiple deprivation, 39 (2.85%) on accommodation and ten (0.73%) on waist/neck/hip and thigh circumferences.

Blood samples were not collected from two centres (Sunderland and King's College Hospital). Logistic regression of missing blood sample at the first visit was associated with centre variable only ($p<0.05$). At time point 2 (OGTT) missing blood sample was associated with younger maternal age and centre. Missing blood at time point 3 was associated with centre, younger maternal age, black ethnicity and intervention arm.

On obstetric complications, 184(13.4%) had missing values, primarily due to no-show at the OGTT so the GDM diagnosis was missing, see Table S9.

There was no infant outcome for 3 pregnancies: One participant withdrew before the OGTT visit (i.e. second) and one participant prior to the third visit who consented for the team to obtain outcome data but these were only partially retrieved. One participant was lost to follow-up before the OGTT for whom there were no infant data (Table S13).

The LCGA and auxiliary group-wise comparisons in adjusted models (of continuous and categorical outcomes) in *Mplus* relied on the Full Information Maximum Likelihood (FIML) function, under the missing at random (MAR) assumption i.e. missingness could be explained by the observed variables. FIML is asymptotically equivalent to multiple imputation and thus helps avoid listwise deletion. This allows computation from all available data and therefore increasing power. The MAR assumption was assumed in this LCGA so modelling the longitudinal trajectories from all time points meant previous EPDS scores could predict missingness on the next score, i.e. based on the reasoning that high levels of depression being associated with attrition or not attending the next visit. Equally, given the known predictors for missingness on other outcomes, values are presented without adjustment and adjusted by age, randomisation (visit 2 and 3 only), ethnicity (white/other) to meet the FIML assumption and latent obesity and latent SES to remove the effects of baseline differences and for theoretical reasons.

4 Latent Class Growth Analaysis - LCGA

We favoured LCGA over Growth Mixture Modelling because we did not expect that within group variation in EPDS scores to be easily interpretable when depressive symptoms are inferred from the total EPDS score which measures feelings over the previous short window of 7-days. LCGA can offer more parsimonious results once an overall pattern of score is established longitudinally, one can more confidently assume that effects of mental health may have been present on a continuum of the score and relative to the trajectory identified. Therefore, variance in the intercepts and slopes within-group was fixed to 0 rather than allowing for within group intercept and slope variance to be estimated (i.e., GMM) across pregnancy. Nevertheless GMM models were generated in accordance to the reporting guidelines, as well as the complete case LCGA (see below). The analysis commands for the LCGA in *Mplus* are included below.

```
DATA: !listwise=ON # uncommented in full case analysis

ANALYSIS:
TYPE = MIXTURE;
starts=700 90;
stiterations=10;
estimator =mlr;
LRTSTARTS= 20 5 200 50;

MODEL:

%overall%
i s | EPDStotal_T1@0 EPDStotal_T2@1 EPDStotal_T3@1.6;
i-s@0; # removed in LGMM modelling
```

The first timepoint was set at 0 as the intercept and the second and third time points set at 1 and 1.6 to reflect the timeline at which the questionnaires were obtained. Only a linear model was tested given the 3 timepoints available. The final choice of 700 repeats and 90 final optimisations was chosen based on previous optimisation steps (so as to facilitate running the model in the loop script in the final stage).

4.1 Criteria

One to five-class solutions were generated as the range often identified in similar studies.[5] We evaluated several indices in order to assess and select the best fitting model: for parsimonious fit we present the Aikaike Information Criterion (AIC,),[6] Bayesian Information Criterion (BIC), sample-size Adjusted BIC (aBIC) where the smallest values is preferred. We also evaluated the entropy (measures the accuracy of group assignment and membership) where entropy =1 reflects perfect classification and 0 is poor and > 0.6 is considered good classification in large sample sizes.[7] Additionally, to select which final model would be carried for further analyses we evaluated class sizes (minimum count and % of total), and a significant Lo-Mendell-Rubin Likekihood Ratio Test (LMR-LRT,)[8] was interpreted as an improvement in fit compared to the n-1 class model. A qualitative judgement was also made, opting to apply the principle of parsimony and placing an importance on ease of interpretability and theoretical justifications in the model selection.[9]

4.2 Model selection and interpretation

Although the 5-class model had the lowest AIC/BIC/aBIC values, the smallest class size was n=32 (2.3%) in the 5-class model which was judged to be insufficient for meaningful and powered group comparisons whereas the smallest count was n=62 (4.5%) in the 4-class model. Additionally, the Lo-Mendell-Rubin adjusted LRT p-value was above 0.05 in the 5-class solution suggesting that it was not a significant improvement to the 4-class model whereas the p-value for the 4-class model was significant at 0.0239 over the 3-class model. Despite the 4-class model having the lowest entropy (0.72) it is still considered of good standard i.e. > 0.6 for further analyses of group comparisons especially in large samples such as this one.[7] Figure 1 in the main manuscript shows the probabilities in the item responses for each of the 4 classes and, for illustrative purposes, the distribution of participant EPDS scores within each of the 4 classes as

assigned to their most probably class membership and the estimated means at each visit. The within class distribution of EPDS score at each time point (between-class variance of growth estimates are equal by default) allows to fully exploit the inherent (and theoretically plausible) gradient of the participant symptoms which also provides statistical advantage in further analyses of comparison between the classes. We did not view the presence of outliers to be problematic, in fact given the short time window the EPDS aims to measure (7 previous days) it is in theory conceivable that some participants may experience fluctuations for many contextual reasons (fatigue, stress, work commitments, illness), hence the utility to have more than one time point to establish average score patterns and trajectories. Additionally, given the large group sizes we did not expect the outliers to have large impact within those groups.

In the 4-class solution selected here, we viewed the mean intercepts and growth estimates to label the groups, see Table S16. The unstandardized mean intercepts and slopes obtained from the 4-class solution were the following: *class 1* (n=219, 16%) had a mean intercept of 11.3 points (i.e. at baseline, standard error [SE]=0.55, p<0.001) and mean slope of -0.34 (SE=0.46, p=0.47) which was labelled as *"Moderate"* and stable. *Class 2* (n=575, 42%) had a mean intercept of 3.4 points (SE=0.22, p<0.001) and a slope of -0.9 points (SE=0.1, p<0.001) which was labelled as *"Not Depressed"* with symptoms improving over pregnancy. *Class 3* (n=513, 37.5%) had a mean intercept of 7.5 points (SE=0.5, p<0.001) and a mean slope of -0.74 (SE=0.20,p<0.001) which was labelled *"Mild"* and showed improvement over pregnancy. Finally *class 4* (n=62,4.5%) had a mean intercept of 16.1 points (SE=0.87, p<0.001) and a mean slope of 0.77 (SE=0.56, p=0.174) which was labelled as *"Severe"* with a chronic/stable feature. Hence we find that among 1369 obese women there are 4 latent groups which are distinguished by their average baseline EPDS score and growth trajectories. Two groups of women ("Not Depressed" and "Mild") form 79.47% of the total sample and show low and moderate symptoms which on average improve from baseline 15-18 weeks until 34-36 weeks. Supplementary Table S16 provides the average probabilities of class membership for the 4-class model which shows that the entropy is mostly contributed by Class 1 (labelled 'Moderate', see below) which share observations with Class 3 ('Mild') and class 3 sharing observation with class 2 'Not Depressed'. This is theoretically acceptable and in line of the notion of participant fluctuation in symptom severity and given the sizes of these two classes we viewed this would have minimal effect in group-wise comparisons. Nevertheless, these fractional membership values are taken into account in all following analyses to provide unbiased estimates. Therefore the 4-class solutions was adopted as the best fitting and interpretable model.

However, to ascertain the robustness of the classification we also assessed whether the variability in gestational age at each visit could have an influence so we also performed the analyses adjusting the EPDS scores for GA at each time point within the LCGA and found there was no significant influence of GA (p=0.481, p=0.253 and p=0.198 at visit 1,2 and 3 respectively) so that the group trajectories and proportions stayed equivalent (4.3% vs 4.5%, 16.5% vs 16%, 36.3% vs 37.4% and 43% vs 42%), with the 4-class solution also yielding the best solution by aforementioned criteria. Furthermore, because the intervention may have influenced the EPDS score at visit 2 and 3, we repeated the 4-class LCGA analysis adjusting for the effect of randomization and we found no significant effect on the second (p=0.300) or the third EPDS score (p=0.860) and no change in the class proportions described in the model above.

Additionally, for completeness and in line with the guidelines,[10] (Supplementary Tables S17 and S19 with Figure S2 are provided for comparisons between 1-4 class solutions when using 935 cases with complete data (EPDS available at three time points) vs all available data LCGA as well as the LCGA vs GMM model solutions using all data.

The robustness of FIML to deal with missing data is reflected in the model solutions generated from 935 participants who provided EPDS scores at all 3 timepoints. There the change in model fit indices is only of a degree of magnitude (S2) while it also agrees to a 4-class model as the most appropriate according to the LMR-LRT (Supplementary Table S17). Figure S3 and table S18 show that the overall distribution of means intercept and mean slopes for the 4 classes is almost identical in the "Not Depressed","Mild" and "Moderate" to the LCGA using all available data. The difference between these two models, for the "Severe" class, the intercept is 1 point lower in the complete case analysis (15.06 vs 16.08) and the slope is positively significant (1.16, p=0.039, vs 0.77 p=0.174 in the full data analysis), indicating a worsening of symptoms among these women.

Table S15: Model fit estimates of LCGA 1-5 class solutions and counts per class (%)

n-class Model	LL	BIC	AIC	aBIC	Entropy	LMR aLRT p-value	Count(%) per class				
							Class 1	Class 2	Class 3	Class 4	Class 5
1	-2282	4601	4575	4585			1369(100)				
2	-1774	3607	3565	3581	0.76	0.00	395(29.8)	975(71.2)			
3	-1589	3257	3199	3222	0.77	0.00	96(7.0)	524(38.3)	749(54.7)		
**4**	**-1531**	**3163**	**3090**	**3119**	**0.72**	**0.02**	**62(4.5)**	**219(16.0)**	**513(37.5)**	**575(42.0)**	
5	-1496	3114	3026	3060	0.74	0.30	32(2.3)	62(4.5)	217(15.9)	488(35.6)	570 (41.6)

Note:
One to five class models were generated by Latent Class Growth Analysis using all available data and compared across model fit indices, entropy and whether a n-class model significantly improves the data fit compared to a (n-1)-class model using the Lo-Mendell-Rubin adjusted Likelihood Ratio Test (LMR-aLRT). AIC: Aikaike Information Criterion; BIC: Bayesian Information Criterion; aBIC: sample-size Adjusted BIC; LL:log likelihood.

Table S16: Class-wise average probabilities, slopes and intercepts for the 4-class model.

Average latent class probabilities for most likely latent class membership (row) by latent class (column)					Intercept			Slope		
Class	1	2	3	4	Estimate	95% CI	p-value	Estimate	95% CI	p-value
1	0.79	0.00	0.16	0.05	11.3	[10.24,12.38]	p<0.001	-0.34	[-1.25,0.57]	0.468
2	0.00	0.88	0.11	0.00	3.4	[2.97,3.82]	p<0.001	-0.90	[-1.10,-0.70]	p<0.001
3	0.09	0.14	0.77	0.00	7.5	[6.46,8.48]	p<0.001	-0.74	[-1.13,-0.35]	p<0.001
4	0.12	0.00	0.00	0.87	16.1	[14.37,17.79]	p<0.001	0.77	[-0.34,1.87]	0.174

Note:
The diagonal in the average latent class probabilities reflect the classification accuracy. Entropy of 0.72 is mostly contributed by Class 1 ('Subclinical') which share observations with Class 3 ('Moderate') and class 3 sharing observation with class 2 'Not Depressed'.In the 4-class model selected, each class intercept represents the mean score at baseline visit and the mean growth estimate in symptom scores is represented by the slope. A significant intercept refers to a value different from 0. A positive and significant slope estimate is interpreted as a worsening in depressive symptoms (as evaluated from the total EPDS score) whereas a negative significant slope is interpreted as an improvement in symptoms across pregnancy.

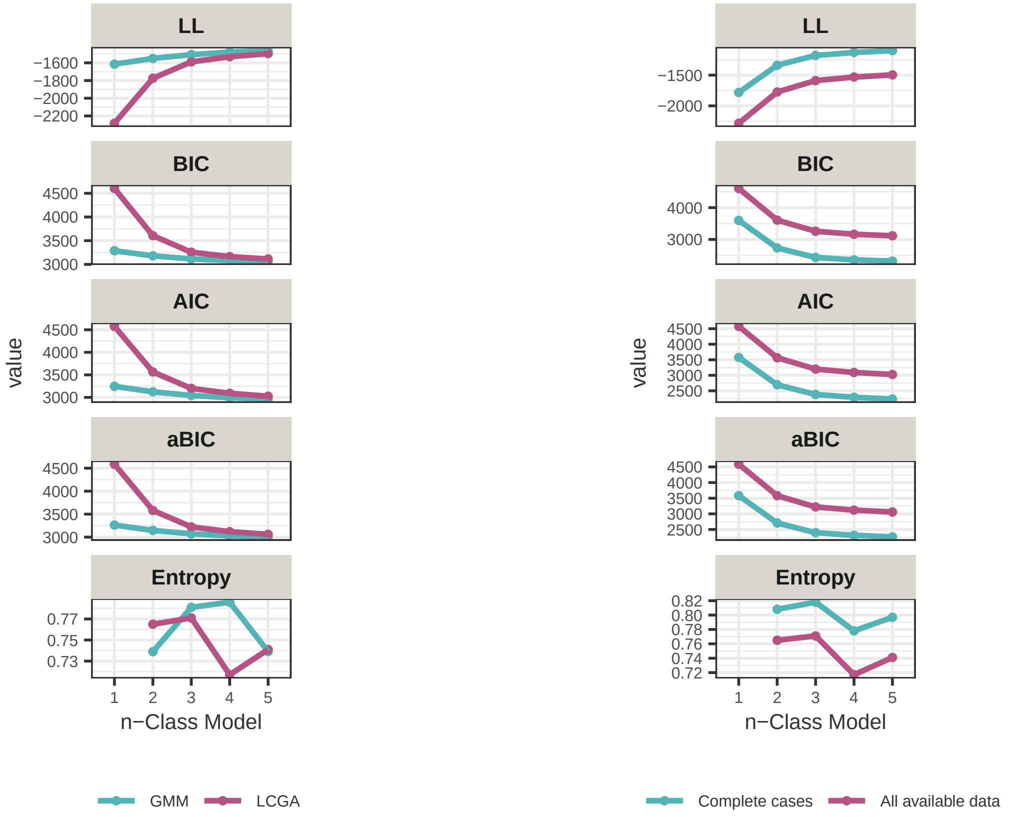

Figure S2: Distribution of Model indices across 1-5 class model solutions using LCGA vs GMM (left panel) with all available data (n=1369) and the 1-5 class model solution by LCGA with all available data (n=1369) vs LCGA with complete cases only (n=935, right panel) . AIC:Aikaike Information Criterion; BIC: Bayesian Information Criterion; aBIC: sample-size Adjusted BIC; LMR-LRT :Lo-Mendell-Rubin Likekihood Ratio Test; LL:log likelihood

4.3 LCGA all data vs complete data

Table S17: Model fit indices for the 1-5 class solutions from subgroup of women with 3 EPDS scores (n=935).

n-class	LL	BIC	AIC	aBIC	Entropy	LMR-LRT p-value
1	-1782	3599	3574	3583		
2	-1339	2733	2695	2708	0.81	0.000
3	-1179	2434	2381	2399	0.82	0.004
**4**	**-1131**	**2359**	**2291**	**2314**	**0.78**	**0.002**
5	-1101	2319	2237	2265	0.80	0.146

Note:
The Latent Class Growth Analysis is generated for the complete cases analysis by listwise deletion. Models representing the best fit according to indices and LMR-LRT are in bold. AIC: Aikaike Information Criterion, BIC: Bayesian Information Criterion, aBIC: sample-size Adjusted BIC; LMR-LRT: Lo-Mendell-Rubin Likelihood Ratio Test; LL: log likelihood.

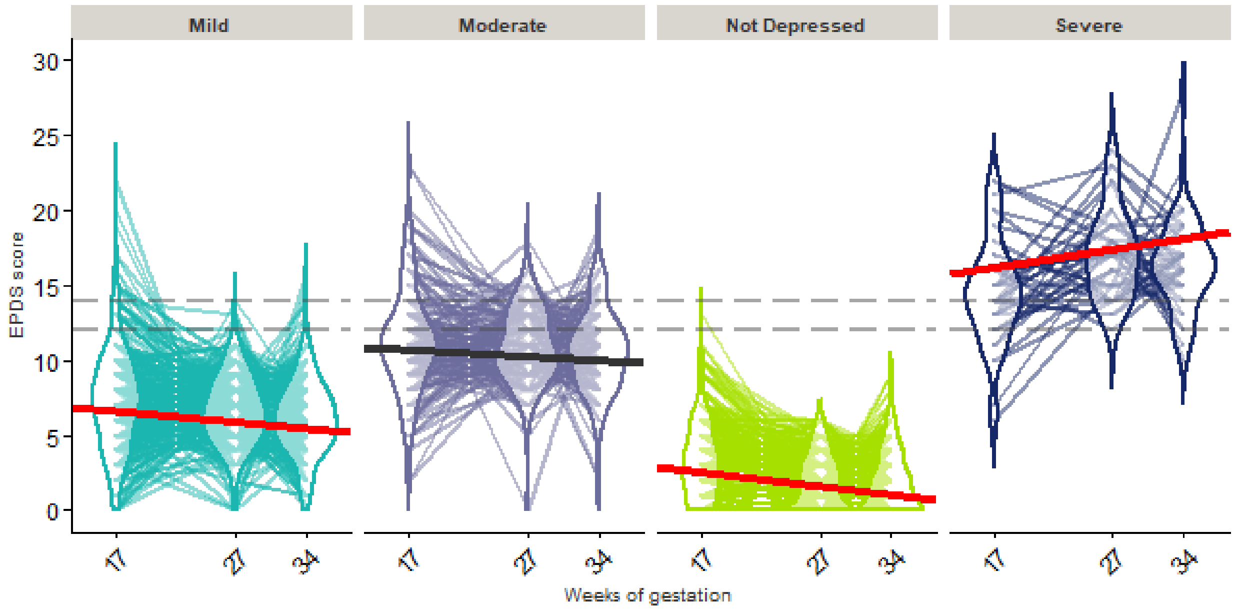

Figure S3: Depressive symptom trajectories and distributions for women who had 3 EPDS scores available (n=935) based on the best fitting solution (4-class model). Each line refers to a subject except bold lines which represent the average trajectory for a class which is red if the slope is significantly different from zero (p<0.05). Violin plots represent the score distribution at each time point.

Table S18: Mean slopes and Intercepts for the 4-class model using cases with complete data.

Class	Intercept	p-value	Slope	p-value
1	3.4	p<0.001	-0.92	p<0.001
2	15.1	p<0.001	1.16	0.039
3	7.3	p<0.001	-0.72	0.001
4	11.1	p<0.001	-0.42	0.347

4.4 LCGA vs GMM

Table S19: Model fit indices using the GMM method.

n-class	LL	BIC	AIC	aBIC	Entropy	LMR-LRT p-value
1	-1615	3287	3246	3262		
2	-1551	3181	3123	3146	0.74	0.00
**3**	**-1507**	**3116**	**3042**	**3071**	**0.78**	**0.00**
4	-1480	3082	2994	3028	0.79	0.21
5	-1462	3069	2964	3005	0.74	0.18

Note:
Models generated from all available data by Growth Mixture Modelling under the Full Information Maximum Likelihood for missing data handling. Model representing the best fit according to indices and LMR-LRT are in bold. AIC: Aikaike Information Criterion; BIC: Bayesian Information Criterion; aBIC: sample-size Adjusted BIC; LMR-LRT: Lo-Mendell-Rubin Likelihood Ratio Test; LL:log likelihood.

5 Group comparisons

Unadjusted analyses are provided by the AUXILIARY option in *Mplus*[7,11] using the "automatic" method. The DCAT function for categorical outcomes and the BCH function for continuous outcomes allowed for group comparison while accounting for the classification error (i.e. fractional class membership) performed during the LCGA, employing a 3-step approach.

The three steps involve 1) building the unconditional Latent Class Growth model 2) assigning individuals to classes based on their posterior class membership probabilities W 3) compares classes on auxiliaries (i.e. class predictors or distal outcomes) accounting for the assignment error in 2). In the second step of the 3-step approach using the BCH method, weights are computed per individual based on their inverse logits error rates and brought into the third step.[12] These methods are thus unbiased by the lack of perfect class assignment[13] (Table S16). Means and categorical probability estimates are robust in large samples with good class assignment (entropy >0.6,).[7] This 3-step approach is viewed as superior and indicated, regardless of entropy, over the "classify-analyse" approach which only compares the classes based on most probable participant class membership.[12] The BCH function has shown to be robust and unbiased even in the assumption of homoskedasticity is in the distal outcome is violated and the variable distribution is non-normal or bimodal rather than normally distributed.[13]

Adjusted analyses are done using the "manual" method[7,14] because the automatic methods described above cannot include covariates. All dietary variables, blood biomarkers, pregnancy and birth outcomes were defined as distal. When distal variables were binary (e.g. SGA) and adjustment included latent covariates (SES and Obesity) the model requires numeric integration and the BCH function is not available. In this case, the 3-step method relies on saving the modal (most likely) class assignment in the first step and their estimated conditional probabilities are computed in the second step to represent the classification error.

As a further approach taken to minimised bias, we implemented an additional theoretically directed application to the structural framework described in this study: *socio-economic status* is, by definition, a multifaceted social construct and we opted to represent this by creating a new latent variable ("SES") using the following variables as indicators: income (ordinal), index of multiple deprivation (ordinal) and highest education attained (ordinal). Similarly, we derived a latent *Obesity* variable using sum of skinfold, waist and hip circumferences and BMI as indicators as a more valid/closer representation of the implied physiological impact of adiposity. A strength in the present study is a large variability of BMI above 30 kg/m^2 which this latent variable can capture in further depth. We verified that the measurement models of each latent variable was valid (i.e. standardized factor loadings were all significant).

Overall, this strategy allows for the reduction in the measurement error, and therefore bias, introduced by otherwise using each indicator as independent proxies of SES or obesity in regression analyses, so their latent forms were used in all adjusted models. To improve model fit further and improve the estimation of the main effects of the covariates, in models including both SES and Obesity, we included their covariance as this is theoretically plausible relationship.

5.1 Demographics

Class-wise baseline values on demographics attributed to the main manuscript are provided in Table S20 and Table S21 and Figure S4.

Table S20: Baseline probabilities and Odds ratio against the Not Depressed class.

		Not Depressed	Severe		Moderate		Mild	
		Probability %	Probability %	OR[95%CI]	Probability %	OR[95%CI]	Probability %	OR[95%CI]
Index Multiple Deprivation (quintile)	Most Deprived	37.10	60.30	ref	51.30	ref	47.10	ref
	4th	36.00	24.00	0.41 [0.19 to 0.87]	33.20	0.67 [0.43 to 1.03]	33.00	0.72 [0.50 to 1.04]
	3rd	14.10	6.10	0.27 [0.07 to 0.96]	8.10	0.41 [0.20 to 0.84]	10.40	0.58 [0.34 to 0.99]
	2nd	7.50	6.50	0.53 [0.10 to 2.77]	6.70	0.64 [0.25 to 1.65]	5.50	0.58 [0.28 to 1.19]
	Least Deprived	5.30	3.10	0.36 [0.07 to 1.99]	0.80	0.10 [0.00 to 1.96]	3.90	0.58 [0.27 to 1.25]
Smoker	No	93.10	93.60	ref	89.60	ref	95.50	ref
	Current	6.90	6.40	0.93 [0.24 to 3.63]	10.40	1.57 [0.75 to 3.27]	4.50	0.63 [0.30 to 1.32]
Main Ethnicity	White	67.70	53.80	ref	53.50	ref	62.50	ref
	Black	24.90	23.70	1.20 [0.58 to 2.49]	33.80	1.72 [1.15 to 2.57]	22.70	0.99 [0.64 to 1.51]
	Asian	3.40	9.10	3.38 [1.12 to 10.24]	8.00	3.00 [1.31 to 6.85]	8.10	2.58 [1.16 to 5.75]
	Other Ethnicity	4.00	13.40	4.23 [1.64 to 10.93]	4.70	1.49 [0.56 to 3.95]	6.70	1.81 [0.80 to 4.09]
Living with partner	Living with partner	79.50	61.40	ref	72.10	ref	79.50	ref
	Not Living with partner	20.50	38.60	2.45 [1.32 to 4.53]	27.90	1.51 [0.91 to 2.50]	20.50	1.00 [0.66 to 1.54]
Children < 2 years old	No child	85.70	88.50	ref	82.20	ref	88.70	ref
	One or more	14.30	11.50	0.78 [0.32 to 1.87]	17.80	1.29 [0.80 to 2.10]	11.30	0.76 [0.46 to 1.27]
Highest education attained	None	3.10	7.40	ref	8.30	ref	3.20	ref
	GCE	17.10	24.30	0.60 [0.16 to 2.27]	16.10	0.36 [0.15 to 0.86]	14.50	0.83 [0.30 to 2.35]
	Vocational	26.40	28.10	0.45 [0.10 to 2.09]	22.50	0.32 [0.14 to 0.76]	22.20	0.83 [0.30 to 2.27]
	A levels	15.00	13.20	0.37 [0.08 to 1.78]	15.20	0.38 [0.15 to 0.98]	16.90	1.11 [0.40 to 3.09]
	First degree	26.00	21.50	0.35 [0.09 to 1.43]	30.30	0.44 [0.19 to 1.04]	26.60	1.01 [0.37 to 2.75]
	Higher degree	12.40	5.50	0.19 [0.04 to 0.98]	7.50	0.23 [0.07 to 0.79]	16.60	1.31 [0.47 to 3.69]
BMI category	30-35	48.40	50.30	ref	46.00	ref	53.80	ref
	35-40	32.20	32.20	0.96 [0.49 to 1.90]	33.30	1.09 [0.71 to 1.66]	32.10	0.90 [0.62 to 1.28]
	>40	19.40	17.50	0.87 [0.38 to 2.00]	20.70	1.12 [0.68 to 1.86]	14.10	0.65 [0.40 to 1.08]
Folate intake at 1st visit	Daily	59.30	43.40	ref	52.90	ref	61.80	ref
	Less than daily	6.50	10.10	2.11 [0.70 to 6.30]	5.30	0.90 [0.40 to 2.06]	6.90	1.02 [0.54 to 1.92]
	Never	34.20	46.50	1.86 [0.97 to 3.56]	41.80	1.37 [0.91 to 2.08]	31.20	0.88 [0.62 to 1.25]
UK Born	No	27.70	38.90	ref	35.00	ref	36.50	ref
	Yes	72.30	61.10	0.60 [0.28 to 1.28]	65.00	0.71 [0.43 to 1.18]	63.50	0.67 [0.47 to 0.95]
Income	< £12,688	13.80	38.10	ref	26.60	ref	16.90	ref
	£12,688 - £17,628	11.40	11.30	0.36 [0.12 to 1.11]	14.20	0.64 [0.33 to 1.25]	10.70	0.77 [0.40 to 1.47]
	£17,629 - £23,452	9.50	5.30	0.20 [0.05 to 0.80]	9.80	0.53 [0.26 to 1.09]	6.30	0.54 [0.25 to 1.16]
	£23,453 - £32,500	11.20	15.20	0.49 [0.19 to 1.28]	11.50	0.53 [0.26 to 1.10]	13.70	1.00 [0.50 to 1.97]
	> £32,500	38.10	11.10	0.11 [0.03 to 0.34]	24.60	0.33 [0.18 to 0.62]	38.80	0.83 [0.49 to 1.41]
	Prefers not to answer	16.00	18.90	0.43 [0.16 to 1.14]	13.30	0.43 [0.20 to 0.92]	13.60	0.69 [0.32 to 1.48]
Parity	Nulliparous	41.20	34.60	ref	35.70	ref	51.30	ref
	Multiparous	58.80	65.40	1.32 [0.64 to 2.77]	64.30	1.26 [0.81 to 1.97]	48.70	0.67 [0.48 to 0.92]
Accomodation	Own house/flat	35.80	19.20	ref	24.70	ref	36.20	ref
	Temporary/other	1.90	5.10	4.88 [1.01 to 23.43]	2.30	1.72 [0.41 to 7.33]	3.20	1.65 [0.47 to 5.80]
	Family/friends free	5.90	10.80	3.40 [1.06 to 10.95]	6.80	1.67 [0.69 to 4.07]	5.10	0.85 [0.40 to 1.83]
	Private rental	30.30	29.00	1.78 [0.57 to 5.55]	33.50	1.60 [0.75 to 3.42]	31.00	1.01 [0.68 to 1.49]
	Council rental	26.00	35.80	2.57 [1.14 to 5.79]	32.60	1.82 [1.01 to 3.30]	24.40	0.93 [0.60 to 1.45]
Employement	Paid job	68.30	51.40	ref	56.40	ref	68.30	ref
	Looking after home/family	16.50	21.20	1.71 [0.76 to 3.85]	21.40	1.57 [0.98 to 2.52]	16.20	0.98 [0.61 to 1.59]
	Not in paid job	10.00	21.90	2.92 [1.25 to 6.81]	13.90	1.68 [0.96 to 2.94]	8.50	0.85 [0.42 to 1.73]
	School/Training	4.00	5.10	1.70 [0.45 to 6.42]	4.40	1.34 [0.55 to 3.27]	5.90	1.46 [0.64 to 3.36]
	Other	1.20	0.40	0.45 [0.00 to NA]	3.90	3.89 [1.08 to 13.99]	1.10	0.93 [0.06 to 14.73]
Asthma	No asthma	83.60	70.00	ref	80.30	ref	80.60	ref
	Asthmatic	16.40	30.00	2.18 [1.15 to 4.15]	19.70	1.25 [0.74 to 2.11]	19.40	1.23 [0.75 to 2.02]

Table S21: Baseline standardized mean difference vs the Not Depressed class

		Severe	Moderate	Mild
	Age	0.00 [-0.30 to 0.30]	-0.10 [-0.30 to 0.09]	-0.08 [-0.25 to 0.10]
Anthropometrics	BMI	-0.06 [-0.37 to 0.25]	0.02 [-0.17 to 0.21]	-0.07 [-0.24 to 0.11]
	Hip	0.10 [-0.21 to 0.42]	0.01 [-0.18 to 0.20]	-0.04 [-0.22 to 0.13]
	Neck	0.07 [-0.22 to 0.36]	-0.04 [-0.23 to 0.16]	-0.14 [-0.32 to 0.03]
	Thigh	-0.03 [-0.33 to 0.27]	0.03 [-0.16 to 0.23]	0.02 [-0.16 to 0.19]
	Waist/Hip	-0.03 [-0.32 to 0.26]	-0.03 [-0.22 to 0.16]	-0.09 [-0.27 to 0.09]
	Waist	0.07 [-0.24 to 0.38]	-0.02 [-0.20 to 0.17]	-0.11 [-0.29 to 0.07]
	Sum of skinfold	-0.14 [-0.44 to 0.16]	0.09 [-0.09 to 0.28]	0.05 [-0.13 to 0.22]
Latent	Obesity	-0.04 [-0.36 to 0.29]	0.02 [-0.17 to 0.22]	-0.07 [-0.25 to 0.11]
	SES	-0.77 [-1.13 to -0.42]	-0.41 [-0.64 to -0.17]	0.00 [-0.20 to 0.21]

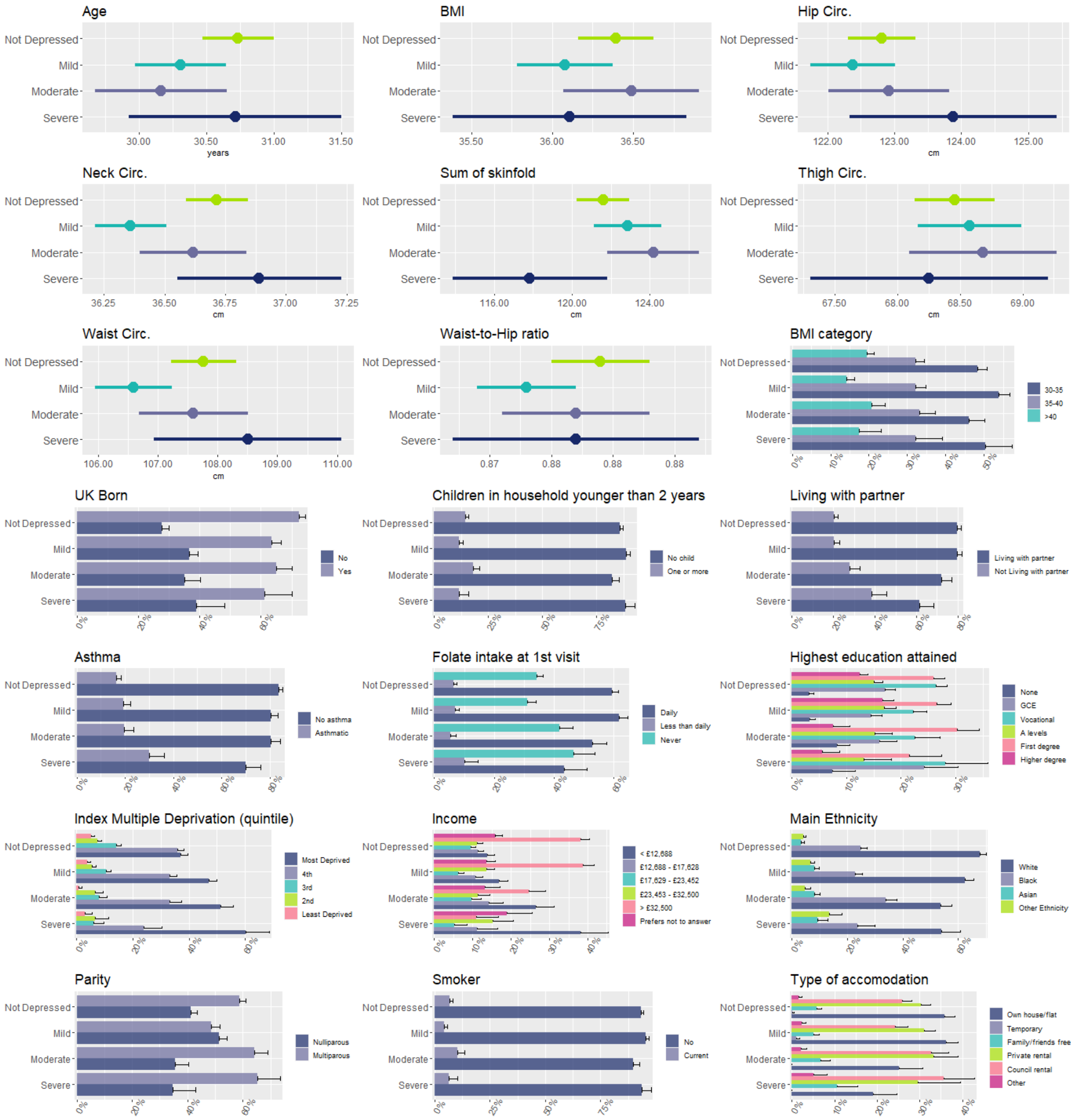

Figure S4: Participant characteristics by Class are presented as probabilities (%) for categorical variables and as means in original units for continuous variables, taking into account the fractional class membership error. All error bars are standard errors. Circ.= Circumference.

5.2 Latent measurement modelling

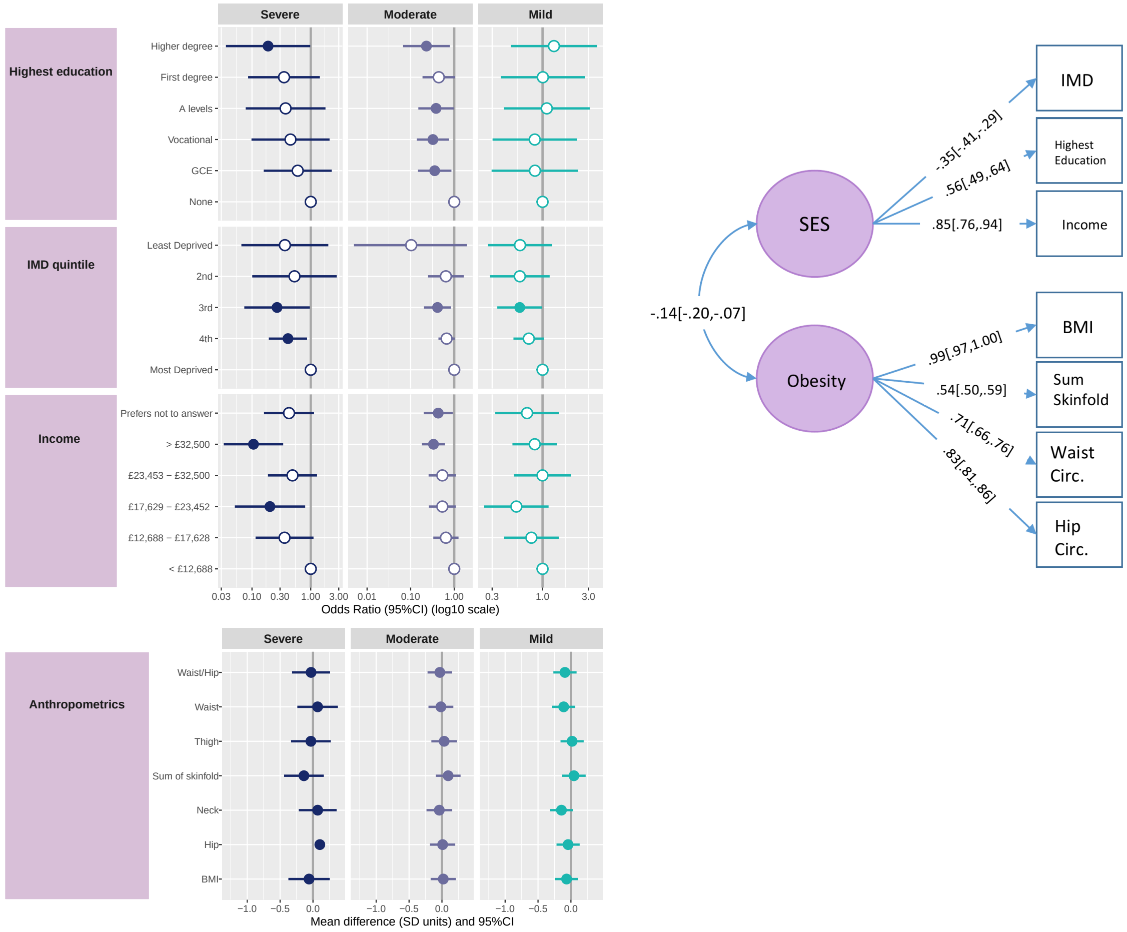

Figure S5: Plot presenting unadjusted Odds Ratios (ORs) and standardized differences of the means and their 95% Confidence intervals (CIs) of the associations between participant socio-economic status and anthropometrics against the reference ‘Not Depressed’ class. The variables were then used as indicators to model the latent constructs ‘SES’ and ‘Obesity’ used as covariates in adjusted models. On the right is the measurement model showing the standardized factor loadings[95%CI] and latent factors covariance (all significant at p<0.05).

5.3 Diet

Table S22: Unadjusted means [Standard Error] dietary intake in raw units.

	Severe	Moderate	Mild	Not Depressed
**17 weeks, trial entry**				
Energy (kcal)	2064.02 [124.35]	1961.93 [69.90]	1811.95 [40.12]	1784.01 [32.99]
Glycaemic load (/100g)	166.94 [11.02]	148.72 [5.96]	133.99 [3.62]	131.95 [2.82]
Saturated fat (g)	28.57 [2.21]	27.07 [1.31]	26.05 [0.74]	25.07 [0.63]
Total fat (% of Energy)	30.69 [0.97]	30.09 [0.61]	31.72 [0.36]	30.92 [0.31]
Carbohydrates (% of Energy)	52.13 [1.37]	50.63 [0.80]	48.44 [0.50]	48.90 [0.43]
Sugars (% of Energy)	26.17 [1.56]	25.12 [0.89]	23.57 [0.57]	23.63 [0.45]
Saturated fat (% of Energy)	12.33 [0.47]	12.34 [0.35]	12.90 [0.20]	12.49 [0.17]
Protein (% of Energy)	17.45 [0.74]	19.48 [0.47]	20.05 [0.32]	20.31 [0.25]
**27 weeks, trial end**				
Energy (kcal)	2022.85 [145.49]	1904.15 [64.79]	1670.46 [37.31]	1663.68 [28.96]
Glycaemic load (/100g)	148.40 [12.60]	141.27 [5.72]	115.67 [3.25]	120.27 [2.49]
Saturated fat (g)	27.92 [2.50]	26.42 [1.22]	24.32 [0.75]	23.32 [0.58]
Total fat (% of Energy)	31.62 [1.10]	30.18 [0.59]	31.92 [0.40]	30.63 [0.32]
Carbohydrates (% of Energy)	48.09 [1.66]	50.07 [0.82]	46.57 [0.56]	48.27 [0.40]
Sugars (% of Energy)	23.25 [1.76]	24.26 [0.90]	22.40 [0.58]	23.34 [0.44]
Saturated fat (% of Energy)	12.61 [0.56]	12.35 [0.36]	12.97 [0.22]	12.46 [0.18]
Protein (% of Energy)	20.42 [0.92]	19.93 [0.49]	21.63 [0.37]	21.16 [0.25]

Note:
Over- and under reporting in the dietary variables was controlled by excluding participant data if total calorie count >= 20Mj (=4,780kcal) or =< 4.5 Mj (=1,076 kcal).

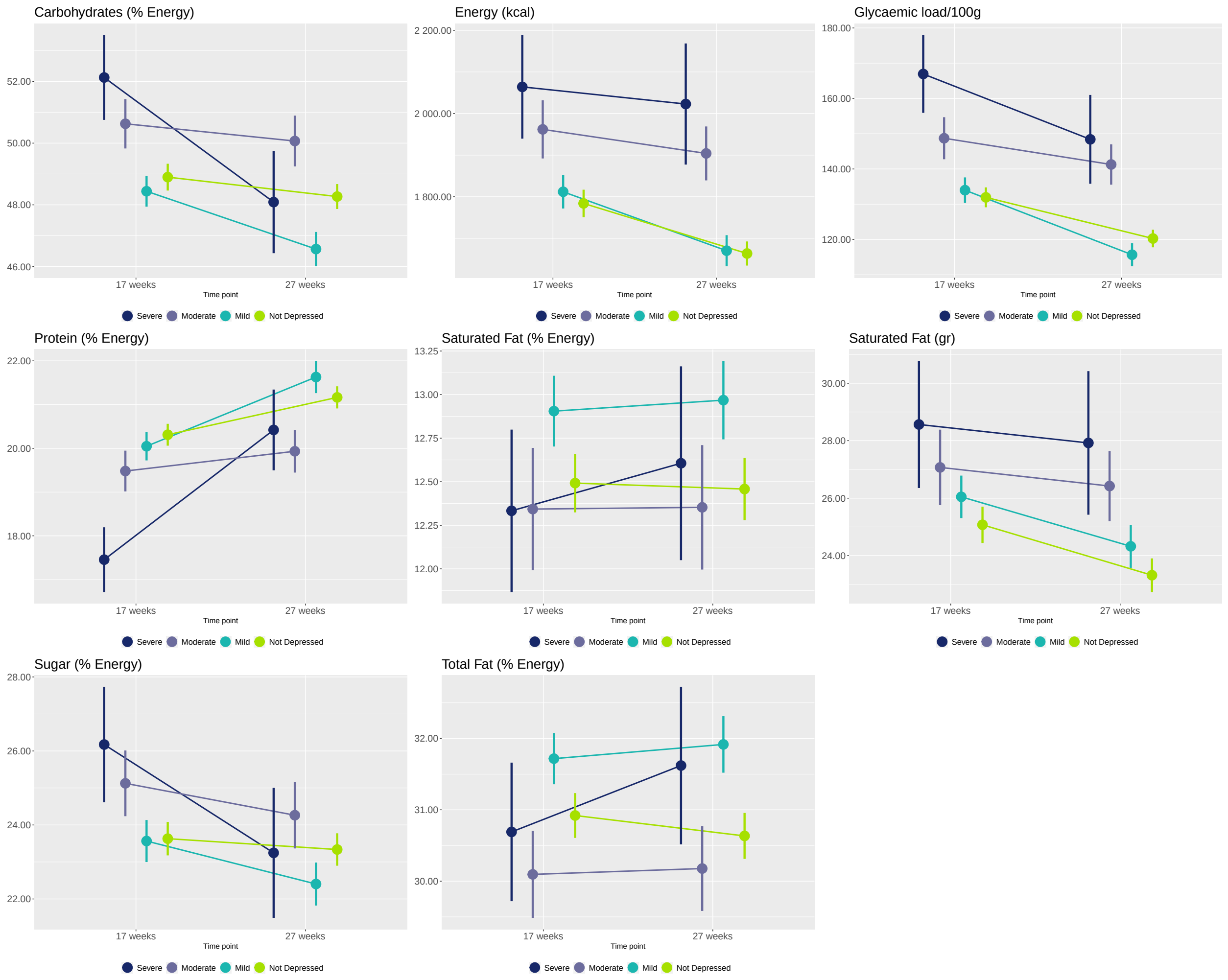

Figure S6: Women self-reported their dietary intake over the previous month at the first and second visit (at median 17[16 to 17] and median 27[27 to 28] weeks gestation respectively). Diet content and composition (% of total energy) were calculated and plotted as means and standard-errors, estimated for each class taking into account measurement error. Values are presented in raw units.

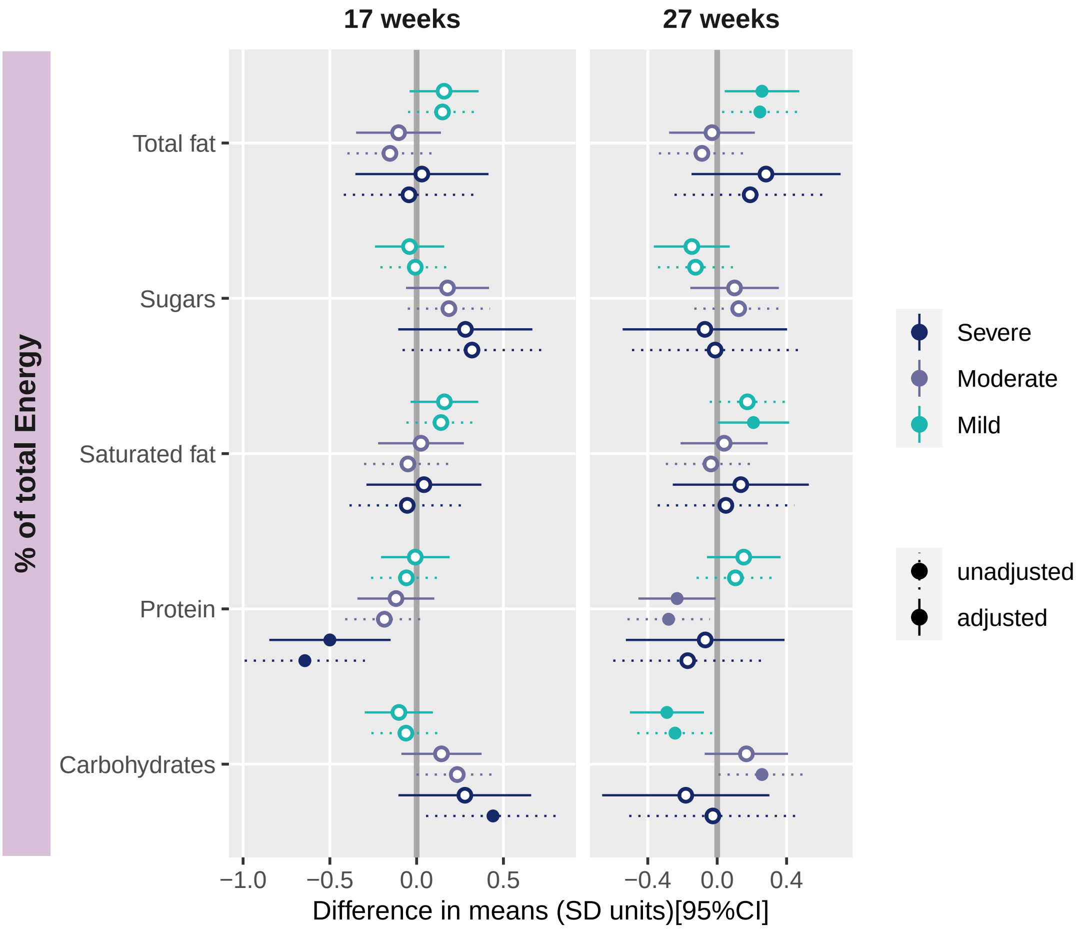

Figure S7: Classes were compared on their diet composition (as % of total Energy) against the Not Depressed class based on self-reported diet obtained at the trial entry (17 weeks) and trial end (27 weeks) which is presented in standardized mean differences from unadjusted and adjusted models with maternal age, nulliparity, white ethnicity, latent SES and latent Obesity as covariates, and the intervention effect at 27 weeks only. Circles are filled if the Confidence Intervals (CIs) did not contain 0, which was interpreted as significant.

Table S23: Mean differences in dietary intake and composition compared against the Not Depressed class in standardised units at 17 and 27 weeks gestation.

			Severe		Moderate		Mild	
Timepoint			MD[95%CI]	adjMD[95%CI]	MD[95%CI]	adjMD[95%CI]	MD[95%CI]	adjMD[95%CI]
17 weeks	% of total Energy	Carbohydrates	0.44[0.05 to 0.82]	0.28[-0.10 to 0.66]	0.23[0.00 to 0.47]	0.14[-0.09 to 0.37]	-0.06 [-0.26 to 0.14]	-0.10[-0.30 to 0.09]
		Protein	-0.64[-0.99 to -0.30]	-0.50[-0.85 to -0.15]	-0.19[-0.41 to 0.04]	-0.12[-0.34 to 0.10]	-0.06 [-0.26 to 0.14]	-0.01[-0.20 to 0.19]
		Saturated fat	-0.05[-0.39 to 0.28]	0.04[-0.29 to 0.37]	-0.05[-0.30 to 0.20]	0.03[-0.22 to 0.27]	0.14 [-0.06 to 0.34]	0.16[-0.04 to 0.36]
		Sugars	0.32[-0.08 to 0.72]	0.28[-0.11 to 0.67]	0.19[-0.05 to 0.42]	0.18[-0.06 to 0.42]	-0.01 [-0.21 to 0.19]	-0.04[-0.24 to 0.16]
		Total fat	-0.04[-0.42 to 0.33]	0.03[-0.35 to 0.41]	-0.15[-0.40 to 0.09]	-0.10[-0.35 to 0.14]	0.15 [-0.05 to 0.35]	0.16[-0.04 to 0.36]
	Content	Energy (kcal)	0.48[0.05 to 0.91]	0.33[-0.12 to 0.78]	0.30[0.05 to 0.55]	0.23[-0.02 to 0.48]	0.05 [-0.15 to 0.24]	0.04[-0.15 to 0.24]
		Glycaemic load/100g	0.68[0.25 to 1.11]	0.46[0.00 to 0.92]	0.32[0.08 to 0.57]	0.21[-0.03 to 0.45]	0.04 [-0.16 to 0.24]	0.02[-0.17 to 0.21]
		Saturated fat (g)	0.32[-0.09 to 0.73]	0.26[-0.16 to 0.68]	0.18[-0.07 to 0.43]	0.17[-0.09 to 0.42]	0.09 [-0.11 to 0.28]	0.10[-0.10 to 0.29]
27 weeks	% of total Energy	Carbohydrates	-0.03[-0.51 to 0.46]	-0.18[-0.66 to 0.30]	0.26[0.01 to 0.51]	0.17[-0.07 to 0.41]	-0.24 [-0.46 to -0.03]	-0.29[-0.50 to -0.08]
		Protein	-0.17[-0.60 to 0.26]	-0.07[-0.53 to 0.39]	-0.28[-0.52 to -0.04]	-0.23[-0.45 to -0.01]	0.10 [-0.12 to 0.33]	0.15[-0.06 to 0.36]
		Saturated fat	0.05[-0.34 to 0.44]	0.14[-0.26 to 0.53]	-0.04[-0.30 to 0.22]	0.04[-0.21 to 0.29]	0.17 [-0.04 to 0.39]	0.21[0.00 to 0.42]
		Sugars	-0.01[-0.49 to 0.47]	-0.07[-0.54 to 0.40]	0.12[-0.13 to 0.38]	0.10[-0.16 to 0.36]	-0.12 [-0.34 to 0.09]	-0.15[-0.36 to 0.07]
		Total fat	0.19[-0.25 to 0.63]	0.28[-0.15 to 0.71]	-0.09[-0.34 to 0.16]	-0.03[-0.28 to 0.22]	0.25 [0.03 to 0.46]	0.26[0.04 to 0.47]
	Content	Energy (kcal)	0.70[0.13 to 1.27]	0.60[0.04 to 1.17]	0.47[0.20 to 0.73]	0.42[0.16 to 0.68]	0.01 [-0.19 to 0.22]	0.04[-0.17 to 0.24]
		Glycaemic load/100g	0.64[0.07 to 1.20]	0.48[-0.07 to 1.03]	0.47[0.20 to 0.74]	0.39[0.14 to 0.65]	-0.10 [-0.31 to 0.10]	-0.10[-0.29 to 0.10]
		Saturated fat (g)	0.46[-0.04 to 0.97]	0.43[-0.07 to 0.93]	0.31[0.05 to 0.57]	0.32[0.07 to 0.57]	0.10 [-0.11 to 0.31]	0.14[-0.06 to 0.34]

Note:
AdjMD=adjusted mean difference, all presented in SD units, with maternal age, nulliparity (vs multiparity), white ethnicity (vs other), latent socio-economic status, latent obesity and intervention (at 27 weeks only) as covariates.

5.3.1 Main effects of covariates on diet

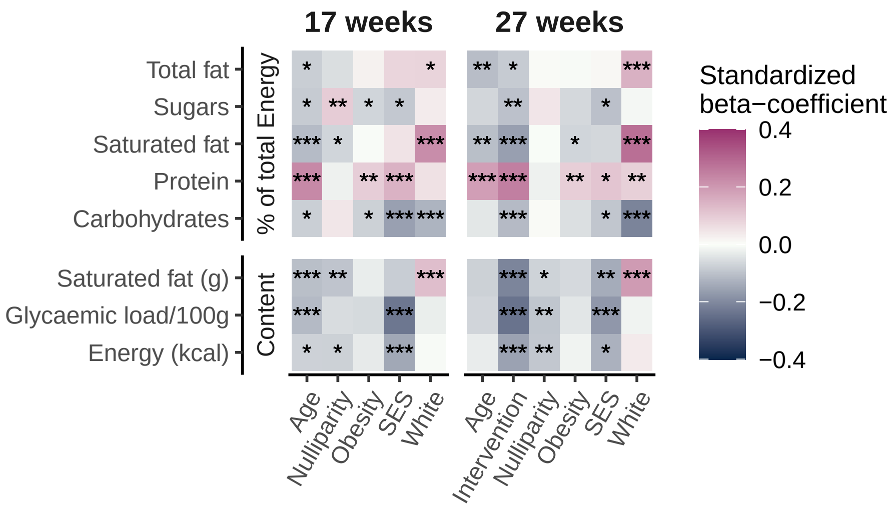

Figure S8: Main effects of covariates on dietary intake at 17 and 27 weeks. Maternal age, nulliparity (vs Multiparity), White (vs non-White), latent SES, latent Obesity and Intervention (vs Controls, included at 27 weeks only) were entered into multiple linear regression models for each dietary factor. Negative SES indicates more adverse SES. Heatmap colours represent the sign and estimate of the standardized coefficients which can be used to evaluate the influence of a covariate when the others are held constant. Variables significantly associated with diet macronutrient are annotated '*':p<0.05,'**':p<0.01,'***'p<0.001.

5.3.2 Interactions Covariates x Class

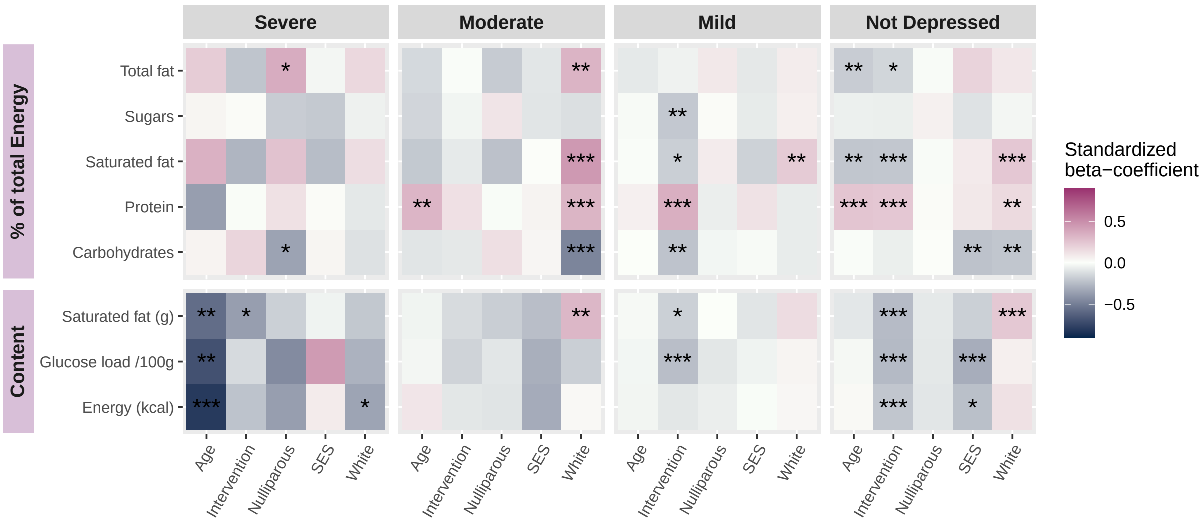

Figure S9: In order to explore the presence of an interaction between the depressive symptom classes and effect of the intervention, we conducted a within-class regressions with age, nulliparity, white (vs non-white) ethnicity and latent socio-economic status (with income, highest education attained and index of multiple deprivation as indicators). Over- and under reporting was controlled by excluding participants if calorie intake was less than 20Mj (=4,780kcal) and higher than 4.5 Mj (=1,076 kcal). The approximate count of available/missing data per class on diet at trial end was Depressed= 41/21, Subclinical= 164/55, Moderate = 376/137, Not Depressed = 445/130.

5.4 Blood markers

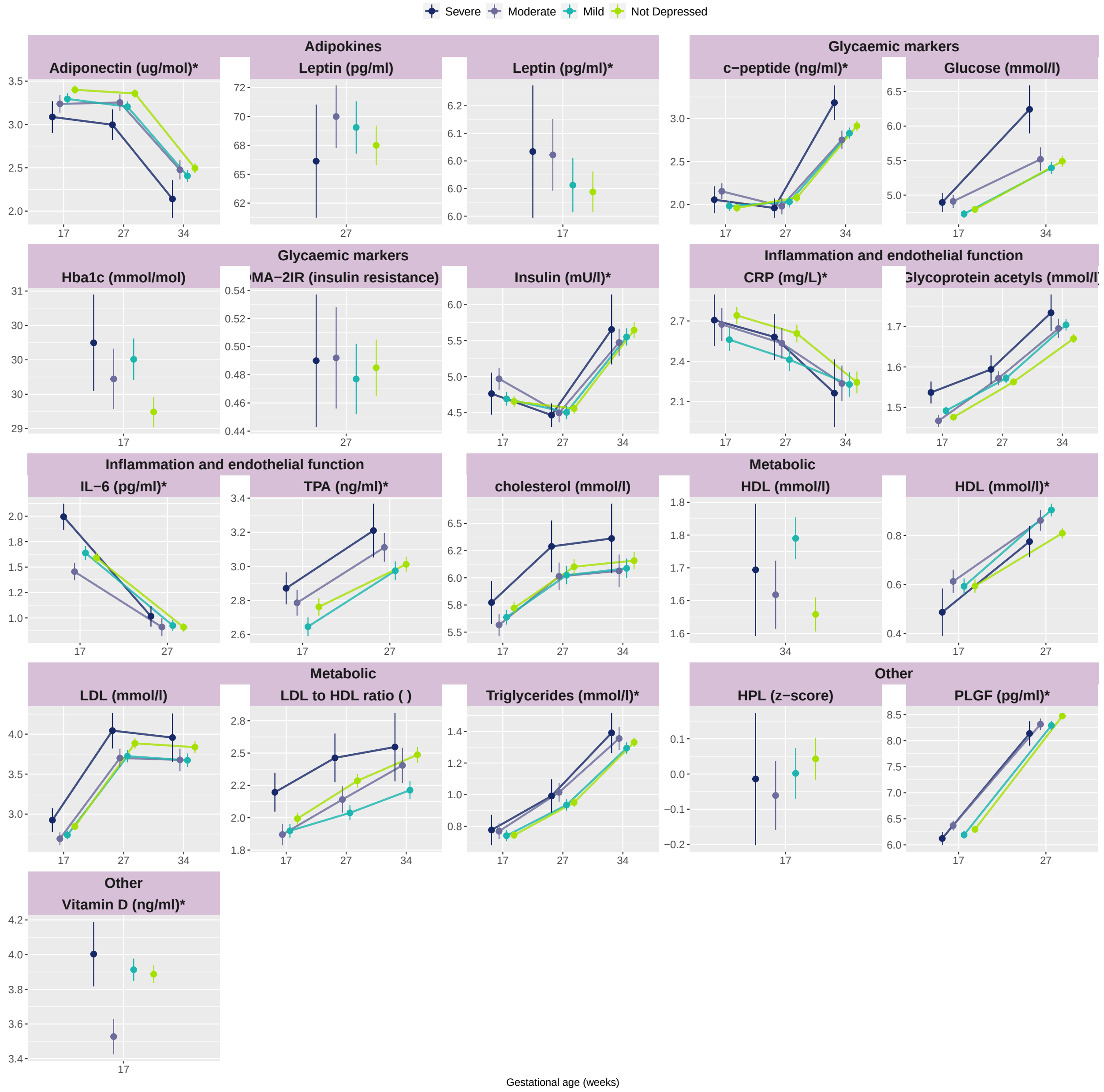

Figure S10: Participant unadjusted means and standard errors across classes in raw units at three time points in pregnancy taking into account the class membership measurement error. Random samples were taken at 17 and 34 weeks and from fasting at 27 weeks according to oral glucose tolerance test protocol. * presented after log transformation.

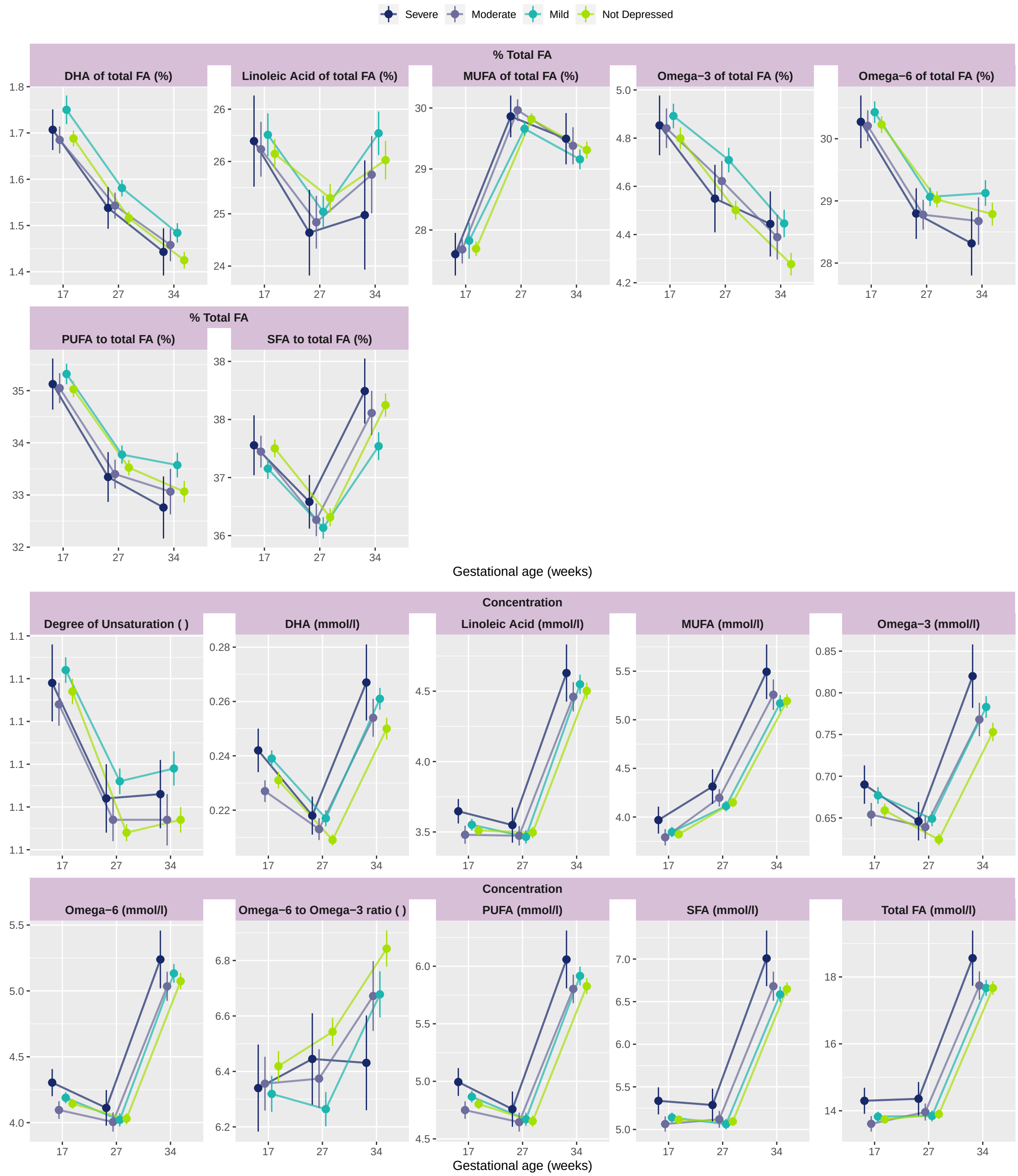

Figure S11: Plots of means(SE) fatty Acid concentration and as % total fatty acids at three time points across four classes taking into account the class membership measurement error. Random samples were taken at 17 and 34 weeks and from fasting at 27 weeks according to oral glucose tolerance test protocol. FA: Fatty Acids, MUFA:Mono-unsaturated FA, SFA: Saturated FA,PUFA: Poly-unsaturated FA, DHA:Docosahexaenoic acid.

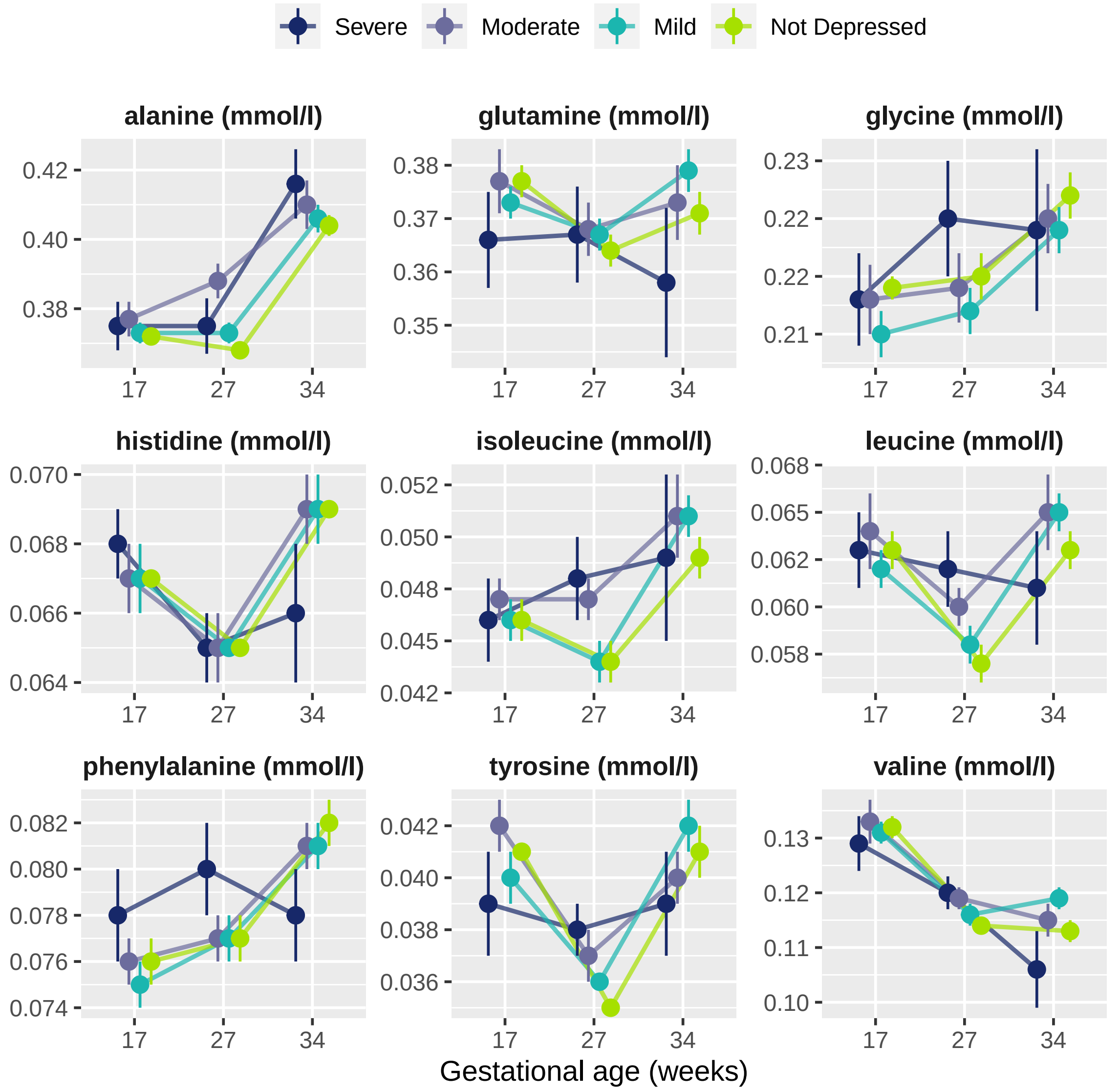

Figure S12: Means (Standard errors) in 9 Amino Acids measured at the three visits in unadjusted values estimated for the Severe, Moderate, Mild and Not Depressed classes. First and last sample taken from random blood, second after overnight fast in accordance at the OGTT visit.

5.4.1 Group comparisons on acute infection (CRP>10mg/L) at blood sampling

To exclude the presence of active infection at blood sampling which could affect other markers by comparing C-reactive protein levels above 10mg/L as the clinical cut-off.

Table S24: Class comparison on acute infection at each visit.

		17 weeks n= 969			27 weeks n= 894			34 weeks n=733		
		Prob (%)	OR[95%CI]	adjOR[95%CI]	Prob (%)	OR[95%CI]	adjOR[95%CI]	Prob (%)	OR[95%CI]	adjOR[95%CI]
Severe	CRP =<10 (mg/L)	70.50	ref	ref	82.30	ref	ref	81.30	ref	ref
	CRP >10 (mg/L)	29.50	1.01 [0.45 to 2.29]	0.74 [0.31 to 1.80]	17.70	0.86 [0.28 to 2.67]	0.58 [0.19 to 1.78]	18.70	1.03 [0.36 to 3.00]	0.98 [0.27 to 3.59]
Moderate	CRP =<10 (mg/L)	77.60	ref	ref	82.20	ref	ref	85.50	ref	ref
	CRP >10 (mg/L)	22.40	0.70 [0.29 to 1.66]	0.67 [0.38 to 1.20]	17.80	0.87 [0.45 to 1.67]	0.66 [0.35 to 1.24]	14.50	0.76 [0.38 to 1.52]	0.65 [0.28 to 1.49]
Mild	CRP =<10 (mg/L)	73.10	ref	ref	86.40	ref	ref	85.90	ref	ref
	CRP >10 (mg/L)	26.90	0.89 [0.55 to 1.44]	0.87 [0.53 to 1.41]	13.60	0.63 [0.35 to 1.15]	0.75 [0.44 to 1.29]	14.10	0.74 [0.41 to 1.31]	0.91 [0.47 to 1.77]
Not Depressed	CRP =<10 (mg/L)	70.80	ref	ref	80.10	ref	ref	81.80	ref	ref
	CRP >10 (mg/L)	29.20	ref	ref	19.90	ref	ref	18.20	ref	ref

Note:
Classes Odds ratios (ORs) and confidence intervals (CIs) are presented against the Not Depressed class as the reference class.

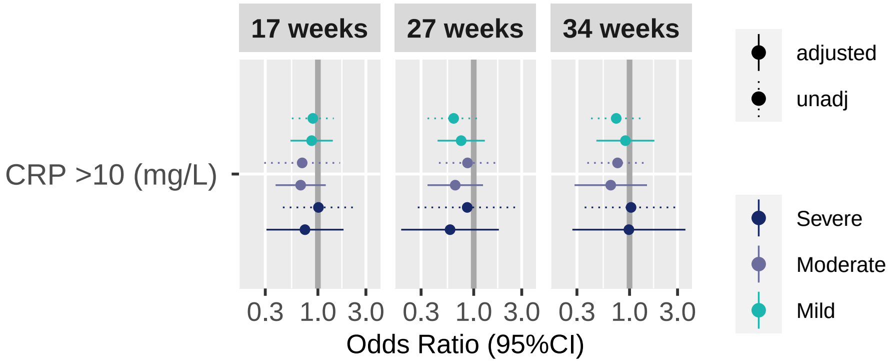

5.4.2 Group comparisons on blood biomarkers

Adipokines, Amino Acids and Fatty Acids were compared against the Not Depressed class. Metabolic/Glycaemic/Inflammatory/Placental/Vit-D markers are in the main manuscript.

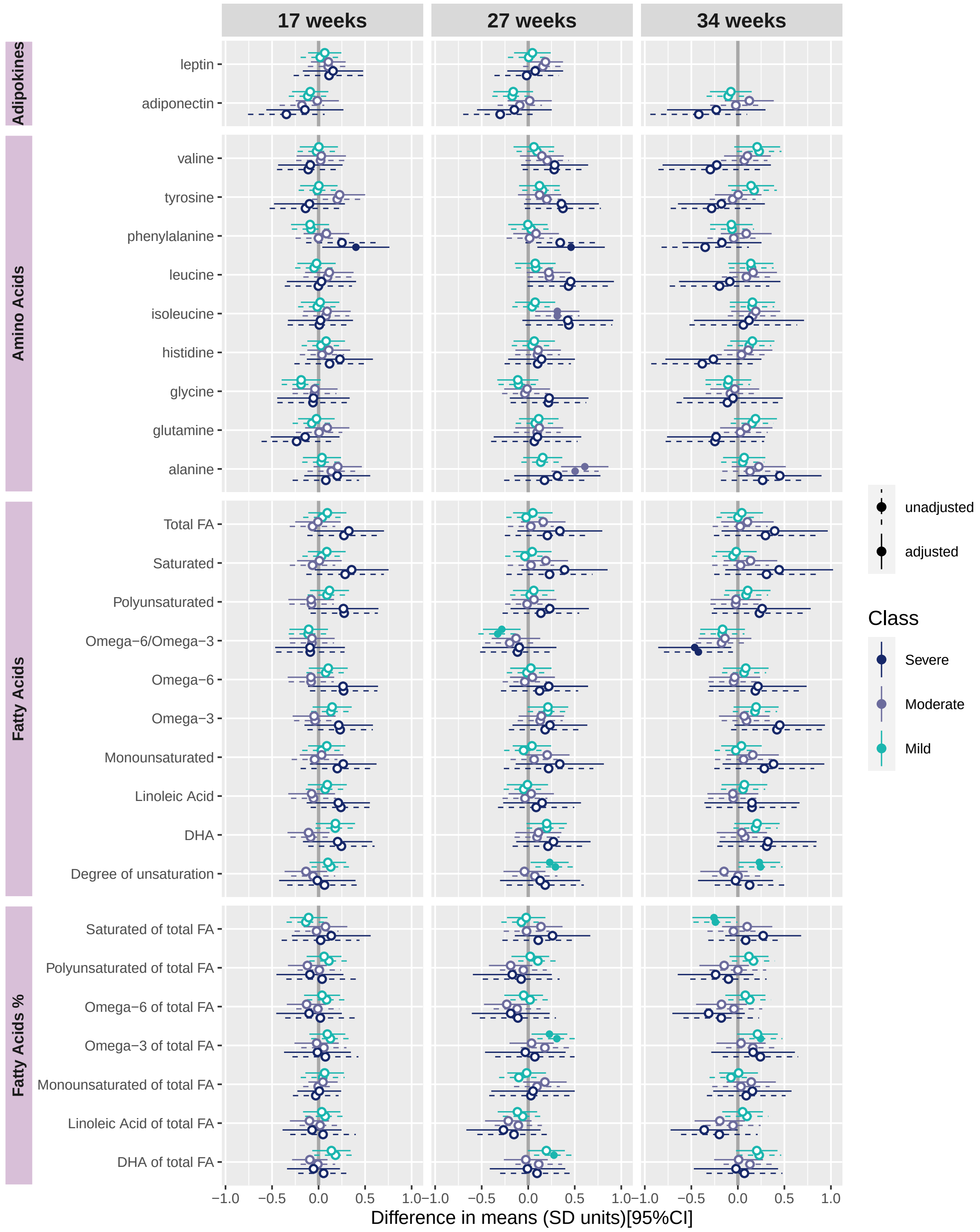

Figure S13: Blood markers were compared between the Severe, Moderate and Mild against the Not depressed class and presented as mean difference [95% CIs] in standardized units unadjusted and adjusted for age, nulliparity and white ethnicity, latent socio-economic status and latent obesity and the intervention at 27 and 34 weeks only. All estimates are calculated taking into account the class membership measurement error.

5.4.3 Tables at 17 weeks

Table S25: Mean differences in Adipokines Inflammation and endothelial function, Amino Acids, Placental,Glycaemic markers against the Not Depressed class in standardised units at 17 weeks gestation.

		Depressed		Subclinical		Moderate	
Group	Biomarker	MD[95%CI]	adjMD[95%CI]	MD[95%CI]	adjMD[95%CI]	MD[95%CI]	adjMD[95%CI]
Adipokines	adiponectin	-0.35[-0.76 to 0.06]	-0.15[-0.56 to 0.27]	-0.18[-0.42 to 0.06]	-0.01[-0.25 to 0.22]	-0.12 [-0.32 to 0.09]	-0.09[-0.28 to 0.10]
	leptin	0.11[-0.27 to 0.49]	0.16[-0.17 to 0.48]	0.10[-0.12 to 0.32]	0.10[-0.08 to 0.29]	0.02 [-0.19 to 0.23]	0.07[-0.11 to 0.24]
Amino Acids	alanine	0.08[-0.28 to 0.44]	0.20[-0.15 to 0.56]	0.14[-0.12 to 0.39]	0.21[-0.05 to 0.47]	0.03 [-0.18 to 0.23]	0.04[-0.17 to 0.24]
	glutamine	-0.24[-0.61 to 0.14]	-0.14[-0.51 to 0.22]	0.00[-0.24 to 0.26]	0.09[-0.14 to 0.33]	-0.07 [-0.28 to 0.13]	-0.02[-0.22 to 0.17]
	glycine	-0.06[-0.44 to 0.32]	-0.06[-0.44 to 0.33]	-0.04[-0.28 to 0.19]	-0.04[-0.28 to 0.20]	-0.19 [-0.40 to 0.02]	-0.19[-0.40 to 0.02]
	histidine	0.12[-0.26 to 0.50]	0.23[-0.13 to 0.58]	0.04[-0.20 to 0.28]	0.11[-0.12 to 0.34]	0.03 [-0.18 to 0.23]	0.08[-0.12 to 0.28]
	isoleucine	0.01[-0.33 to 0.35]	0.02[-0.33 to 0.37]	0.08[-0.17 to 0.33]	0.09[-0.16 to 0.34]	-0.01 [-0.22 to 0.19]	0.02[-0.19 to 0.22]
	leucine	0.00[-0.36 to 0.36]	0.03[-0.34 to 0.40]	0.10[-0.16 to 0.36]	0.12[-0.14 to 0.38]	-0.05 [-0.25 to 0.16]	-0.02[-0.23 to 0.18]
	phenylalanine	0.25[-0.13 to 0.64]	0.40[0.04 to 0.76]	0.00[-0.25 to 0.25]	0.08[-0.16 to 0.33]	-0.08 [-0.29 to 0.12]	-0.09[-0.30 to 0.11]
	tyrosine	-0.14[-0.53 to 0.25]	-0.10[-0.48 to 0.28]	0.20[-0.07 to 0.47]	0.23[-0.05 to 0.50]	-0.01 [-0.21 to 0.19]	0.00[-0.20 to 0.20]
	valine	-0.11[-0.45 to 0.23]	-0.09[-0.44 to 0.26]	0.02[-0.24 to 0.28]	0.03[-0.24 to 0.29]	-0.02 [-0.23 to 0.18]	0.00[-0.20 to 0.21]
Glycaemic markers	c-peptide	0.12[-0.27 to 0.51]	0.06[-0.34 to 0.45]	0.23[-0.01 to 0.48]	0.18[-0.06 to 0.42]	0.03 [-0.18 to 0.23]	0.03[-0.17 to 0.23]
	glucose	0.13[-0.24 to 0.50]	0.04[-0.34 to 0.42]	0.15[-0.11 to 0.41]	0.09[-0.17 to 0.35]	-0.08 [-0.29 to 0.12]	-0.10[-0.30 to 0.10]
	hba1c	0.26[-0.12 to 0.64]	0.10[-0.31 to 0.51]	0.12[-0.12 to 0.37]	0.00[-0.23 to 0.22]	0.20 [-0.02 to 0.41]	0.20[-0.01 to 0.40]
	insulin	0.08[-0.36 to 0.52]	-0.04[-0.49 to 0.42]	0.23[-0.01 to 0.47]	0.12[-0.12 to 0.36]	0.03 [-0.17 to 0.23]	0.00[-0.19 to 0.20]
Inflammation and endothelial function	CRP	-0.03[-0.38 to 0.32]	-0.06[-0.41 to 0.29]	-0.06[-0.29 to 0.17]	-0.07[-0.31 to 0.16]	-0.16 [-0.37 to 0.05]	-0.10[-0.30 to 0.10]
	IL-6	0.45[0.14 to 0.75]	0.41[0.11 to 0.72]	-0.15[-0.36 to 0.06]	-0.19[-0.39 to 0.02]	0.05 [-0.16 to 0.27]	0.06[-0.15 to 0.27]
	tPA-antigen	0.14[-0.13 to 0.41]	0.05[-0.21 to 0.32]	0.03[-0.19 to 0.25]	-0.02[-0.24 to 0.21]	-0.15 [-0.36 to 0.06]	-0.16[-0.37 to 0.05]
	Glycoprotein acetyls	0.44[0.04 to 0.85]	0.44[0.06 to 0.82]	-0.06[-0.30 to 0.17]	-0.06[-0.30 to 0.18]	0.12 [-0.09 to 0.33]	0.16[-0.05 to 0.37]
Other	HPL	-0.06[-0.46 to 0.34]	-0.01[-0.38 to 0.37]	-0.11[-0.33 to 0.12]	-0.03[-0.26 to 0.19]	-0.04 [-0.25 to 0.17]	-0.04[-0.25 to 0.17]
	Plgf	-0.19[-0.49 to 0.10]	-0.40[-0.70 to -0.10]	0.08[-0.14 to 0.30]	-0.07[-0.29 to 0.16]	-0.12 [-0.33 to 0.10]	-0.13[-0.34 to 0.08]
	Vit-D	0.13[-0.30 to 0.56]	0.34[-0.08 to 0.75]	-0.40[-0.64 to -0.16]	-0.23[-0.46 to 0.01]	0.03 [-0.17 to 0.23]	0.04[-0.15 to 0.23]

Note:
AdjMD=adjusted mean difference, all presented in SD units, with maternal age, nulliparity (vs multiparity), white ethnicity (vs other), latent socio-economic status, latent obesity as covariates.

Table S26: Mean differences in Fatty Acids, Fatty Acids (of Total Fatty acids) and Metabolic markers against the Not Depressed class in standardised units at 17 weeks gestation.

Group	Biomarker	Severe		Moderate		Mild	
		MD[95%CI]	adjMD[95%CI]	MD[95%CI]	adjMD[95%CI]	MD[95%CI]	adjMD[95%CI]
Fatty Acids	DHA	0.24[-0.11 to 0.60]	0.20[-0.17 to 0.58]	-0.09[-0.30 to 0.13]	-0.11[-0.33 to 0.12]	0.18 [-0.03 to 0.39]	0.18[-0.03 to 0.39]
	Omega-3	0.23[-0.12 to 0.58]	0.22[-0.15 to 0.58]	-0.04[-0.26 to 0.19]	-0.05[-0.28 to 0.18]	0.13 [-0.08 to 0.34]	0.15[-0.06 to 0.36]
	Omega-6	0.27[-0.09 to 0.63]	0.26[-0.11 to 0.64]	-0.08[-0.32 to 0.16]	-0.08[-0.33 to 0.17]	0.08 [-0.13 to 0.28]	0.10[-0.10 to 0.31]
	Linoleic Acid	0.24[-0.09 to 0.57]	0.21[-0.13 to 0.55]	-0.05[-0.30 to 0.19]	-0.07[-0.33 to 0.18]	0.07 [-0.14 to 0.28]	0.09[-0.12 to 0.30]
	Monounsaturated	0.20[-0.19 to 0.60]	0.26[-0.09 to 0.62]	-0.04[-0.29 to 0.20]	0.03[-0.20 to 0.27]	0.03 [-0.18 to 0.24]	0.09[-0.11 to 0.29]
	Polyunsaturated	0.28[-0.09 to 0.64]	0.27[-0.11 to 0.64]	-0.08[-0.31 to 0.16]	-0.08[-0.32 to 0.17]	0.09 [-0.12 to 0.30]	0.12[-0.09 to 0.33]
	Saturated	0.28[-0.13 to 0.70]	0.36[-0.04 to 0.75]	-0.06[-0.31 to 0.18]	0.01[-0.23 to 0.25]	0.03 [-0.17 to 0.24]	0.09[-0.12 to 0.29]
	Total FA	0.27[-0.12 to 0.67]	0.32[-0.05 to 0.70]	-0.06[-0.31 to 0.18]	-0.01[-0.25 to 0.24]	0.04 [-0.17 to 0.25]	0.09[-0.11 to 0.30]
	Degree of unsaturation	0.06[-0.34 to 0.47]	-0.01[-0.42 to 0.40]	-0.06[-0.30 to 0.18]	-0.14[-0.36 to 0.09]	0.13 [-0.08 to 0.34]	0.10[-0.10 to 0.30]
	Omega-6/Omega-3	-0.09[-0.46 to 0.28]	-0.09[-0.47 to 0.28]	-0.07[-0.31 to 0.16]	-0.07[-0.32 to 0.17]	-0.11 [-0.32 to 0.10]	-0.11[-0.31 to 0.10]
Fatty Acids %	DHA of total FA	0.05[-0.22 to 0.33]	-0.05[-0.34 to 0.23]	-0.01[-0.19 to 0.18]	-0.09[-0.28 to 0.10]	0.18 [-0.05 to 0.41]	0.14[-0.07 to 0.34]
	Omega-3 of total FA	0.07[-0.28 to 0.43]	-0.01[-0.37 to 0.35]	0.06[-0.19 to 0.30]	-0.02[-0.26 to 0.22]	0.13 [-0.08 to 0.33]	0.09[-0.10 to 0.29]
	Omega-6 of total FA	0.02[-0.36 to 0.40]	-0.10[-0.46 to 0.25]	-0.01[-0.24 to 0.22]	-0.13[-0.34 to 0.08]	0.09 [-0.12 to 0.30]	0.04[-0.16 to 0.23]
	Linoleic Acid of total FA	0.05[-0.30 to 0.40]	-0.07[-0.39 to 0.25]	0.02[-0.20 to 0.24]	-0.10[-0.31 to 0.11]	0.07 [-0.14 to 0.29]	0.03[-0.17 to 0.23]
	Monounsaturated of total FA	-0.03[-0.28 to 0.22]	0.01[-0.23 to 0.24]	0.00[-0.17 to 0.16]	0.05[-0.11 to 0.20]	0.04 [-0.19 to 0.28]	0.06[-0.14 to 0.27]
	Polyunsaturated of total FA	0.04[-0.35 to 0.42]	-0.09[-0.45 to 0.27]	0.01[-0.23 to 0.24]	-0.12[-0.33 to 0.09]	0.11 [-0.10 to 0.32]	0.06[-0.13 to 0.25]
	Saturated of total FA	0.02[-0.40 to 0.44]	0.14[-0.29 to 0.56]	-0.02[-0.26 to 0.21]	0.07[-0.16 to 0.31]	-0.14 [-0.34 to 0.07]	-0.11[-0.31 to 0.10]
Metabolic	cholesterol	0.05[-0.36 to 0.47]	0.10[-0.34 to 0.53]	-0.16[-0.39 to 0.07]	-0.12[-0.35 to 0.11]	-0.09 [-0.29 to 0.12]	-0.06[-0.27 to 0.15]
	HDL	-0.24[-0.68 to 0.20]	-0.22[-0.65 to 0.22]	0.04[-0.19 to 0.27]	0.01[-0.21 to 0.24]	0.00 [-0.20 to 0.20]	-0.03[-0.23 to 0.17]
	LDL	0.10[-0.29 to 0.50]	0.16[-0.24 to 0.55]	-0.20[-0.43 to 0.03]	-0.15[-0.38 to 0.08]	-0.14 [-0.34 to 0.07]	-0.10[-0.31 to 0.10]
	LDL/HDL	0.27[-0.14 to 0.67]	0.26[-0.11 to 0.63]	-0.16[-0.39 to 0.07]	-0.12[-0.34 to 0.10]	-0.12 [-0.33 to 0.08]	-0.08[-0.29 to 0.12]
	triglycerides	0.07[-0.35 to 0.49]	0.03[-0.39 to 0.46]	0.05[-0.17 to 0.28]	0.07[-0.16 to 0.30]	-0.01 [-0.21 to 0.20]	0.03[-0.17 to 0.24]

Note:
AdjMD= adjusted mean difference, all presented in SD units, with maternal age, nulliparity (vs multiparity), white ethnicity (vs other), latent socio-economic status, latent obesity as covariates.

5.4.4 Tables at 27 weeks

Table S27: Mean differences in Adipokines Inflammation and endothelial function, Amino Acids, Placental, Glycaemic markers against the Not Depressed class in standardised units at 27 weeks gestation.

Group	Biomarker	Severe		Moderate		Mild	
		MD[95%CI]	adjMD[95%CI]	MD[95%CI]	adjMD[95%CI]	MD[95%CI]	adjMD[95%CI]
Adipokines	adiponectin	-0.30[-0.70 to 0.10]	-0.15[-0.55 to 0.25]	-0.09[-0.33 to 0.15]	0.02[-0.22 to 0.25]	-0.17 [-0.39 to 0.04]	-0.16[-0.38 to 0.05]
	leptin	-0.02[-0.36 to 0.33]	0.07[-0.22 to 0.38]	0.16[-0.06 to 0.37]	0.18[0.00 to 0.37]	0.00 [-0.22 to 0.23]	0.04[-0.15 to 0.24]
Amino Acids	alanine	0.17[-0.26 to 0.61]	0.31[-0.15 to 0.78]	0.50[0.25 to 0.76]	0.61[0.35 to 0.86]	0.14 [-0.07 to 0.34]	0.16[-0.05 to 0.37]
	glutamine	0.07[-0.40 to 0.53]	0.10[-0.37 to 0.57]	0.10[-0.16 to 0.35]	0.12[-0.14 to 0.38]	0.07 [-0.13 to 0.28]	0.11[-0.10 to 0.33]
	glycine	0.22[-0.19 to 0.62]	0.22[-0.20 to 0.65]	-0.04[-0.28 to 0.21]	-0.01[-0.26 to 0.23]	-0.10 [-0.32 to 0.11]	-0.11[-0.33 to 0.11]
	histidine	0.10[-0.25 to 0.46]	0.14[-0.22 to 0.50]	0.10[-0.14 to 0.34]	0.11[-0.14 to 0.35]	0.04 [-0.18 to 0.26]	0.06[-0.16 to 0.29]
	isoleucine	0.44[-0.03 to 0.90]	0.42[-0.06 to 0.91]	0.31[0.07 to 0.55]	0.31[0.07 to 0.55]	0.04 [-0.17 to 0.25]	0.07[-0.14 to 0.29]
	leucine	0.43[-0.01 to 0.87]	0.46[-0.01 to 0.92]	0.23[-0.01 to 0.46]	0.22[-0.02 to 0.46]	0.08 [-0.13 to 0.30]	0.07[-0.14 to 0.30]
	phenylalanine	0.34[-0.03 to 0.72]	0.46[0.10 to 0.82]	0.01[-0.23 to 0.26]	0.08[-0.16 to 0.33]	0.03 [-0.18 to 0.25]	0.00[-0.22 to 0.21]
	tyrosine	0.37[-0.04 to 0.78]	0.36[-0.04 to 0.76]	0.20[-0.03 to 0.43]	0.12[-0.11 to 0.35]	0.15 [-0.06 to 0.37]	0.12[-0.10 to 0.34]
	valine	0.28[-0.06 to 0.62]	0.28[-0.08 to 0.64]	0.20[-0.03 to 0.44]	0.15[-0.09 to 0.38]	0.09 [-0.13 to 0.31]	0.06[-0.16 to 0.28]
Glycaemic markers	c-peptide	-0.14[-0.43 to 0.15]	-0.02[-0.32 to 0.28]	-0.12[-0.36 to 0.13]	0.02[-0.22 to 0.25]	-0.06 [-0.28 to 0.16]	-0.01[-0.22 to 0.21]
	insulin	-0.07[-0.37 to 0.22]	-0.04[-0.34 to 0.27]	-0.05[-0.28 to 0.18]	-0.01[-0.24 to 0.23]	-0.04 [-0.26 to 0.17]	-0.04[-0.26 to 0.19]
Inflammation and endothelial function	CRP	-0.02[-0.36 to 0.31]	-0.01[-0.34 to 0.32]	-0.07[-0.30 to 0.16]	-0.04[-0.27 to 0.19]	-0.18 [-0.40 to 0.04]	-0.12[-0.34 to 0.09]
	IL-6	0.15[-0.15 to 0.44]	0.00[-0.29 to 0.30]	0.00[-0.25 to 0.26]	-0.07[-0.31 to 0.17]	0.02 [-0.19 to 0.24]	0.04[-0.16 to 0.25]
	tPA-antigen	0.27[-0.17 to 0.70]	0.23[-0.22 to 0.68]	0.13[-0.11 to 0.38]	0.12[-0.13 to 0.37]	-0.05 [-0.26 to 0.16]	-0.07[-0.29 to 0.15]
	Glycoprotein acetyls	0.22[-0.27 to 0.70]	0.25[-0.25 to 0.74]	0.06[-0.19 to 0.31]	0.13[-0.11 to 0.37]	0.06 [-0.15 to 0.27]	0.13[-0.08 to 0.34]
Other	Plgf	-0.31[-0.75 to 0.13]	-0.49[-0.92 to -0.05]	-0.14[-0.37 to 0.08]	-0.27[-0.50 to -0.04]	-0.17 [-0.38 to 0.05]	-0.16[-0.37 to 0.06]

Note:
AdjMD=adjusted mean difference, all presented in SD units, with maternal age, nulliparity (vs multiparity), white ethnicity (vs other), latent socio-economic status, latent obesity and intervention as covariates.

Table S28: Mean differences in Fatty Acids, Fatty Acids (of Total Fatty acids) and Metabolic markers against the Not Depressed class in standardised units at 27 weeks gestation.

Group	Biomarker	Severe		Moderate		Mild	
		MD[95%CI]	adjMD[95%CI]	MD[95%CI]	adjMD[95%CI]	MD[95%CI]	adjMD[95%CI]
Fatty Acids	DHA	0.21[-0.17 to 0.60]	0.27[-0.13 to 0.67]	0.10[-0.15 to 0.34]	0.11[-0.14 to 0.36]	0.20 [-0.01 to 0.42]	0.20[-0.02 to 0.42]
	Omega-3	0.18[-0.21 to 0.57]	0.23[-0.17 to 0.64]	0.13[-0.11 to 0.37]	0.14[-0.10 to 0.38]	0.21 [0.00 to 0.43]	0.21[-0.01 to 0.43]
	Omega-6	0.12[-0.29 to 0.54]	0.22[-0.20 to 0.64]	-0.04[-0.28 to 0.20]	0.04[-0.20 to 0.29]	-0.01 [-0.23 to 0.20]	0.03[-0.19 to 0.25]
	Linoleic Acid	0.09[-0.32 to 0.50]	0.15[-0.27 to 0.57]	-0.04[-0.28 to 0.21]	0.03[-0.21 to 0.28]	-0.05 [-0.26 to 0.17]	-0.01[-0.23 to 0.21]
	Monounsaturated	0.22[-0.26 to 0.70]	0.34[-0.14 to 0.81]	0.06[-0.19 to 0.32]	0.20[-0.04 to 0.44]	-0.05 [-0.26 to 0.16]	0.04[-0.17 to 0.25]
	Polyunsaturated	0.14[-0.28 to 0.55]	0.23[-0.19 to 0.65]	-0.01[-0.25 to 0.22]	0.06[-0.18 to 0.30]	0.02 [-0.20 to 0.24]	0.06[-0.16 to 0.28]
	Saturated	0.23[-0.23 to 0.69]	0.39[-0.07 to 0.85]	0.03[-0.22 to 0.28]	0.19[-0.05 to 0.43]	-0.04 [-0.25 to 0.17]	0.04[-0.16 to 0.25]
	Total FA	0.21[-0.25 to 0.66]	0.34[-0.12 to 0.80]	0.03[-0.22 to 0.27]	0.16[-0.08 to 0.40]	-0.02 [-0.24 to 0.19]	0.05[-0.16 to 0.26]
	Degree of unsaturation	0.18[-0.23 to 0.60]	0.13[-0.30 to 0.56]	0.07[-0.17 to 0.31]	-0.04[-0.27 to 0.18]	0.29 [0.08 to 0.51]	0.23[0.03 to 0.44]
	Omega-6/Omega-3	-0.12[-0.52 to 0.29]	-0.10[-0.50 to 0.30]	-0.20[-0.46 to 0.06]	-0.13[-0.39 to 0.13]	-0.33 [-0.54 to -0.12]	-0.28[-0.49 to -0.08]
Fatty Acids %	DHA of total FA	0.10[-0.30 to 0.49]	-0.01[-0.41 to 0.40]	0.11[-0.14 to 0.36]	-0.03[-0.26 to 0.21]	0.28 [0.06 to 0.49]	0.20[0.00 to 0.39]
	Omega-3 of total FA	0.07[-0.36 to 0.50]	-0.03[-0.46 to 0.40]	0.18[-0.08 to 0.44]	0.04[-0.20 to 0.27]	0.31 [0.10 to 0.52]	0.23[0.04 to 0.42]
	Omega-6 of total FA	-0.11[-0.52 to 0.30]	-0.19[-0.61 to 0.23]	-0.12[-0.37 to 0.13]	-0.23[-0.48 to 0.01]	0.02 [-0.19 to 0.23]	-0.05[-0.26 to 0.16]
	Linoleic Acid of total FA	-0.15[-0.55 to 0.24]	-0.27[-0.66 to 0.13]	-0.11[-0.36 to 0.15]	-0.21[-0.46 to 0.04]	-0.06 [-0.27 to 0.15]	-0.12[-0.33 to 0.10]
	Monounsaturated of total FA	0.03[-0.42 to 0.48]	0.05[-0.40 to 0.50]	0.09[-0.15 to 0.34]	0.18[-0.05 to 0.41]	-0.10 [-0.31 to 0.11]	-0.02[-0.22 to 0.19]
	Polyunsaturated of total FA	-0.08[-0.49 to 0.34]	-0.17[-0.60 to 0.25]	-0.05[-0.30 to 0.20]	-0.19[-0.42 to 0.04]	0.10 [-0.11 to 0.32]	0.02[-0.18 to 0.22]
	Saturated of total FA	0.11[-0.28 to 0.49]	0.26[-0.14 to 0.67]	-0.02[-0.26 to 0.23]	0.14[-0.09 to 0.37]	-0.07 [-0.29 to 0.14]	-0.02[-0.23 to 0.18]
Metabolic	cholesterol	0.16[-0.26 to 0.59]	0.31[-0.12 to 0.74]	-0.08[-0.32 to 0.16]	0.01[-0.23 to 0.26]	-0.07 [-0.28 to 0.15]	-0.03[-0.25 to 0.19]
	HDL	-0.10[-0.47 to 0.28]	0.00[-0.37 to 0.36]	0.14[-0.11 to 0.40]	0.14[-0.10 to 0.37]	0.26 [0.05 to 0.48]	0.22[0.00 to 0.43]
	LDL	0.15[-0.28 to 0.59]	0.27[-0.16 to 0.70]	-0.17[-0.42 to 0.07]	-0.08[-0.32 to 0.16]	-0.15 [-0.36 to 0.06]	-0.11[-0.32 to 0.11]
	LDL/HDL	0.21[-0.24 to 0.67]	0.22[-0.20 to 0.64]	-0.17[-0.43 to 0.09]	-0.11[-0.35 to 0.14]	-0.29 [-0.50 to -0.09]	-0.24[-0.45 to -0.03]
	triglycerides	0.15[-0.29 to 0.59]	0.16[-0.28 to 0.61]	0.18[-0.08 to 0.44]	0.25[-0.01 to 0.50]	-0.03 [-0.24 to 0.17]	0.03[-0.18 to 0.24]

Note:
AdjMD= adjusted mean difference, all presented in SD units, with maternal age, nulliparity (vs multiparity), white ethnicity (vs other), latent socio-economic status, latent obesity and intervention as covariates.

5.4.5 Interaction class x covariates

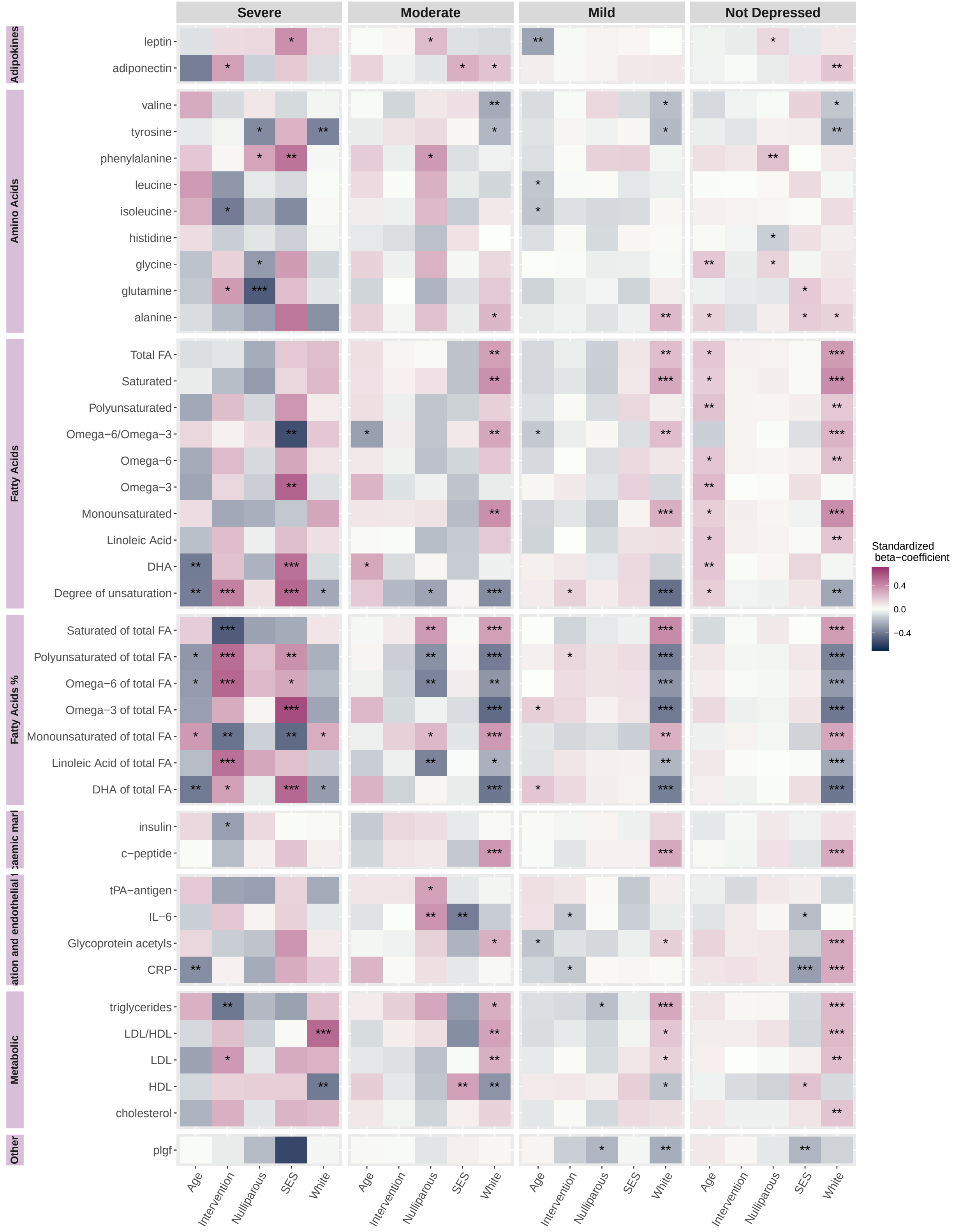

Figure S14: In order to explore an interaction between depressive symptom class and covariates, we performed a within-class regressions. Blood samples at the second visit were taken after overnight fast during the oral glucose tolerance test. Variables significantly associated with metabolites are annotated ”:p<0.05,’*’:p<0.01,’**’p<0.001.

5.4.6 Sensitivity Analysis

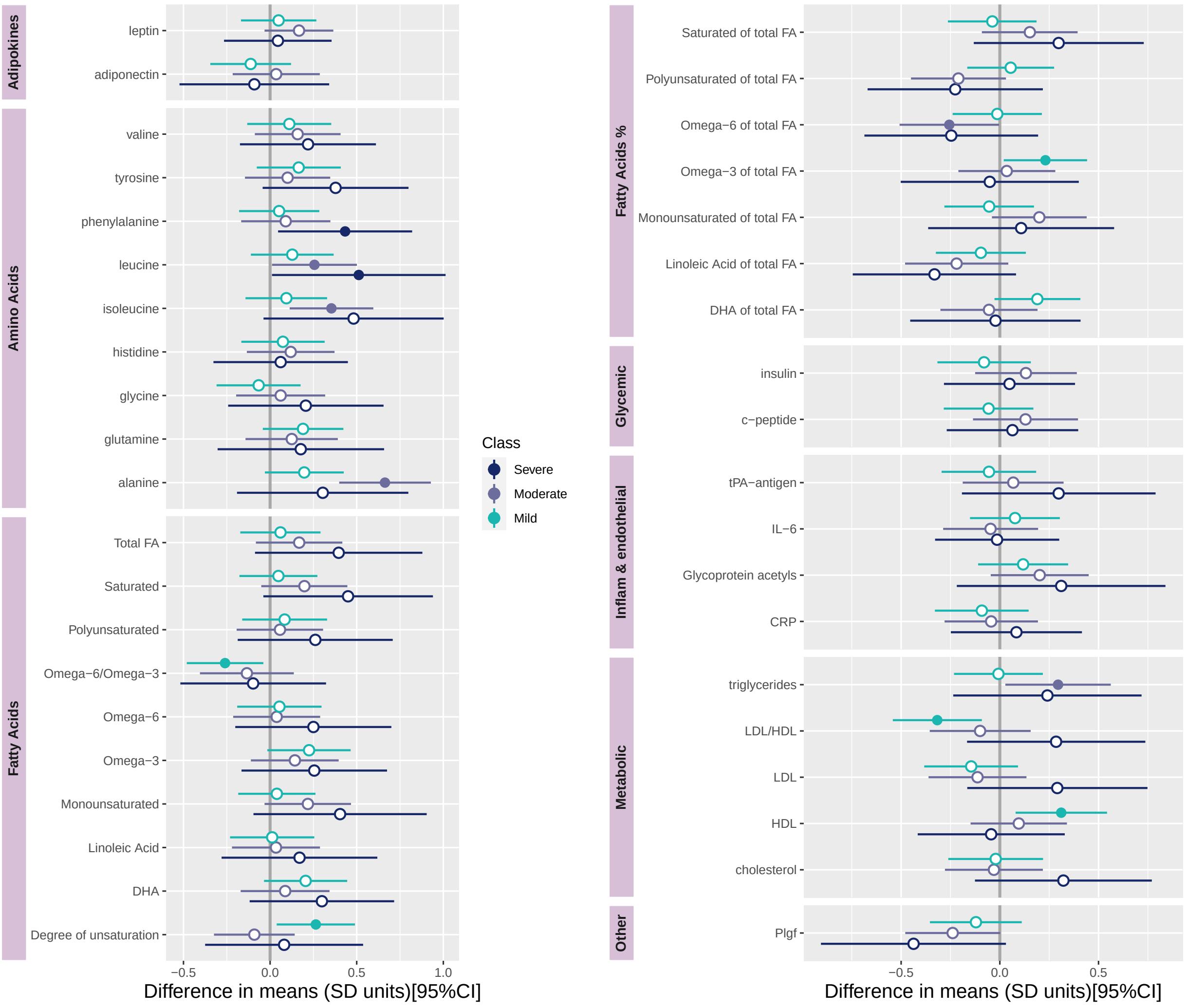

Figure S15: Blood samples at 27 weeks were analysed excluding 120 participants from one centre from which 1h post OGTT research samples were analysed rather than the fasting sample (0h) as per the UPBEAT study protocol. Mean standardized differences are presented after adjustement for maternal age, ethnicity(white vs non-white), nulliparity, latent obesity, latent SES and intervention.

5.4.7 Tables at 34 weeks

Table S29: Mean differences in Adipokines Inflammation and endothelial function, Amino Acids, Placental, Glycaemic markers against the Not Depressed class in standardised units at 34 weeks gestation.

		Severe		Moderate		Mild	
Group	Biomarker	MD[95%CI]	adjMD[95%CI]	MD[95%CI]	adjMD[95%CI]	MD[95%CI]	adjMD[95%CI]
Adipokines	adiponectin	-0.42[-0.94 to 0.10]	-0.23[-0.76 to 0.30]	-0.02[-0.30 to 0.25]	0.12[-0.14 to 0.39]	-0.10 [-0.34 to 0.13]	-0.07[-0.30 to 0.15]
Amino Acids	alanine	0.27[-0.18 to 0.72]	0.45[0.00 to 0.90]	0.13[-0.17 to 0.43]	0.22[-0.07 to 0.52]	0.05 [-0.18 to 0.28]	0.07[-0.16 to 0.30]
	glutamine	-0.25[-0.78 to 0.28]	-0.23[-0.76 to 0.29]	0.03[-0.26 to 0.32]	0.09[-0.19 to 0.37]	0.15 [-0.08 to 0.38]	0.19[-0.04 to 0.42]
	glycine	-0.11[-0.66 to 0.43]	-0.05[-0.59 to 0.48]	-0.08[-0.34 to 0.18]	-0.03[-0.30 to 0.23]	-0.11 [-0.35 to 0.13]	-0.10[-0.35 to 0.14]
	histidine	-0.38[-0.93 to 0.17]	-0.26[-0.78 to 0.25]	0.04[-0.23 to 0.31]	0.11[-0.15 to 0.37]	0.12 [-0.12 to 0.36]	0.16[-0.08 to 0.39]
	isoleucine	0.06[-0.52 to 0.64]	0.12[-0.47 to 0.71]	0.16[-0.11 to 0.42]	0.19[-0.07 to 0.46]	0.15 [-0.09 to 0.39]	0.16[-0.09 to 0.40]
	leucine	-0.20[-0.73 to 0.34]	-0.09[-0.63 to 0.46]	0.09[-0.17 to 0.36]	0.16[-0.09 to 0.42]	0.15 [-0.09 to 0.38]	0.14[-0.10 to 0.38]
	phenylalanine	-0.35[-0.82 to 0.12]	-0.17[-0.60 to 0.25]	-0.04[-0.33 to 0.24]	0.09[-0.18 to 0.36]	-0.06 [-0.30 to 0.17]	-0.07[-0.30 to 0.16]
	tyrosine	-0.28[-0.72 to 0.16]	-0.18[-0.64 to 0.29]	-0.06[-0.30 to 0.19]	0.00[-0.25 to 0.25]	0.17 [-0.07 to 0.42]	0.14[-0.10 to 0.39]
	valine	-0.30[-0.85 to 0.26]	-0.23[-0.81 to 0.36]	0.07[-0.18 to 0.32]	0.10[-0.15 to 0.35]	0.23 [-0.01 to 0.47]	0.21[-0.04 to 0.45]
Glycaemic markers	c-peptide	0.32[-0.17 to 0.81]	0.44[-0.07 to 0.94]	-0.19[-0.47 to 0.09]	-0.15[-0.42 to 0.11]	-0.10 [-0.33 to 0.13]	-0.09[-0.33 to 0.15]
	glucose	0.62[0.04 to 1.21]	0.67[0.07 to 1.26]	0.02[-0.28 to 0.32]	0.02[-0.28 to 0.32]	-0.08 [-0.30 to 0.14]	-0.07[-0.30 to 0.15]
	insulin	0.01[-0.64 to 0.65]	0.04[-0.59 to 0.67]	-0.11[-0.38 to 0.16]	-0.13[-0.40 to 0.14]	-0.06 [-0.30 to 0.17]	-0.07[-0.31 to 0.17]
Inflammation and endothelial function	CRP	-0.07[-0.52 to 0.38]	-0.09[-0.52 to 0.34]	-0.01[-0.27 to 0.25]	0.00[-0.26 to 0.26]	-0.01 [-0.25 to 0.22]	0.05[-0.19 to 0.29]
	Glycoprotein acetyls	0.37[-0.16 to 0.90]	0.42[-0.14 to 0.98]	0.14[-0.16 to 0.44]	0.17[-0.12 to 0.46]	0.20 [-0.04 to 0.42]	0.24[0.01 to 0.48]

Note:
AdjMD=adjusted mean difference, all presented in SD units, with maternal age, nulliparity (vs multiparity), white ethnicity (vs other), latent socio-economic status, latent obesity and intervention as covariates.

Table S30: Mean differences in Fatty Acids, Fatty Acids (of Total Fatty acids) and Metabolic markers against the Not Depressed class in standardised units at 34 weeks gestation.

		Severe		Moderate		Mild	
Group	Biomarker	MD[95%CI]	adjMD[95%CI]	MD[95%CI]	adjMD[95%CI]	MD[95%CI]	adjMD[95%CI]
Fatty Acids	DHA	0.31[-0.22 to 0.84]	0.32[-0.20 to 0.85]	0.07[-0.19 to 0.34]	0.04[-0.23 to 0.31]	0.19 [-0.05 to 0.43]	0.21[-0.04 to 0.45]
	Omega-3	0.42[-0.07 to 0.91]	0.45[-0.04 to 0.94]	0.09[-0.17 to 0.36]	0.07[-0.20 to 0.34]	0.18 [-0.05 to 0.42]	0.20[-0.05 to 0.44]
	Omega-6	0.19[-0.32 to 0.70]	0.22[-0.31 to 0.74]	-0.04[-0.32 to 0.23]	-0.04[-0.31 to 0.24]	0.07 [-0.17 to 0.30]	0.09[-0.16 to 0.33]
	Linoleic Acid	0.15[-0.34 to 0.65]	0.15[-0.36 to 0.66]	-0.05[-0.32 to 0.22]	-0.05[-0.33 to 0.22]	0.06 [-0.18 to 0.29]	0.07[-0.17 to 0.32]
	Monounsaturated	0.28[-0.25 to 0.82]	0.38[-0.16 to 0.93]	0.06[-0.25 to 0.37]	0.16[-0.12 to 0.44]	-0.02 [-0.25 to 0.21]	0.04[-0.18 to 0.26]
	Polyunsaturated	0.23[-0.28 to 0.74]	0.26[-0.26 to 0.78]	-0.02[-0.30 to 0.25]	-0.02[-0.29 to 0.25]	0.09 [-0.15 to 0.32]	0.11[-0.14 to 0.35]
	Saturated	0.31[-0.25 to 0.87]	0.44[-0.14 to 1.02]	0.03[-0.28 to 0.33]	0.14[-0.15 to 0.42]	-0.05 [-0.28 to 0.17]	-0.02[-0.24 to 0.20]
	Total FA	0.30[-0.26 to 0.85]	0.40[-0.17 to 0.97]	0.03[-0.27 to 0.32]	0.10[-0.18 to 0.38]	0.00 [-0.23 to 0.23]	0.04[-0.19 to 0.27]
	Degree of unsaturation	0.13[-0.25 to 0.50]	-0.03[-0.43 to 0.38]	0.00[-0.28 to 0.28]	-0.15[-0.40 to 0.11]	0.24 [0.00 to 0.48]	0.23[0.01 to 0.45]
	Omega-6/Omega-3	-0.42[-0.79 to -0.05]	-0.46[-0.86 to -0.07]	-0.17[-0.45 to 0.10]	-0.14[-0.42 to 0.14]	-0.17 [-0.41 to 0.07]	-0.16[-0.40 to 0.08]
Fatty Acids %	DHA of total FA	0.07[-0.34 to 0.48]	-0.02[-0.47 to 0.43]	0.13[-0.15 to 0.41]	0.01[-0.26 to 0.27]	0.23 [-0.01 to 0.47]	0.20[-0.02 to 0.43]
	Omega-3 of total FA	0.24[-0.16 to 0.65]	0.16[-0.29 to 0.61]	0.16[-0.13 to 0.45]	0.03[-0.23 to 0.30]	0.24 [0.01 to 0.48]	0.21[-0.01 to 0.43]
	Omega-6 of total FA	-0.18[-0.58 to 0.23]	-0.31[-0.70 to 0.07]	-0.04[-0.35 to 0.26]	-0.18[-0.45 to 0.10]	0.13 [-0.10 to 0.36]	0.08[-0.14 to 0.30]
	Linoleic Acid of total FA	-0.20[-0.61 to 0.21]	-0.36[-0.72 to 0.00]	-0.05[-0.35 to 0.24]	-0.19[-0.47 to 0.08]	0.10 [-0.14 to 0.33]	0.05[-0.17 to 0.27]
	Monounsaturated of total FA	0.09[-0.33 to 0.51]	0.16[-0.26 to 0.58]	0.03[-0.28 to 0.34]	0.14[-0.12 to 0.41]	-0.07 [-0.30 to 0.16]	0.01[-0.20 to 0.22]
	Polyunsaturated of total FA	-0.10[-0.51 to 0.31]	-0.24[-0.65 to 0.17]	0.00[-0.30 to 0.30]	-0.15[-0.41 to 0.12]	0.17 [-0.06 to 0.40]	0.12[-0.09 to 0.33]
	Saturated of total FA	0.08[-0.32 to 0.48]	0.27[-0.14 to 0.68]	-0.05[-0.33 to 0.23]	0.10[-0.17 to 0.37]	-0.24 [-0.48 to 0.00]	-0.26[-0.49 to -0.03]
Metabolic	cholesterol	0.18[-0.38 to 0.74]	0.30[-0.25 to 0.85]	-0.08[-0.36 to 0.20]	0.00[-0.27 to 0.27]	-0.06 [-0.29 to 0.17]	-0.03[-0.26 to 0.21]
	LDL	0.11[-0.46 to 0.68]	0.18[-0.36 to 0.73]	-0.15[-0.43 to 0.13]	-0.09[-0.36 to 0.19]	-0.15 [-0.38 to 0.08]	-0.11[-0.34 to 0.13]
	LDL/HDL	0.07[-0.51 to 0.64]	0.05[-0.48 to 0.59]	-0.09[-0.39 to 0.22]	-0.05[-0.35 to 0.24]	-0.29 [-0.52 to -0.07]	-0.23[-0.46 to 0.00]
	triglycerides	0.20[-0.34 to 0.73]	0.28[-0.27 to 0.83]	0.16[-0.16 to 0.48]	0.23[-0.08 to 0.54]	-0.08 [-0.30 to 0.14]	-0.04[-0.26 to 0.18]

Note:
AdjMD= adjusted mean difference, all presented in SD units, with maternal age, nulliparity (vs multiparity), white ethnicity (vs other), latent socio-economic status, latent obesity and intervention as covariates.

5.5 Group comparison on reports of infections prior to each visit

Table S31: Class comparison on reported infections at each visit.

		17 weeks n= 1344			27 weeks n=1179			34 weeks n=1007		
		Prob (%)	OR[95%CI]	adjOR[95%CI]	Prob (%)	OR[95%CI]	adjOR[95%CI]	Prob (%)	OR[95%CI]	adjOR[95%CI]
Severe	No infection	53.50	ref	ref	59.40	ref	ref	67.00	ref	ref
	Infection	46.50	3.11 [1.74 to 5.56]	3.11 [1.61 to 6.00]	40.60	2.37 [1.21 to 4.62]	2.18 [1.06 to 4.49]	33.00	2.51 [1.18 to 5.34]	2.15 [0.89 to 5.19]
Moderate	No infection	74.90	ref	ref	68.70	ref	ref	68.40	ref	ref
	Infection	25.10	1.20 [0.76 to 1.87]	1.34 [0.84 to 2.12]	31.30	1.58 [0.93 to 2.67]	1.72 [1.07 to 2.76]	31.60	2.35 [1.47 to 3.76]	2.20 [1.29 to 3.77]
Mild	No infection	69.20	ref	ref	71.60	ref	ref	73.90	ref	ref
	Infection	30.80	1.59 [1.02 to 2.47]	1.40 [0.93 to 2.09]	28.40	1.38 [0.95 to 2.01]	1.52 [0.98 to 2.37]	26.10	1.80 [1.17 to 2.78]	1.94 [1.17 to 3.23]
Not Depressed	No infection	78.10	ref	ref	77.60	ref	ref	83.60	ref	ref
	Infection	21.90	ref	ref	22.40	ref	ref	16.40	ref	ref

Note:
Women were asked to report any infections prior to the first visit, between the first and the second visit and between the second and the third visit. Classes Odds ratios (ORs) and confidence intervals (CIs) are presented against the Not Depressed class as the reference class. Adjustment for maternal age, nulliparity, white (vs non-white) ethnicity, latent SES, latent obesity and the intervention (at 27 and 34 weeks only).

5.6 Group comparisons on gestational weight gain

Table S32: Adjusted mean gestational weight gain across classes.

	Unadjusted		Adjusted	
Class	Mean GWG (kg)[95%CI]	Difference(kg) [95%CI]	Mean GWG (kg)[95%CI]	Difference(kg) [95%CI]
Severe	9.00[6.99 to 11.01]	1.71[-0.37 to 3.78]	12.45[9.64 to 15.26]	2.64[0.63 to 4.64]
Moderate	7.77[6.77 to 8.77]	0.47[-0.62 to 1.56]	10.70[8.65 to 12.74]	0.88[-0.16 to 1.93]
Mild	7.46[6.87 to 8.05]	0.16[-0.72 to 1.04]	9.66[7.71 to 11.61]	-0.16[-1.02 to 0.70]
Not Depressed	7.30[6.80 to 7.79]		9.81[7.93 to 11.70]	

Note:
Mean Gestational weight gain (GWG) at 34 weeks and difference in GWG against the Not Depressed class, after adjustment for maternal age, parity, white ethnicity, SES, Obesity, Intervention.

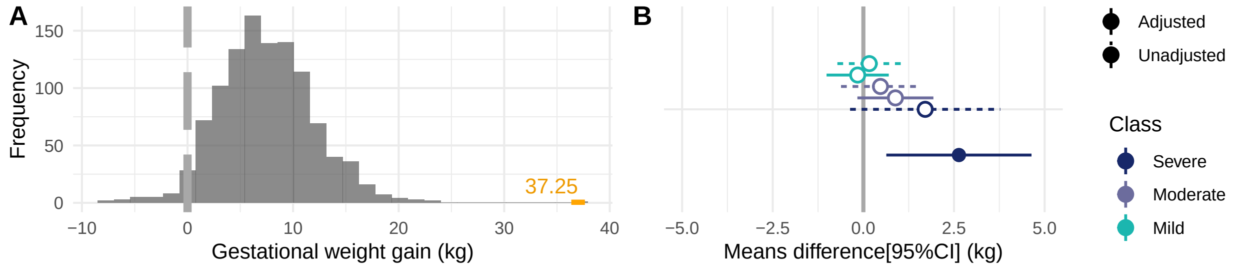

Figure S16: Gestational weight gain at 34 weeks. A) Distribution of weight gains (kg) shows outlier value 37.25kg which is excluded in further analyses. C) Plot representing the mean difference in GWG against the Not Depressed class after adjustement for maternal age, parity, white ethnicity, latent SES, latent obesity and intervention, bars represent the 95%CI. Available data n=1004.

5.7 Pregnancy outcomes

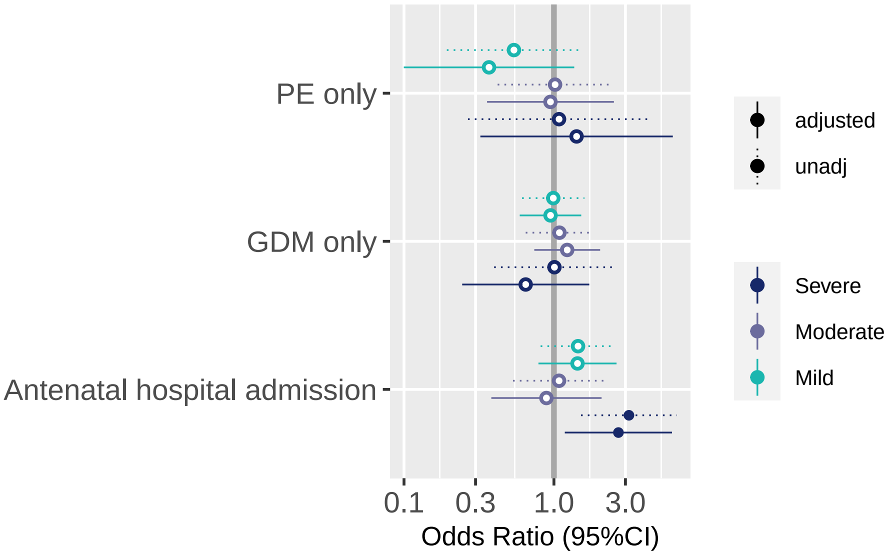

Figure S17: ORs and 95%CIs are plotted for outcomes during pregnancy with the Not Depressed class as the reference. Participants with only either GDM or PE were included (i.e., excluded participants with superimposed GDM with PE). Covariates in adjusted models included maternal age, nulliparity, white ethnicity, latent SES, latent Obesity and intervention. GDM= gestational diabetes mellitus, PE=Pre-eclampsia.

Table S33: Class comparison on obstetric complications and admission to hospital.

		Not Depressed	Severe			Moderate			Mild		
		Prob (%)	Prob (%)	OR[95%CI]	adjOR[95%CI]	Prob (%)	OR[95%CI]	adjOR[95%CI]	Prob (%)	OR[95%CI]	adjOR[95%CI]
Antenatal hospital admission	No	92.40	79.40	ref	ref	91.80	ref	ref	89.40	ref	ref
	Yes	7.60	20.60	3.16 [1.51 to 6.61]	2.69 [1.18 to 6.13]	8.20	1.08 [0.53 to 2.20]	0.89 [0.38 to 2.08]	10.60	1.45 [0.81 to 2.57]	1.44 [0.79 to 2.62]
GDM only	no GDM	78.40	78.30	ref	ref	77.00	ref	ref	78.60	ref	ref
	GDM	21.60	21.70	1.01 [0.40 to 2.55]	0.65 [0.24 to 1.72]	23.00	1.09 [0.65 to 1.82]	1.23 [0.74 to 2.03]	21.40	0.99 [0.61 to 1.60]	0.95 [0.59 to 1.52]
PE only	no PE	95.40	95.00	ref	ref	95.30	ref	ref	97.40	ref	ref
	PE	4.60	5.00	1.08 [0.27 to 4.38]	1.42 [0.32 to 6.21]	4.70	1.02 [0.42 to 2.46]	0.95 [0.36 to 2.51]	2.60	0.54 [0.19 to 1.51]	0.37 [0.10 to 1.37]

Note:
Classes Odds ratios (ORs) and confidence intervals (CIs) are presented against the Not Depressed class as the reference class. Participants excluded if they experienced superimposed obstetric comorbidities.

5.8 Birth outcomes

Table S34: Class comparison on birth outcomes.

		Not Depressed	Severe			Moderate			Mild		
		Prob (%)	Prob (%)	OR[95%CI]	adjOR[95%CI]	Prob (%)	OR[95%CI]	adjOR[95%CI]	Prob (%)	OR[95%CI]	adjOR[95%CI]
Preterm	Born at term	97.10	85.40	ref	ref	94.60	ref	ref	91.60	ref	ref
	Born <37 weeks	2.90	14.60	5.71 [2.06 to 15.85]	3.05 [1.11 to 8.36]	5.40	1.90 [0.71 to 5.12]	1.56 [0.66 to 3.71]	8.40	3.06 [1.22 to 7.72]	1.84 [0.81 to 4.18]
LGA 90% by WHO	No	85.30	85.80	ref	ref	89.50	ref	ref	91.90	ref	ref
	Yes	14.70	14.20	0.96 [0.41 to 2.24]	0.90 [0.32 to 2.47]	10.50	0.68 [0.37 to 1.24]	0.90 [0.49 to 1.66]	8.10	0.51 [0.30 to 0.88]	0.54 [0.29 to 1.02]
IOL	Not induced	63.80	70.00	ref	ref	60.30	ref	ref	68.70	ref	ref
	Induced	36.20	30.00	0.76 [0.36 to 1.58]	0.79 [0.39 to 1.60]	39.70	1.16 [0.78 to 1.73]	1.20 [0.79 to 1.81]	31.30	0.80 [0.57 to 1.14]	0.86 [0.58 to 1.26]
Blood loss >1000mls	<1000ml	86.60	91.30	ref	ref	89.30	ref	ref	84.40	ref	ref
	>=1000ml	13.40	8.70	0.62 [0.19 to 1.98]	0.74 [0.23 to 2.34]	10.70	0.78 [0.39 to 1.55]	0.83 [0.42 to 1.61]	15.60	1.20 [0.76 to 1.90]	1.15 [0.69 to 1.90]
SGA 10% by WHO	Not SGA	93.40	94.60	ref	ref	92.10	ref	ref	95.30	ref	ref
	SGA	6.60	5.40	0.80 [0.22 to 2.87]	0.77 [0.18 to 3.24]	7.90	1.21 [0.62 to 2.38]	1.16 [0.58 to 2.33]	4.70	0.69 [0.32 to 1.51]	0.64 [0.28 to 1.47]
NICU	Not admitted	93.20	93.70	ref	ref	93.30	ref	ref	91.20	ref	ref
	Admitted	6.80	6.30	0.91 [0.27 to 3.05]	0.79 [0.20 to 3.11]	6.70	0.97 [0.46 to 2.08]	0.97 [0.42 to 2.24]	8.80	1.31 [0.72 to 2.41]	1.29 [0.66 to 2.52]
Spont PTB/PROM	No	97.90	90.90	ref	ref	97.60	ref	ref	93.50	ref	ref
	Yes	2.10	9.10	4.63 [1.36 to 15.72]	2.83 [0.87 to 9.17]	2.40	1.11 [0.27 to 4.52]	1.01 [0.28 to 3.61]	6.50	3.19 [1.11 to 9.22]	1.93 [0.77 to 4.81]
CS all	No CS	64.00	71.50	ref	ref	62.50	ref	ref	64.10	ref	ref
	CS	36.00	28.50	0.71 [0.32 to 1.58]	0.69 [0.32 to 1.46]	37.50	1.06 [0.73 to 1.56]	1.10 [0.72 to 1.68]	35.90	1.00[0.71 to 1.39]	1.11[0.76 to 1.61]

Note:
Classes Odds ratios (ORs) and confidence intervals (CIs) are presented against the Not Depressed class as the reference class. IOL: Induction of Labour, NICU: Neonatal Intensive Care Unit. PROM: Premature Rupture of Membrane, PTB: Preterm birth, SGA/LGA: Small/large for gestational age.

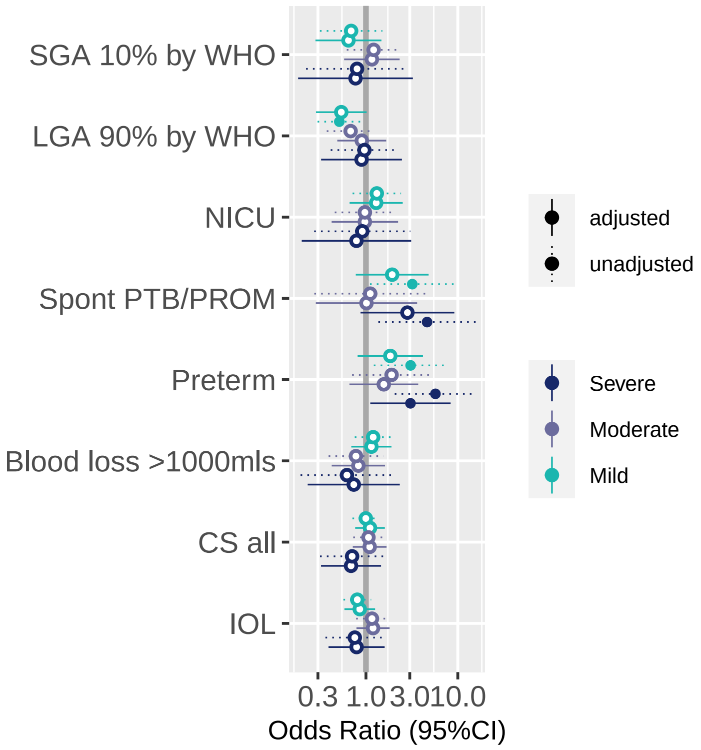

Figure S18: Odds Ratios (95%CIs) obtained from logistic regressions on birth outcomes are plotted where the Not Depressed class is the reference and adjusted models included maternal age, white ethnicity,nulliparity, latent SES, latent Obesity and intervention were covariates. Filled circles indicate CIs excluded 1 and is interpreted as significant. IOL: Induction of Labour, NICU: Neonatal Intensive Care Unit. PROM: Premature Rupture of Membrane, PTB: Preterm birth, SGA/LGA: Small/large for gestational age.

Table S35: Adjusted mean gestational age at birth (days) and difference.

	Unadjusted		Adjusted	
Class	Mean GA at birth (days)	Difference (days)[95%CI]	Mean GA at birth (days)	Difference (days)[95%CI]
Severe	274.24[270.11 to 278.36]	-3.85[-8.20 to 0.49]	287.413[279.903 to 294.923]	-1.85[-6.37 to 2.67]
Moderate	275.23[272.42 to 278.04]	-2.86[-5.89 to 0.17]	287.262[281.753 to 292.771]	-2.00[-5.04 to 1.04]
Mild	276.94[275.30 to 278.59]	-1.15[-3.55 to 1.25]	288.019[281.794 to 294.243]	-1.25[-3.63 to 1.14]
Not Depressed	278.09[276.77 to 279.41]		289.263[283.552 to 294.975]	

Note:
Gestational age at birth and difference against the Not Depressed class, after adjustment for maternal age, parity, white ethnicity, SES, Obesity, intervention.

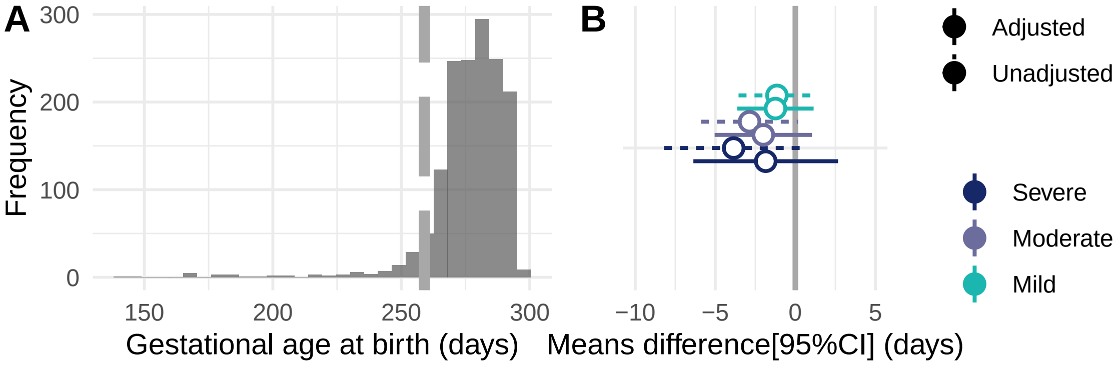

Figure S19: A. Gestational ages at delivery. B. Mean differences in unadjusted comparisons and adjusted for age, nulliparity, white ethnicity, intervention, latent socio-economic status (SES), latent obesity.

References

1 Poston L, Bell R, Croker H, *et al.* Effect of a behavioural intervention in obese pregnant women (the UPBEAT study): a multicentre, randomised controlled trial. *The Lancet Diabetes & Endocrinology* 2015; **3**: 767–77.

2 White SL, Pasupathy D, Sattar N, *et al.* Metabolic profiling of gestational diabetes in obese women during pregnancy. *Diabetologia* 2017; **60**: 1903–12.

3 Report of the National High Blood Pressure Education Program Working Group on High Blood Pressure in Pregnancy. *American Journal of Obstetrics and Gynecology* 2000; **183**: s1–22.

4 Vieira MC, Begum S, Seed PT, *et al.* Gestational diabetes modifies the association between PlGF in early pregnancy and preeclampsia in women with obesity. *Pregnancy Hypertension* 2018; **13**: 267–72.

5 Baron E, Bass J, Murray SM, Schneider M, Lund C. A systematic review of growth curve mixture modelling literature investigating trajectories of perinatal depressive symptoms and associated risk factors. *Journal of Affective Disorders* 2017; **223**: 194–208.

6 Akaike H. Factor analysis and AIC. *Psychometrika* 1987; **52**: 317–32.

7 Asparouhov T, Muthén B. Auxiliary Variables in Mixture Modeling: Three-Step Approaches Using M plus. *Structural Equation Modeling: A Multidisciplinary Journal* 2014; **21**: 329–41.

8 Lo Y. Testing the number of components in a normal mixture. *Biometrika* 2001; **88**: 767–78.

9 Wickrama, K. K., Lee, T. K., O'Neal, C. W., & Lorenz FO. Higher-Order Growth Curves and Mixture Modeling with Mplus. Routledge, 2016 DOI:10.4324/9781315642741.

10 Schoot R van de, Sijbrandij M, Winter SD, Depaoli S, Vermunt JK. The GRoLTS-Checklist: Guidelines for Reporting on Latent Trajectory Studies. *Structural Equation Modeling: A Multidisciplinary Journal* 2017; **24**: 451–67.

11 Bakk Z, Vermunt JK. Robustness of Stepwise Latent Class Modeling With Continuous Distal Outcomes. *Structural Equation Modeling: A Multidisciplinary Journal* 2016; **23**: 20–31.

12 Nylund-Gibson K, Grimm RP, Masyn KE. Prediction from Latent Classes: A Demonstration of Different Approaches to Include Distal Outcomes in Mixture Models. *Structural Equation Modeling* 2019; **26**: 967–85.

13 Nylund-Gibson K, Masyn KE. Covariates and Mixture Modeling: Results of a Simulation Study Exploring the Impact of Misspecified Effects on Class Enumeration. *Structural Equation Modeling: A Multidisciplinary Journal* 2016; **23**: 782–97.

14 Asparouhov T, Muthén B. Auxiliary variables in mixture modeling: Using the BCH method in Mplus to estimate a distal outcome model and an arbitrary second model. *Mplus Web Notes* 2021; : 1–80.
